# Systems biology analysis of the proteomic alterations induced by MPP^+^, a Parkinson's disease-related mitochondrial toxin

**DOI:** 10.3389/fncel.2015.00014

**Published:** 2015-02-02

**Authors:** Chiara Monti, Heather Bondi, Andrea Urbani, Mauro Fasano, Tiziana Alberio

**Affiliations:** ^1^Biomedical Research Division, Department of Theoretical and Applied Sciences, University of InsubriaBusto Arsizio, Italy; ^2^Center of Neuroscience, University of InsubriaBusto Arsizio, Italy; ^3^Santa Lucia IRCCS FoundationRome, Italy; ^4^Department of Experimental Medicine and Surgery, University of Rome “Tor Vergata,”Rome, Italy

**Keywords:** MPP^+^, systems biology, meta-analysis, network enrichment, over-representation analysis

## Abstract

Parkinson's disease (PD) is a complex neurodegenerative disease whose etiology has not been completely characterized. Many cellular processes have been proposed to play a role in the neuronal damage and loss: defects in the proteosomal activity, altered protein processing, increased reactive oxygen species burden. Among them, the involvement of a decreased activity and an altered disposal of mitochondria is becoming more and more evident. The mitochondrial toxin 1-methyl-4-phenylpyridinium (MPP^+^), an inhibitor of complex I, has been widely used to reproduce biochemical alterations linked to PD *in vitro* and its precursor, 1-methyl-4-phenyl-1,2,3,6-tetrahydropyridine hydrochloride (MPTP), to induce a Parkinson-like syndrome *in vivo*. Therefore, we performed a meta-analysis of the literature of all the proteomic investigations of neuronal alterations due to MPP^+^ treatment and compared it with our results obtained with a mitochondrial proteomic analysis of SH-SY5Y cells treated with MPP^+^. By using open-source bioinformatics tools, we identified the biochemical pathways and the molecular functions mostly affected by MPP^+^, i.e., ATP production, the mitochondrial unfolded stress response, apoptosis, autophagy, and, most importantly, the synapse funcionality. Eventually, we generated protein networks, based on physical or functional interactions, to highlight the relationships among the molecular actors involved. In particular, we identified the mitochondrial protein HSP60 as the central hub in the protein-protein interaction network. As a whole, this analysis clarified the cellular responses to MPP^+^, the specific mitochondrial proteome alterations induced and how this toxic model can recapitulate some pathogenetic events of PD.

## Introduction

Parkinson's disease (PD) is the second most common neurodegenerative disorder. It is pathologically characterized by the loss of dopaminergic neurons in the midbrain *substantia nigra pars compacta* (SNpc) and it is anatomically characterized by the formation of intra-cytoplasmic protein aggregates called Lewy bodies (Dauer and Przedborski, 2003; Keane et al., 2011). The loss of dopaminergic neurons causes the appearance of motor symptoms, such as bradykinesia, resting tremor, rigidity, and postural instability (Calvo and Mootha, 2010). The majority of data on the molecular mechanisms underlying neuronal degeneration comes from studies on animal and cellular models (Alberio et al., 2012; Blandini and Armentero, 2012). Among the biochemical pathways involved in neuronal loss, mitochondrial impairment has been repeatedly demonstrated both in PD patients and PD models (Hauser and Hastings, 2013). Therefore, mitochondrial toxins are often used to generate a specific insult in dopaminergic cells (Yadav et al., 2012).

1-Methyl-4-phenyl-1,2,3,6-tetrahydropyridine hydrochloride (MPTP) was originally observed to reproduce Parkinson's like symptoms in heroin addicts ingesting a synthetic narcotic containing the toxic substance (Davis et al., 1979; Langston and Palfreman, 1996). MPTP is metabolized by monoamine-oxidase (MAO)-B (Brooks et al., 1989) in astrocytes (Sedelis et al., 2000) into an active toxic cation product, 1-methyl-4-phenylpyridinium (MPP^+^), which primarily targets nigrostriatal dopaminergic neurons because it is transported into cells via the dopamine transporter (DAT) (Chagkutip et al., 2003). This leads to inhibition of complex I of the electron transport chain (ETC) (Nicklas et al., 1985; Ramsay et al., 1986) and cell death specifically in dopaminergic neurons. This neurotoxin became of particular interest when it was found to reproduce symptomatic, pathological, and biochemical features of PD in animal models (Tieu, 2011). The active toxic metabolite MPP^+^ is frequently used in *in vitro* studies but does not cross the blood brain barrier, thus the precursor MPTP is used to generate one of the most commonly used pharmacological models of PD.

The animals most frequently used for MPTP-based studies are mice and monkeys. The treatment with MPTP is usually optimized to generate the least number of undesirable consequences (acute death, dehydration, and malnutrition) and, at the same time, the most severe and stable form of SNpc damage (Tieu, 2011). Monkeys are more sensitive to the toxin and often exhibit a generalized (bilateral) parkinsonian syndrome; on the other hand, this model fails to reproduce two important characteristic features of PD: the loss of neurons in other brain monoaminergic areas and the formation of Lewy bodies. Rodents, compared to monkeys, are less sensitive to MPTP toxicity. Nevertheless, the C57black6 mice strain was found to be sensitive to a systemic injection of MPTP and the damage to be selective on mesencephalic dopaminergic neurons. The use of MPTP in rats, on the contrary, is limited because they do not show any significant dopaminergic neurodegeneration (Tieu, 2011). The human neuroblastoma SH-SY5Y cell line (N-type) is widely used for *in vitro* studies. Indeed, these cells express DAT, required for the entry of the toxin into the cell (Alberio et al., 2012).

Current research on the molecular bases of PD is exploiting high-throughput screening techniques to detect altered neuronal gene expression. However, changes in mRNA levels do not always correlate with similar changes in protein levels or activity (Vogel and Marcotte, 2012). Studies evaluating protein expression changes in PD models are therefore crucial. The most common methods of proteome analysis are the comparative two-dimensional electrophoresis (2-DE), followed by mass spectrometry (MS), and shotgun proteomics, based on a gel-free approach (Zhao et al., 2007). Shotgun proteomics shows better sensitivity and resolution than 2-DE, thus allowing the detection and quantification of several hundreds of proteins. However, 2-DE provides a better workbench for the characterization of post-translational modifications, including proteolytic degradation (Alberio et al., 2014). Proteomics is an unbiased approach, aiming at generating a list of candidate proteins deserving further targeted studies. Such a global approach allows to manage with the great complexity of the system being investigated (e.g., the brain), thus overcoming limits of conventional biochemistry or molecular biology tools. However, it is sometimes difficult to interpret a long list of data and solve the false positive and false negative issues. In this regard, the integration of proteomics with bioinformatics data mining methods can unravel the involvement of biochemical pathways that were hidden by the complexity of data themselves and focus the attention on the main mechanisms responsible of the phenomena under investigation (Alberio et al., 2014; Kaever et al., 2014).

In this study we performed a meta-analysis of all the proteomic investigations of neuronal alterations due to the MPP^+^ treatment reported so far (Jin et al., 2005; Zhao et al., 2007; Chin et al., 2008; Diedrich et al., 2008; Liu et al., 2008; Zhang et al., 2010; Burté et al., 2011; Campello et al., 2013; Dixit et al., 2013; Choi et al., 2014) and compared it with our results obtained with a mitochondrial proteomic analysis of SH-SY5Y cells treated with MPP^+^ (Alberio et al., 2014). Through this analysis, we aim at clarifying the cellular responses to MPP^+^, the specific mitochondrial proteome alterations induced and how this toxic model can recapitulate some pathogenetic events of PD.

## Materials and methods

### Input lists generation

Two input lists were generated: the first one -EXP input list- included proteins altered in SH-SY5Y cells treated with MPP^+^ (2.5 mM, 24 h) compared to control cells. Proteomics data were obtained both by 2-DE and shotgun LC-MS/MS (Alberio et al., 2014). The second META input list was obtained by a meta-analysis of the literature by Medline search of “MPP^+^”AND “proteom^*^” or “MPTP^*^”AND “proteom^*^” keywords. All protein IDs were converted to the corresponding human Uniprot ID.

### Bioinformatics analysis

The over-representation analysis (ORA) of protein lists (EXP and META) was carried out using the GO Consortium (http://geneontology.org/), Reactome (http://www.reactome.org/), and Webgestalt online tools (http://bioinfo.vanderbilt.edu/webgestalt/) against Kyoto Encyclopedia of Genes and Genomes (KEGG), WikiPathway and Pathway Commons databases.

The web portal BioProfiling.de was used to build enriched networks. It covers most of the available information regarding signaling and metabolic pathways (database: Reactome) and physical protein-protein interactions (database: IntAct) (Antonov, 2011). Accordingly, the input list was analyzed by Rspider (Haw and Stein, 2012) and PPIspider (Antonov et al., 2009; Orchard et al., 2014) tools, respectively. Both tools employ advanced enrichment or network-based statistical frameworks. The *p*-value provided, computed by Monte Carlo simulation (http://www.bioprofiling.de/statistical_frameworks.html), refers to the probability to get a model of the same quality for a random gene list of the same size (Antonov et al., 2010).

The significant networks D1 (all nodes belong to the input list) and D2 (the insertion of one node between two existing nodes is allowed), *p* < 0.01, were further considered to interpret and discuss proteomics results. The enriched networks were exported as a. xgmml file and visualized and modified by Cytoscape 3.1.0 (http://www.cytoscape.org/) (Shannon et al., 2003).

To perform ORA (GO terms) and network topology analysis of the enriched networks, we used the Jepetto Cytoscape application (Winterhalter et al., 2014; http://apps.cytoscape.org/apps/jepetto), which interacts simultaneously with three servers, i.e., EnrichNet, PathExpand, and TopoGSA. Significantly over-represented GO terms were selected if their Xd score was greater than that calculated as the intercept of the linear regression of Xd scores vs. the Fisher's exact test *p*-value after Benjamini-Hochberg correction (*q*-value) (Glaab et al., 2012). The outcome of the topological analysis considered was the statistics table, providing the averaged values for five topological properties (shortest path length, node betweenness centrality, node degree, clustering coefficient and node eigenvector centrality), and the similarity ranking table, providing a quantitative comparison of the uploaded dataset with the reference datasets, based on a similarity score.

### Subnetwork clustering

ClusterMaker Cytoscape application (http://www.cgl.ucsf.edu/cytoscape/cluster/clusterMaker.shtml) was employed to fragment the META list, PPI D2 model (Morris et al., 2011). In particular, the implemented GLay algorithms were used to identify subnetworks. The GLay environment provides layout algorithms optimized for large networks and allows the exploration and analysis of highly connected biological networks (Su et al., 2010). Eventually, Webgestalt, GO consortium and Reactome were employed to perform ORA of the segmented clusters.

## Results

### Generation of the EXP list and META list

The EXP list was generated by including all the proteins found to be altered in the mitochondrial proteomics investigation of SH-SY5Y exposed to 2.5 mM MPP^+^ for 24 h (Alberio et al., 2014). The list was initially not created nor analyzed in the original paper because in that case MPP^+^ served as an internal control for the dopamine treatment. Fifty-nine proteins detected both by 2-DE and shotgun LC-MS/MS have been included (Supplementary Table 1). For the meta-analysis of the literature, we collected all the valuable information coming from papers where changes at the proteome level were evaluated after MPTP or MPP^+^ treatment (Supplementary Table 1). Data from 11 publications were used to build the list (META input list, 475 proteins). We considered protein changes observed in SH-SY5Y cells (Alberio et al., 2014; Choi et al., 2014), in the N2a mouse neuroblastoma cell line (Burté et al., 2011), in the MPTP-mouse midbrain (Jin et al., 2005; Zhao et al., 2007; Chin et al., 2008; Diedrich et al., 2008; Liu et al., 2008; Zhang et al., 2010; Dixit et al., 2013) and in the MPTP-monkey neuronal retina (Campello et al., 2013).

### Over-representation of biochemical pathways and gene ontology categories

In order to understand which cellular pathways are mainly altered by MPP^+^, both the proteins of our study on SH-SY5Y cells (EXP list) and those found in the literature (META list) were analyzed in terms of ORA by the GO enrichment tool of the GO consortium, by Webgestalt and by Reactome. Webgestalt was used to find over-represented pathways in KEGG, WikiPathways and Pathway Commons databases. All the results coming from the different analyses are detailed in Supplementary Tables 2–8. Since the analyses gave rise to a large amount of results, we selected and summed up the most relevant information in Table 1.

**Table 1:** **Summary table of the most significant results of the GO, Webgestalt, and Reactome analyses**.

	**Input list**	
	**EXP**	**META**
GO biological process	ATP production	
	Response to stress	
	Apoptosis	Synapse
	Mitochondrial transport	Protein complex assembly
	Protein folding/unfolding	Neurotransmitter transport
		Nervous system development
GO molecular function	ATP production	
	Structural constituent of cytoskeleton	
	Voltage-dependent anion channel activity	Phosphatase activity
		Transmembrane transport activity
		SNARE binding
KEGG	Parkinson's disease	
	Oxidative phosphorylation	
	Protein processing	
	Calcium signaling pathway	
		Cytoskeleton
WikiPathways	ATP production	
	Parkin-ubiquitin proteasomal system pathway	
		Synaptic vesicle pathway
		Signaling pathways (EGF, Insulin, TSH, FOS, G13)
		Cytoskeleton
		G protein signaling pathway
Pathway Commons	ATP production	
	Apoptosis	
	Unfolded protein response	
	Metabolism	
		Synapse: neurotransmitters metabolism and release
		Signaling pathways (VEGF, Insulin, junctions, PI3K, and mTOR, etc…)
Reactome	HSP response to stress	
	ATP production	
	Mitochondria protein import	
	EPH signaling	
		GAP junction trafficking
		L1CAM interaction
		Chemical synapse transmission

The “ATP production” pathway served as an internal control, as MPP^+^ directly acts on the complex I of the ETC (Vila and Przedborski, 2003) and it is known to affect ATP production by the mitochondria. Moreover, the ORA analysis revealed that MPP^+^ induces apoptosis, probably through the intrinsic, mitochondrial-dependent pathway, since it was evidenced also by the EXP list analysis. Furthermore, MPP^+^ influenced mitochondrial transport, mainly through Voltage Dependent Anion Channels (VDACs) and protein folding, inducing a chaperone-mediated response. Both analyses pointed out the involvement of the ubiquitin proteasomal system and the role of parkin in responding to mitochondrial stress. Additionally, the analysis of the META list highlighted the influence of the treatment on the functionality of the synapse, both at the structural and at the molecular level. Eventually, several signaling pathways probably involved in the cellular response to the toxin were shown to be over-represented.

### Functional network enrichment

**Figure 1:**
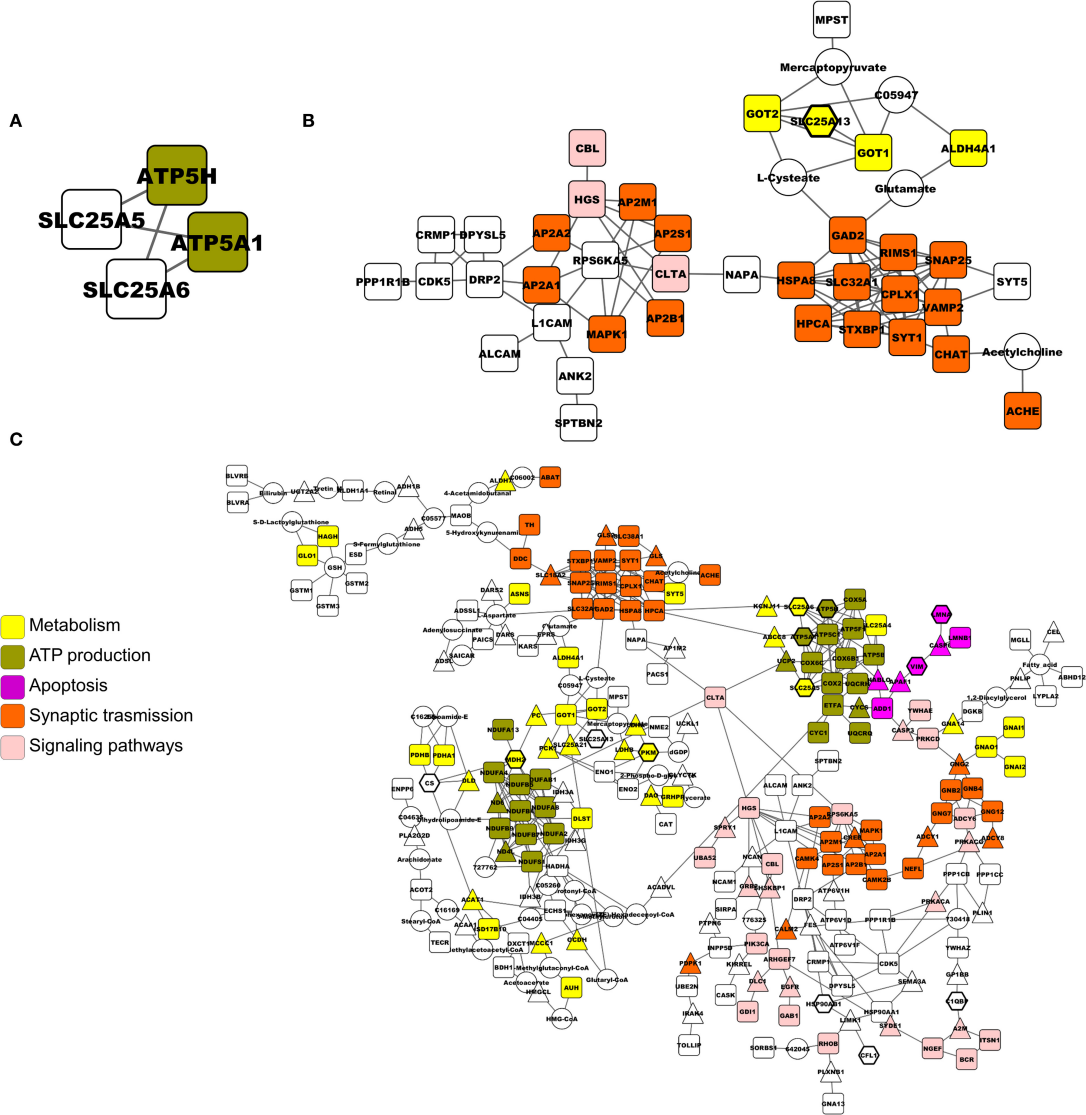
**Networks built by Rspider, using both KEGG and Reactome databases as the reference set. (A)** D1 and D2 model of the EXP list. **(B)** D1 model of the META list. **(C)** D2 model of the META list. Proteins from the input lists are represented by squares. Intermediate genes added by the enrichment tool are represented by triangles. Nodes coming from the EXP list are evidenced by hexagons in **(C)**. Colors indicate gene functional role according to the Gene Ontology.

To visualize which protein networks include the proteins altered by MPP^+^, we performed a network-based analysis of the two lists, using as reference knowledge a global gene network constructed by combining signaling and metabolic pathways from Reactome and KEGG (Rspider). Both D1 model, where all nodes belong to the input list, and D2 model, the enriched network where the insertion of one node between two existing nodes is allowed, generated by the EXP list were superimposable and included only proteins belonging to the ETC (Figure 1A). When all the proteins found to be altered in the literature were included (META list), two more complex networks were generated. Figure 1B shows only proteins of the original list (D1). Orange nodes represent proteins involved in the synaptic transmission, revealing once again the MPP^+^ influence on synaptic functionality. The same proteins, together with others probably involved in the dysfunction at the synapse level, were present also in the D2 model (Figure 1C, orange nodes). Moreover, putative molecular factors responsible of guiding the cellular response (signaling pathways) and the apoptotic process are highlighted in pink and purple, respectively. The hexagonal nodes represent proteins of the EXP list, which contribute to the network built with the META list.

### Physical-interactions network enrichment

As for the functional network analysis, we used a network-based enrichment to visualize physical interactions among proteins of the lists, using the IntAct database as the reference set (PPIspider).

**Figure 2:**
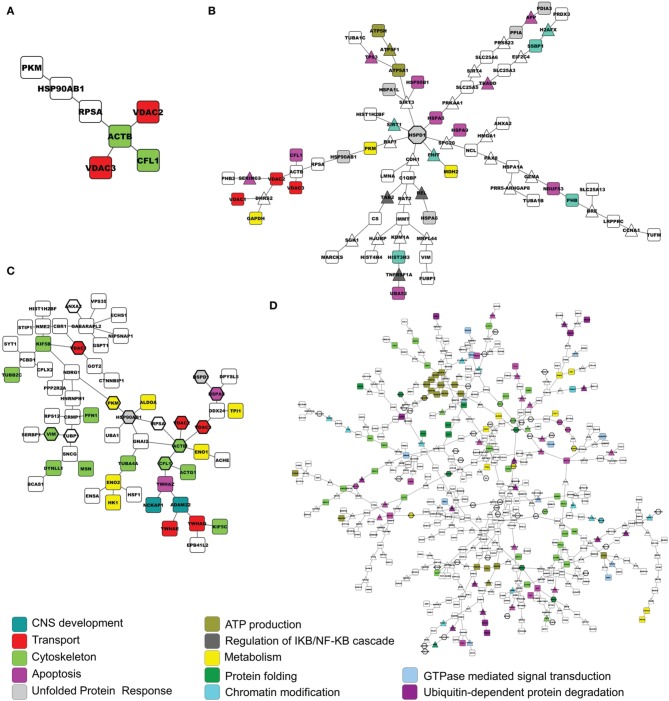
**Networks built by PPIspider, using the IntAct database as the reference set. (A)** D1 model of the EXP list. **(B)** D2 model of the EXP list. **(C)** D1 model of the META list. **(D)** D2 model of the META list. Proteins from the input lists are represented by squares. Intermediate genes added by the enrichment tool are represented by triangles. Nodes coming from the EXP list are evidenced by hexagons in **(C,D)**. Colors indicate gene functional role according to the Gene Ontology.

Figure 2A shows the D1 model for the EXP list. The network evidenced the alteration of proteins involved in mitochondrial transport (red nodes), mainly guided by VDACs. By adding nodes in the D2 model, it was possible to identify HSPD1, alias HSP60, (octagonal node) as a hub, i.e., a central protein directing the mitochondrial response to stress (Figure 2B). This visual representation of physical interactions among proteins responding to MPP^+^ suggests that the unfolded protein response, which is clearly activated, is probably guided by HSP60.

Figure 2C shows the D1 model for the META list. The contribution of proteins by our mitochondrial proteomics investigation is underlined by hexagonal nodes. This network drew attention to cytoskeletal proteins perturbed by the specific challenge (light green nodes). The D2 model of the META list (Figure 2D) was particularly complex and significant protein subgroups were estimated by a specific segmentation analysis. Color code is maintained to show processes already present in the other networks.

### Integrated networks analysis

The list of proteins coming from the D2 network of the EXP list and the D1 network of the META list were analyzed by Jepetto, a Cytoscape app querying the EnrichNet and the PathExpand servers. Results for the analysis of the EXP list, PPI D2 model are summarized in Table 2. The previous enrichment performed by the network-based analysis allowed us to reveal other processes possibly involved, such as the mTOR signaling cascade and the regulation of the DNA repair, beyond other already identified pathways (e.g., ATP production, Response to unfolded protein).

**Table 2:**
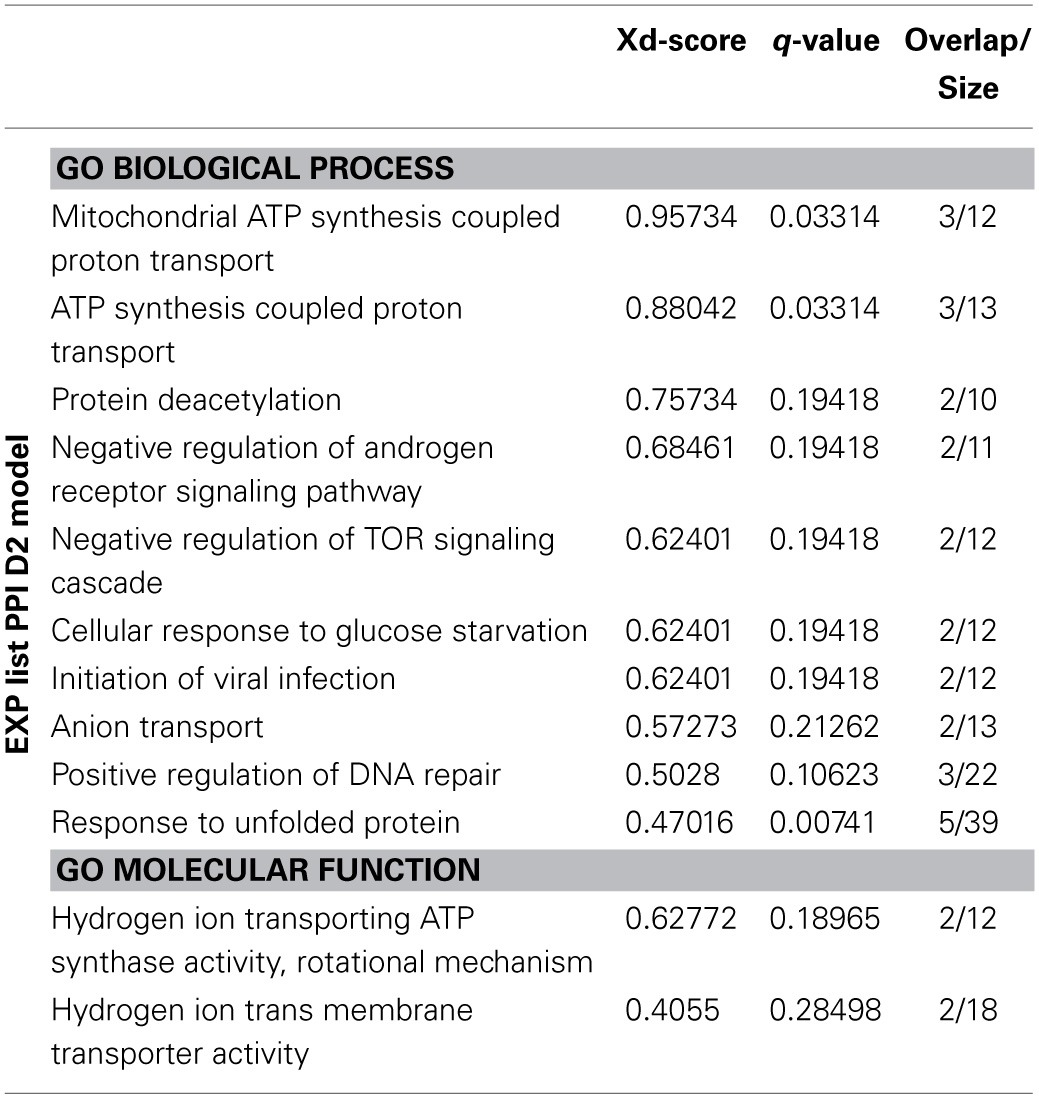
**Summary table of most significant results of the Jepetto analysis of EXP list, PPI D2 model**.

On the other side, the analysis of the PPI D1 model of the META list (non-enriched model) allowed us to obtain a statistical representation of information present in the network. Results are shown in Table 3. The significant results included once again the over-representation of proteins acting at the synapse and implicated in the transport to mitochondria, but also highlighted new pathways, such as autophagy. The topological analysis of this model suggested a close interaction between nodes (Table 4). The average shortest path length was smaller than in random networks, which indicates closer interactions. This was confirmed by over two times higher average node degree.

**Table 3:**
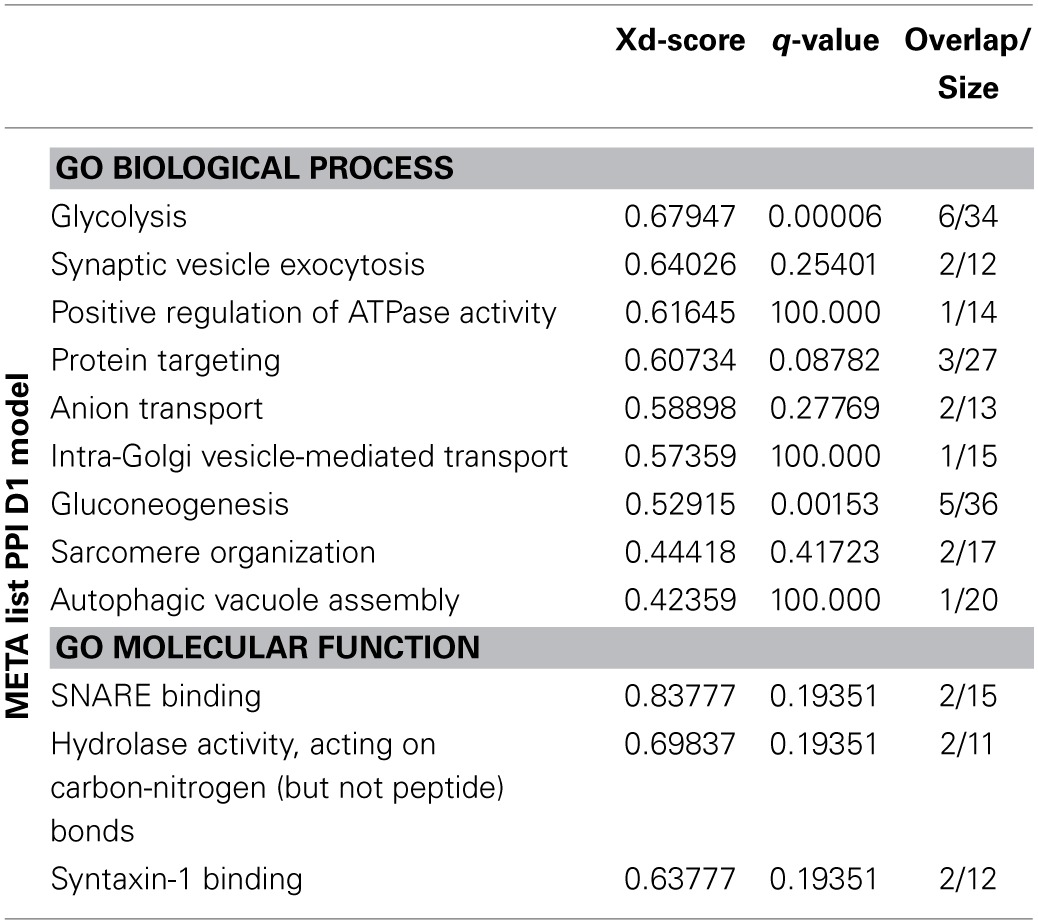
**Summary table of most significant results of the Jepetto analysis of META list, PPI D1 model**.

**Table 4:**
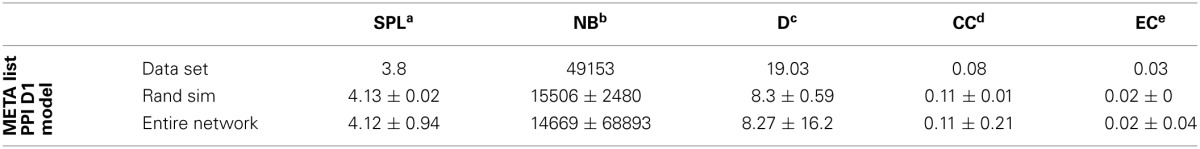
**Topological analysis of the META list, PPI D1 model, using TopoGSA**.

^a^SPL, shortest path length.

^b^NB, node betweenness centrality.

^c^D, node degree.

^d^CC, clustering coefficient.

^e^EC, node eigenvector centrality.

### Subnetwork clustering

Since the PPI D2 model for the META list was very complex, we firstly segmented it into subnetworks using the GLay community detection algorithms. Secondly, we performed ORA on the proteins of each cluster, using the same tools as in the previous analyses (i.e., GO consortium, Webgestalt, Reactome), in order to recognize the univocal biological meaning of each topological cluster.

**Figure 3:**
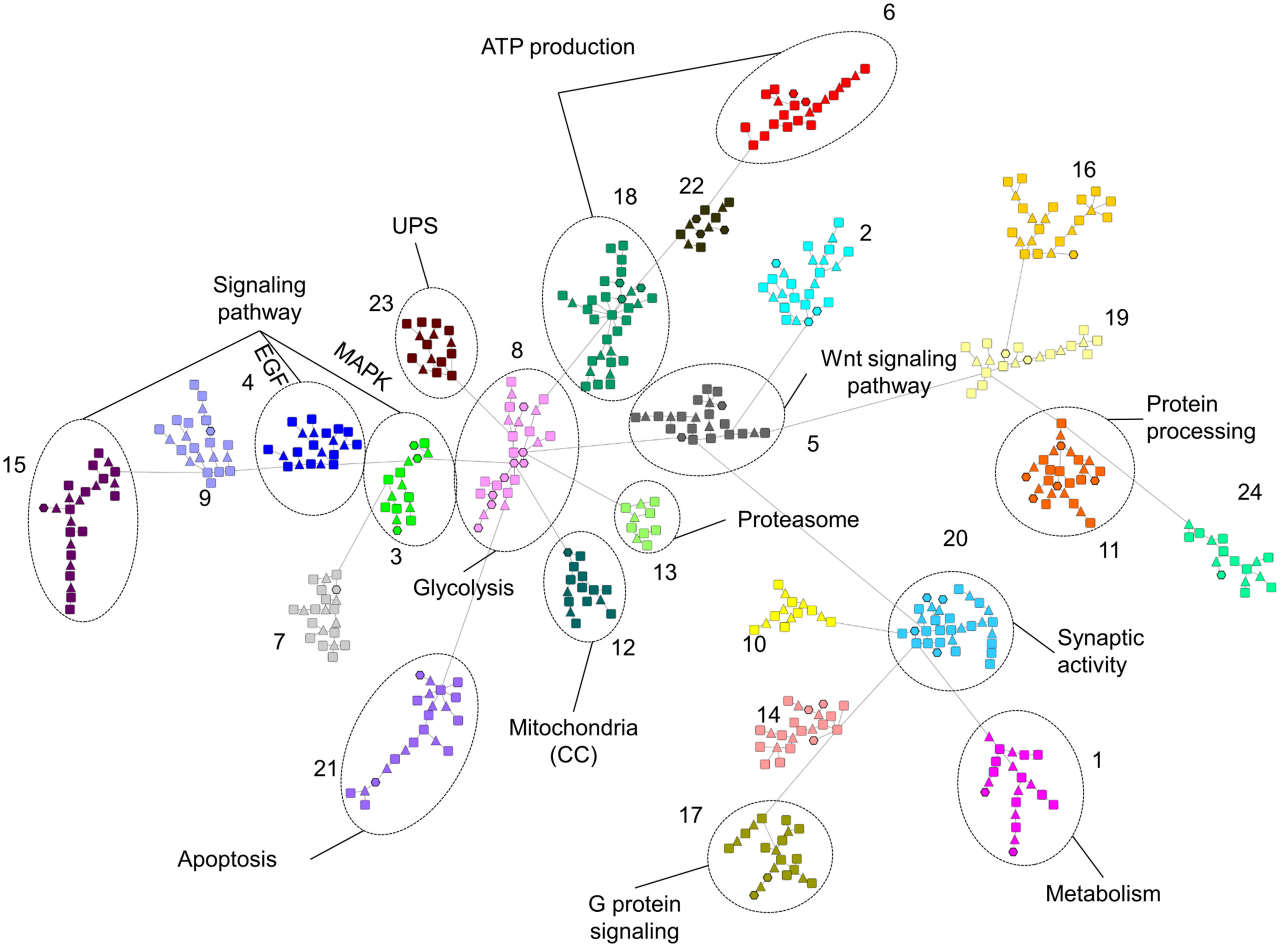
**Subnetwork clustering of the D2 model of the META list, using the community detection algorithms of GLay**. Subnetworks are evidenced by different colors and numbered. Unambiguously over-represented categories are indicated. See Supplementary Tables 9, 10 for details.

Figure 3 shows the same network of Figure 2D, highlighting the clusters found by GLay. Twenty-four clusters have been originated by the original network. Since it was not possible to interpret univocally all subnetworks, we considered only the pathways identified in an unambiguous way by all the analyses and labeled them on Figure 3. Concerning cluster 12, we evidenced the enrichment in mitochondrial proteins, as detected by ORA, using the GO Cellular Component (CC) category. The complete list of ORA analysis results, using GO as a reference set, is detailed in Supplementary Table 9, whereas the outcome of the Webgestalt and Reactome analysis is presented in Supplementary Table 10.

In summary, our analysis revealed the direct effect of MPP^+^ on ATP production by mitochondria; the alteration of VDACs and the consequent dysregulation of the transport to and from the mitochondria; the activation of the mitochondrial unfolded stress response (mt-UPR), together with the pivotal role of HSP60 in directing it; the alteration of the synapse functionality, in particular of the SNARE complex and proteins involved in the endocytosis; the possible signaling cascades activated by the toxin, e.g., EGF, MAPK; the triggering of apoptosis and the autophagy induction.

## Discussion

The MPTP/MPP^+^ model has been widely used to reproduce a Parkinson-like syndrome in animal models as well as mitochondrial impairment in cellular models. Notwithstanding this, very few is known about which specific pathways are altered by the toxin and how MPP^+^ treatment is able to recapitulate PD pathogenesis at the cellular level. The MPTP model is particularly suitable to study PD neurodegeneration for two main reasons: it specifically affects dopaminergic neurons since its active metabolite, MPP^+^, enters via the DAT transporter. Secondly, it acts as a complex I inhibitor and several lines of evidence have demonstrated an involvement of the complex I dysfunction in PD pathogenesis. Complex I deficiency and oxidative damage have been frequently observed in affected neurons and in peripheral tissues of PD patients (Schapira et al., 1989; Benecke et al., 1993; Parker and Swerdlow, 1998; Ambrosi et al., 2014). Moreover, it has been more recently demonstrated that the enzymatic activity of complex I depends on the integrity of the kinase domain of PINK1 (Morais et al., 2009). Consequently, also the pathogenetic mechanisms related to the autosomal recessive PD linked to PINK1 mutations impinges on the impairment of complex I activity (Morais et al., 2014).

In order to exploit the large mole of data present in the literature, we merged the results of several papers containing proteomics experiments on different MPTP-models. With this approach it is possible to overcome the limit of each model or methodology used and to extrapolate the biochemical pathways altered by the complex I blockade. By ORA, we tried to overcome the limits of different databases using several of them and searching for “consensus” pathways. The ORA of the EXP list underlined the involvement of VDACs in altering the transport to and from the mitochondria. The importance of these channels in determining mitochondrial function and consequently the cell fate is the subject of many recent reports (Geisler et al., 2010; Shoshan-Barmatz et al., 2010; Sun et al., 2012; Alberio et al., 2014). The direct damage of ETC by MPP^+^ causes mitochondria depolarization (Nakamura et al., 2000). The electrochemical potential serves not only for ATP production but also for the correct import of proteins into the organelle (Friedman and Nunnari, 2014). Therefore, the transport is generally dysregulated (Burté et al., 2011) and may further damage mitochondrial function. Eventually, loss of import together with the loss of potential leads to the mitochondrial unfolded protein stress response (mt-UPR), i.e., molecular chaperones and proteases that promote proper protein folding, complex assembly and quality control (Haynes and Ron, 2010). Again “protein folding” is a GO term over-represented in our list, and proteins belonging to pathways with a similar meaning of other databases are found to be over-represented, too (e.g., “Regulation of HSF1-mediated heat shock response” in Reactome, “Unfolded Protein Response” in Pathway Commons). Since the EXP list was the result of a mitochondrial proteomics investigation, it is straightforward that the overrepresented pathways were mainly mitochondrial. Even if they were general cellular pathways, they stressed the involvement of these organelles in guiding cellular events. On the one hand, this permitted us to focus on the organelle and its proteome alterations; at the same time, it constituted a limit in the understanding of the MPP^+^ action as a whole. Therefore, the integration of data coming from the literature allowed us to complete the scenario. All the analyses on the META list pointed out the alteration of proteins involved in the synapse functionality. As a consequence of the mitochondrial dysfunction, and of a lower ATP production, it is probable that the synapse structure and function are prejudiced. Indeed, neurons normally rely on oxidative phosphorylation for ATP production rather than on glycolysis (Bolaños et al., 2010). Thus, the energetic needs of the synapse are no more fulfilled upon an impairment of the ETC. This may justify the effect observed at the protein level. Moreover, mitochondria should provide ATP supply and calcium buffering locally, at the high energy-demanding synapses (Schon and Przedborski, 2011). This is the reason for the high number of mitochondria residing in neuronal processes (Amadoro et al., 2014). The lack of these functions at the synapse may be the cause of the impairment of neurotransmitter synthesis and release. Additionally, it must be considered that a direct cause-effect relationship between mitochondrial number and activity with the number and plasticity of spines and synapses has been demonstrated, as the degeneration of synapses as a consequence of a local loss of mitochondria (Li et al., 2004).

The network enrichment is a powerful tool, which may help in fixing the false negative issue. Several molecular actors should contribute to the effects observed, but they may not be detected by the experiments performed for several reasons (e.g., limits of the techniques). Adding nodes to the network may reveal these proteins and the ORA of the enriched networks may highlight previously hidden molecular pathways. As networks generated with Rspider are concerned, the only significant network generated with the EXP list was a four-nodes graph, which stressed the alteration of the ATP production at the mitochondrial level and transport of ATP in the cytosol through carriers (SLC family members). By taking into account several reports on proteomics investigations on cellular and animal models, though, it was possible to obtain two extended networks. The D1 model was particularly interesting because, by simply merging the literature data without adding any node, it evidenced the action of the mitochondrial toxin on the synapse functionality. Two clusters were enriched in proteins involved in the synaptic transmission: members of the SNARE complex and associated regulatory proteins or adaptor proteins for the vesicles endocytosis, responsible for the neurotransmitter re-uptake and release. The D2 model further enlarged the view, integrating those proteins with others involved in the signaling pathways, in the triggering of apoptosis and in ATP production. The proteins of the EXP list were evidenced by a hexagonal shape to show how our experimental findings contributed to the state-of-the-art. Apoptosis induction was frequently observed after MPP^+^ treatment (Liu et al., 2008; Bové and Perier, 2012; Campello et al., 2013; Alberio et al., 2014), but a focus on the proteins involved in the process can reveal which molecular mechanisms are specifically responsible for the MPP^+^-induced dopaminergic neurons death.

The PPI analysis of both lists gave rise to quite complex networks. Apart from the D1 model of the EXP list, which evidenced once again the central role of VDACs, the other networks were too complex to be evaluated by simply considering the edges generated and the enriched categories identified on the network by Bioprofiling. However, networks built using physical interaction information may provide valuable insights when analyzed in terms of over-representation of functional categories. Consequently, we analyzed them by creating a list with all the nodes included in the D2 model of the EXP list and the D1 model of the META list and by searching for over-represented GO categories, while the much larger D2 model of the META list was analyzed after segmentation in subnetworks. The D2 model of the EXP list showed a peculiar topology, since all the network branches originated from a central hub, namely HSPD1 (alias HSP60). In PPI networks hubs may represent higher-order communication points between protein complexes (Han et al., 2005). Indeed, this visual representation of data was able to evidence the pivotal role of HSP60 in directing the mt-UPR. The nuclear encoded chaperone HSP60 is induced by the presence of unfolded proteins inside the organelle (Jovaisaite et al., 2014), and was revealed to be up-regulated after MPP^+^ treatment by our mitochondrial proteomics analysis. Accumulating unfolded proteins, unassisted by chaperone HSP60 in stressed mitochondria, are committed to be digested by proteases (Jovaisaite et al., 2014). In this regard, the ORA analysis of the network statistically validated the importance of the “response to unfolded protein” GO term. Moreover, it highlighted the possible involvement of other pathways possibly altered by MPP^+^, such as the mTOR signaling cascade and the DNA repair pathway.

As far as the D1 model of the META list is concerned, the significant results included once again the over-representation of proteins acting at the synapse and implicated in the transport to mitochondria. Again, the category “autophagic vacuole assembly” linked to autophagy (e.g., the mTOR signaling cascade) appeared to be over-represented. This observation is in agreement with previous reports on the autophagy induction by MPP^+^ and its ability to dysregulate the process, leading to unfolded proteins and impaired mitochondria accumulation, and further ROS production (Dagda et al., 2013). This is particularly important, since neurons depend on autophagy for differentiation and survival (Komatsu et al., 2006).

The simultaneous consideration of several proteome alterations by the meta-analysis allowed us to define which pathways are mainly influenced by MPP^+^. However, it is possible that several proteins playing an important role in some of these processes were not detected by any of the considered papers. The reasons might be various. For example, all the proteomics studies included in the present work dealt with quantitative changes, whereas the contribution of a protein can be determined by other factors, e.g., post-translational modifications or cellular localization. To this purpose, we generated the D2 model of the META list in order to further enrich the scenario. Nevertheless, it was necessary to use a different strategy to analyze this extended network. Using a network clustering algorithm, we were able to obtain subnetworks that may represent highly interconnected proteins in protein complexes or functional modules (Stelzl et al., 2005). The ORA analysis suggested which signaling pathways may be altered by MPP^+^, e.g., the Wnt signaling pathway, already described to be involved in the MPTP-induced loss and repair of nigrostriatal dopaminergic neurons (L'Episcopo et al., 2012).

The bioinformatics analysis presented here may have some shortcomings. First of all, we decided to include only papers based on unbiased proteomics experiments, thus ignoring information coming from other kinds of methodological approaches. This effort was done to complete the interpretation of data of each single paper and to suggest a possible workflow to interpret the long list of proteins normally resulting from proteomics studies. Nevertheless, the neglected information was implicitly taken into account when missing proteins were added by the enrichment procedures. Another shortcoming of PPI graphs is their static nature. On the contrary, interacting proteins are highly dynamic structures, changing in time and space. Eventually, all the false positive or negative data present in the databases are mirrored in the networks built on those information. Being aware of the limits of this approach, we succeeded to describe how the mitochondrial toxin MPP^+^ alters the mitochondrial proteome, overcoming the issues of every single methodology or model. In some cases, the information obtained putting all the results together is more than simply their sum. For example, the dysregulation at the synapse level may be one of the leading event in describing MPP^+^ toxicity. As a whole, this analysis clarified the cellular pathways altered by MPP^+^ and how this toxic model can recapitulate some pathogenetic events of PD. This may serve at one side to guide future research on PD using these models and on the other side to envisage new neuroprotective agents to revert the degenerative process.

## Author contributions

Tiziana Alberio and Mauro Fasano conceived and designed the studies. Chiara Monti and Tiziana Alberio performed the analyses. Tiziana Alberio and Chiara Monti wrote the manuscript. Andrea Urbani and Heather Bondi critically revised the data. All authors contributed to the editing and preparation of the manuscript. All authors approved the final version of the manuscript.

### Conflict of interest statement

The authors declare that the research was conducted in the absence of any commercial or financial relationships that could be construed as a potential conflict of interest.
