# Supplementary material for: Systems biology analysis of the proteomic alterations induced by MPP+, a Parkinson's disease-related mitochondrial toxin
Source: Front Cell Neurosci. 2015 Feb 2;9:14. doi: 10.3389/fncel.2015.00014 (PMC4313704; doi:10.3389/fncel.2015.00014)
Supplement: Supplementary file 1 [file DataSheet1.DOCX]

**SUPPLEMENTARY MATERIAL**

**Systems biology analysis of the proteomic alterations induced by MPP^+^, a Parkinson’s disease-related mitochondrial toxin**

Chiara Monti^1^, Heather Bondi^1,2^, Andrea Urbani^3,4^, Mauro Fasano^1,2^, Tiziana Alberio^*1,2^

^1^Biomedical Research Division, Department of Theoretical and Applied Sciences, University of Insubria, Busto Arsizio, Italy.

^2^Center of Neuroscience, University of Insubria, Busto Arsizio, Italy

^3^Santa Lucia IRCCS Foundation, Rome, Italy

^4^Department of Experimental Medicine and Surgery, University of Rome "Tor Vergata", Rome, Italy.

^*^Dr. TizianaAlberio

University of Insubria

Department of Theoretical and Applied Sciences, Biomedical Research Division

Laboratory of Biochemistry and Functional Proteomics

via Manara 7, I-21052 Busto Arsizio (VA)

E-mail: tiziana.alberio@uninsubria.it; Fax: +39 0332 395599; Tel: +39 0331 339485

**Running title**: MPP^+^ systems biology analysis

**Table 1**: input lists.

| **EXP INPUT LIST** | |
| --- | --- |
| **Alberio et al., 2014** | ACTB, ACTBL2, ANXA2, ANXA3, ATP5A1, ATP5H, C1QBP, CAP1, CFL1, CS, EEF1A1, FUBP1, GAPDH, H2AFV, HIST1H1B, HIST1H2BF, HIST3H3, HIST4H4, HSP90AB1, HSP90B1, HSPA1A, HSPA1L, HSPA5, HSPA6, HSPA9, HSPD1, IMMT, LMNA, LRPPRC, MARCKS, MDH2, NCL, NDUFS3, PDIA3, PHB, PHB2, PKM, PLD3, POTEE, POTEI, PPIA, PRDX3, RPSA, SEPT7, SFXN1, SLC25A13, SLC25A3, SLC25A5, SLC25A6, SRCAP, SSBP1, TUBA1B, TUBA1C, TUFM, UBA52, VDAC1, VDAC2, VDAC3, VIM |

| **META INPUT LIST** | |
| --- | --- |
| **Alberio et al., 2014** | ACTB, ACTBL2, ANXA2, ANXA3, ATP5A1, ATP5H, C1QBP, CAP1, CFL1, CS, EEF1A1, FUBP1, GAPDH, H2AFV, HIST1H1B, HIST1H2BF, HIST3H3, HIST4H4, HSP90AB1, HSP90B1, HSPA1A, HSPA1L, HSPA5, HSPA6, HSPA9, HSPD1, IMMT, LMNA, LRPPRC, MARCKS, MDH2, NCL, NDUFS3, PDIA3, PHB, PHB2, PKM, PLD3, POTEE, POTEI, PPIA, PRDX3, RPSA, SEPT7, SFXN1, SLC25A13, SLC25A3, SLC25A5, SLC25A6, SRCAP, SSBP1, TUBA1B, TUBA1C, TUFM, UBA52, VDAC1, VDAC2, VDAC3, VIM |
| **Burté et al., 2011** | ATP1A2, ATP5H, CCT5, ECHS1, ENO1, ERP29, ETFA, GOT2, HSPA8, HSPD1, IMMT, MDH2, OXCT1, STIP1, VDAC1 |
| **Campello et al., 2013** | ATP5F1, CALB1, COX5A, DDAH1, ENO1, ENO2, GAPDH, HSPA5, HSPA8, MPST, NME2, PPA2, SAG, SNCG, STMN1 |
| **Chin et al., 2008** | ATP5C1, ATP6V1D, ATP6V1F, ATPIF1, BCAS1, BDH1, BIN1, CAMK4, CAPNS1, CAPZB, CASK, CBR1, CCT7, CHMP7, CLTA, CLTB, CLU, COPS2, COX6C, CTSB, CTSD, CYB5B, DBN1, DDAH1, DDC, DGKB, DYNLL1, EEF1D, EHD1, EHD3, ENPP6, ENSA, EXOC7, GAD2, GFAP, GLO1, GNA13, TECR, GRHPR, GRIPAP1, GSPT1, HAPLN1, HDGF, HIST1H2AA, HIST1H2BG, HNRNPH1, HNRNPH2, HPCA, HPCAL1, HPCAL4, ITSN1, KIF5B, KIF5C, KLC3, L1CAM, LAP3, LASP1, LMNB1, LMNB2, MAOB, MAPK1, MINK1, MRPS36, NCKAP1, NCKIPSD, NDUFA13, NDUFA2, NDUFB4, NDUFB5, NDUFB7, NEDD4, NGEF, NME2, NPTN, NPTX1, PACS1, PCBD1, PCYOX1, AIFM1, PEA15, PFKL, PGM2, PHACTR1, PHB, PHB2, PIP4K2B, PPID, PPP2R2D, PPP2R5E, PRAF2, PREP  PSMD1, PTPRD, PTPRS, RAB5A, RHOB, RIMS1, RPL10A, RPS6KA5, RTN1, SCAMP1, SCRN3, SERBP1, SFXN3, SORBS1, SRM, SYP, TAGLN2, TH, THY1, TMSB4X, TOLLIP, TPP1, TPT1, TRAP1, TERF1, TRIM2, TSPAN7, UBE2L3, UBE2N, UBQLN2, UQCRQ, USP9X, VAMP2, VIM |
| **Choi et al., 2014** | AARS2, ASNS, ATP5F1, BTF3, CAP1, CDK6, COX2, DCXR, DDX24, EIF4H, ELFN1, GSTM2, HIST1H2AB, HIST1H2AE, HIST1H2BK, HSD17B10, HSP90AA1, KARS, LAMA4, LCP1, MCTS1, MRPL10, MRPL3, NDUFA9, NDUFS3, NIPSNAP1, NSUN2, NUDT21, PTPLAD1, RPLP1, SCCPDH, SF3A3, SNRNP70, SNRPD1, SPTBN2, TOMM40, UBE2S, VIM |
| **Diedrich et al., 2008** | ACOT2, ACTB, ACTG1, ADCY6, AKR1B1, ALDOA, ALDOC, ARL3, ARPC5, ARPC5L, ATP5B, AUH, CAT, CBR4, CHDH, CHMP5, CKB, CLCF1, CNN3, CPLX1, CPLX2, CRMP1, CSTB, DDAH1, EEF1A1, EEF1A2, EEF1B2, EIF3G, ESD, GABARAPL2, GDA, GLRX3, GOT1, GPI, HADHA, HSPA1A, HSPA4, APG1, INPP5D, LAP3, MDH2, MGLL, MSN, MTPN, NDRG1, NDUFA12, NDUFA4, NDUFA5, NDUFS1, NEFL, NEFM, OXCT1, P4HB, PDHB, PDXK, PEBP1, PFN1, PIK3CA, PRDX5, PSMA5, PSMF1, PVALB, RNH1, SEPT5, SH3GL1, SNAP25, SNCA, SNRPF, STMN1, STXBP1, TKT, TOLLIP, TPPP, TPT1, TUBA1C, TUBA3D, TUBA4A, TUBB2B, TUBB2C, TUBB3, UBQLN1, UBXN6, VDAC1, VDAC2, VIM |
| **Dixit et al., 2013** | ATP5F1, DLST, DRP2, ENO1, ENO2, PRDX3, PRRX2, SEPT5, STMN1, TUBA1A |
| **Jin et al., 2005** | ABAT, ACSL1, ACTB, ACTR1A, ADAM22, ADAM23, ADD1, ADSSL1, AKR1B1, ALDOA, ALDOC, ANK2, ANK3, AP2A1, AP2A2, AP2B1, AP2M1, APP, ARHGEF7, ASAP2, ASRGL1, ATL1, ATP1A2, ATP2A2, ATP2B2, ATP2B3, ATP5B, ATP5C1, BAG6, BAZ1A, BDH1, C2CD2L, CABLES2, CAMK2B, CAMKK2, CAPZB, CBL, CCT2, CCT5, CDK5, CFL1, CHCHD3, CHCHD6, CKB, CLDN11, CLTCL1, CNBP, CNP, CORO1A, COX6B1, CPLX2, CPSF2, CTNNBIP1, CUL3, CUTC, CYC1, DDT, DLGAP5, DLST, DNM1, DPYSL5, DYNLL1, EEF1A1, EPB41L2, FLT3LG, GAB1, GABARAP, GABBR2, GAD2, GAPDH, GDI1, GLO1, GNAI1, GNAI2, GNAO1, GNB1, GNB2, GNB4, GNG12, GNG7, GOT2, GPD2, GPM6A, GSTM1, GSTM3, HAGH, HGS, HK1, HPCAL1, HSPA8, HSPD1, IMPG2, LRP8, LYPLA2, MACF1, MAP1LC3C, MAP2, MBP, MGLL, MIF, MMP17, MTCH2, MYO5A, NAPA, NARS, NCAM1, NCDN, NDRG2, NDRG3, NDST1, NDUFA5, NDUFA8, NDUFAB1, NDUFB9, NDUFS1, OLA1, OSBPL3, PACSIN1, PAICS, PDE1A, PDE1B, PDE1C, PDHA1, PDHB, PGRMC2, PKM, PLP1, PLXND1, PPIA, PPP1CB, PPP1CC, PPP1R1B, PPP2R2A, PPP2R5B, PPP2R5C, PPP2R5D, PRKCD, PVRL1, RASAL1, RBM39, RECQL, RPL18A, RPS12, RPS3A, SCN4A, SIRPA, SLC1A2, SLC1A3, SLC25A4, SLC25A5, SLC32A1, SLC38A1, SLC6A11, SLC6A12, SLC6A2, SLC8A1, STX1B, SYP, SYT1, SYT5, TBX18, TCEA2, TIMM13, TPI1, TRRAP, TUBA1A, TUBB2A, TUBB2B, UBA1, UQCRH, VDAC2, VPS35, YWHAE, YWHAQ, YWHAZ |
| **Liu et al., 2008** | OTUD7B, PSMC5, SCN1A, SNCA |
| **Zhao et al., 2007** | BLVRB, C21orf33, GSTM1, HSPB1, LRRK2, PPIA, PSMA2, UBE2N |
| **Zhang et al., 2010** | ACHE, AIFM1, ALDH1A1, ATP6V1D, ATPIF1, BCAS1, BCR, CAPN2, CAPNS1, CASK, CHAT, COX6C, CSE1L, CTSB, CTSD, DDC, FABP3, HNRNPH2, MAOB, NDUFB5, RAN, SRM, TH, THY1, TPT1, VIM |

**Table 2**: Biological Process Gene Ontology categories significantly over-represented in the EXP and META input lists. Analysis performed using the GO Consortium platform (http://geneontology.org/).

| **EXP INPUT LIST** | | |
| --- | --- | --- |
| **Pathway name** | **Gene** | **P-value** |
| protein folding (GO:0006457) | 12 | 2.48E-11 |
| generation of precursor metabolites and energy (GO:0006091) | 13 | 1.70E-09 |
| single-organism process (GO:0044699) | 52 | 5.55E-08 |
| single-organism transport (GO:0044765) | 26 | 5.68E-08 |
| single-organism localization (GO:1902578) | 26 | 2.03E-07 |
| response to unfolded protein (GO:0006986) | 8 | 2.03E-07 |
| response to topologically incorrect protein (GO:0035966) | 8 | 3.22E-07 |
| establishment of localization (GO:0051234) | 28 | 4.10E-07 |
| cellular metabolic process (GO:0044237) | 43 | 4.32E-07 |
| cellular process (GO:0009987) | 53 | 6.60E-07 |
| energy derivation by oxidation of organic compounds (GO:0015980) | 10 | 7.89E-07 |
| single-organism cellular process (GO:0044763) | 48 | 7.90E-07 |
| transport (GO:0006810) | 27 | 1.30E-06 |
| cellular protein metabolic process (GO:0044267) | 25 | 1.71E-06 |
| biological_process (GO:0008150) | 56 | 1.96E-06 |
| organelle organization (GO:0006996) | 22 | 2.32E-06 |
| establishment of localization in cell (GO:0051649) | 18 | 2.77E-06 |
| purine ribonucleoside triphosphate metabolic process (GO:0009205) | 12 | 5.49E-06 |
| ribonucleoside triphosphate metabolic process (GO:0009199) | 12 | 6.09E-06 |
| purine nucleoside triphosphate metabolic process (GO:0009144) | 12 | 6.09E-06 |
| single-organism intracellular transport (GO:1902582) | 14 | 6.33E-06 |
| regulation of apoptotic process (GO:0042981) | 16 | 6.71E-06 |
| cellular localization (GO:0051641) | 19 | 6.88E-06 |
| regulation of programmed cell death (GO:0043067) | 16 | 7.74E-06 |
| nucleoside triphosphate metabolic process (GO:0009141) | 12 | 8.26E-06 |
| regulation of cell death (GO:0010941) | 16 | 1.33E-05 |
| purine ribonucleoside metabolic process (GO:0046128) | 12 | 1.95E-05 |
| purine nucleoside metabolic process (GO:0042278) | 12 | 2.04E-05 |
| protein metabolic process (GO:0019538) | 26 | 2.14E-05 |
| protein refolding (GO:0042026) | 4 | 2.23E-05 |
| ribonucleoside metabolic process (GO:0009119) | 12 | 2.84E-05 |
| localization (GO:0051179) | 28 | 3.05E-05 |
| purine ribonucleotide metabolic process (GO:0009150) | 12 | 3.14E-05 |
| nucleoside metabolic process (GO:0009116) | 12 | 3.90E-05 |
| ribonucleotide metabolic process (GO:0009259) | 12 | 3.95E-05 |
| nucleotide metabolic process (GO:0009117) | 13 | 4.11E-05 |
| ribose phosphate metabolic process (GO:0019693) | 12 | 4.12E-05 |
| purine nucleotide metabolic process (GO:0006163) | 12 | 4.24E-05 |
| nucleoside phosphate metabolic process (GO:0006753) | 13 | 4.37E-05 |
| organic substance metabolic process (GO:0071704) | 41 | 4.54E-05 |
| glycosyl compound metabolic process (GO:1901657) | 12 | 4.94E-05 |
| metabolic process (GO:0008152) | 43 | 6.08E-05 |
| purine ribonucleoside triphosphate catabolic process (GO:0009207) | 10 | 6.86E-05 |
| ribonucleoside triphosphate catabolic process (GO:0009203) | 10 | 6.86E-05 |
| purine nucleoside triphosphate catabolic process (GO:0009146) | 10 | 7.24E-05 |
| primary metabolic process (GO:0044238) | 40 | 7.38E-05 |
| purine-containing compound metabolic process (GO:0072521) | 12 | 7.41E-05 |
| nucleoside triphosphate catabolic process (GO:0009143) | 10 | 7.63E-05 |
| small molecule metabolic process (GO:0044281) | 21 | 8.11E-05 |
| intracellular transport (GO:0046907) | 14 | 8.21E-05 |
| nucleobase-containing small molecule metabolic process (GO:0055086) | 13 | 8.26E-05 |
| purine ribonucleoside catabolic process (GO:0046130) | 10 | 8.47E-05 |
| purine nucleoside catabolic process (GO:0006152) | 10 | 8.47E-05 |
| ribonucleoside catabolic process (GO:0042454) | 10 | 1.01E-04 |
| purine ribonucleotide catabolic process (GO:0009154) | 10 | 1.08E-04 |
| ribonucleotide catabolic process (GO:0009261) | 10 | 1.09E-04 |
| macromolecular complex subunit organization (GO:0043933) | 17 | 1.12E-04 |
| nucleoside catabolic process (GO:0009164) | 10 | 1.25E-04 |
| purine nucleotide catabolic process (GO:0006195) | 10 | 1.36E-04 |
| glycosyl compound catabolic process (GO:1901658) | 10 | 1.38E-04 |
| establishment of protein localization to organelle (GO:0072594) | 8 | 1.41E-04 |
| purine-containing compound catabolic process (GO:0072523) | 10 | 1.47E-04 |
| cellular macromolecule metabolic process (GO:0044260) | 33 | 1.66E-04 |
| nucleotide catabolic process (GO:0009166) | 10 | 1.75E-04 |
| nucleobase-containing compound catabolic process (GO:0034655) | 12 | 1.90E-04 |
| nucleoside phosphate catabolic process (GO:1901292) | 10 | 1.92E-04 |
| protein localization to organelle (GO:0033365) | 9 | 2.09E-04 |
| single-organism metabolic process (GO:0044710) | 27 | 2.18E-04 |
| organophosphate metabolic process (GO:0019637) | 14 | 2.34E-04 |
| single-organism catabolic process (GO:0044712) | 14 | 2.54E-04 |
| cellular nitrogen compound catabolic process (GO:0044270) | 12 | 3.19E-04 |
| aromatic compound catabolic process (GO:0019439) | 12 | 3.22E-04 |
| heterocycle catabolic process (GO:0046700) | 12 | 3.22E-04 |
| viral process (GO:0016032) | 10 | 3.36E-04 |
| organophosphate catabolic process (GO:0046434) | 10 | 3.36E-04 |
| multi-organism cellular process (GO:0044764) | 10 | 3.56E-04 |
| cellular respiration (GO:0045333) | 6 | 3.89E-04 |
| protein targeting (GO:0006605) | 8 | 4.48E-04 |
| regulation of biological quality (GO:0065008) | 20 | 4.73E-04 |
| organic cyclic compound catabolic process (GO:1901361) | 12 | 4.78E-04 |
| cellular component organization (GO:0016043) | 26 | 5.01E-04 |
| oxidation-reduction process (GO:0055114) | 12 | 5.55E-04 |
| ATP metabolic process (GO:0046034) | 8 | 6.87E-04 |
| phosphate-containing compound metabolic process (GO:0006796) | 18 | 6.95E-04 |
| protein complex subunit organization (GO:0071822) | 13 | 7.29E-04 |
| carbohydrate derivative catabolic process (GO:1901136) | 10 | 7.77E-04 |
| cellular component organization or biogenesis (GO:0071840) | 26 | 8.02E-04 |
| interspecies interaction between organisms (GO:0044419) | 10 | 8.62E-04 |
| symbiosis, encompassing mutualism through parasitism (GO:0044403) | 10 | 8.62E-04 |
| phosphorus metabolic process (GO:0006793) | 18 | 9.03E-04 |
| purine ribonucleoside monophosphate metabolic process (GO:0009167) | 8 | 1.12E-03 |
| purine nucleoside monophosphate metabolic process (GO:0009126) | 8 | 1.14E-03 |
| cellular aromatic compound metabolic process (GO:0006725) | 27 | 1.15E-03 |
| response to stress (GO:0006950) | 21 | 1.16E-03 |
| catabolic process (GO:0009056) | 17 | 1.21E-03 |
| 'de novo' protein folding (GO:0006458) | 4 | 1.26E-03 |
| ribonucleoside monophosphate metabolic process (GO:0009161) | 8 | 1.38E-03 |
| organic substance catabolic process (GO:1901575) | 16 | 1.40E-03 |
| single-organism cellular localization (GO:1902580) | 9 | 1.40E-03 |
| nucleoside monophosphate metabolic process (GO:0009123) | 8 | 1.65E-03 |
| nucleobase-containing compound metabolic process (GO:0006139) | 26 | 1.83E-03 |
| macromolecule metabolic process (GO:0043170) | 33 | 2.03E-03 |
| organic cyclic compound metabolic process (GO:1901360) | 27 | 2.80E-03 |
| negative regulation of cellular process (GO:0048523) | 21 | 2.83E-03 |
| mitochondrial transport (GO:0006839) | 5 | 3.02E-03 |
| activation of immune response (GO:0002253) | 7 | 3.12E-03 |
| negative regulation of biological process (GO:0048519) | 22 | 3.13E-03 |
| heat acclimation (GO:0010286) | 2 | 3.16E-03 |
| cellular heat acclimation (GO:0070370) | 2 | 3.16E-03 |
| adenine transport (GO:0015853) | 2 | 3.16E-03 |
| cellular response to interleukin-4 (GO:0071353) | 3 | 3.40E-03 |
| heterocycle metabolic process (GO:0046483) | 26 | 3.77E-03 |
| biological regulation (GO:0065007) | 41 | 3.93E-03 |
| glucose metabolic process (GO:0006006) | 5 | 4.36E-03 |
| organonitrogen compound catabolic process (GO:1901565) | 10 | 4.40E-03 |
| negative regulation of apoptotic process (GO:0043066) | 9 | 4.85E-03 |
| carbohydrate derivative metabolic process (GO:1901135) | 13 | 4.96E-03 |
| cellular macromolecular complex assembly (GO:0034622) | 8 | 5.03E-03 |
| negative regulation of programmed cell death (GO:0043069) | 9 | 5.39E-03 |
| response to interleukin-4 (GO:0070670) | 3 | 6.08E-03 |
| platelet degranulation (GO:0002576) | 4 | 7.18E-03 |
| transmembrane transport (GO:0055085) | 11 | 7.93E-03 |
| negative regulation of cell death (GO:0060548) | 9 | 8.31E-03 |
| cellular nitrogen compound metabolic process (GO:0034641) | 26 | 8.72E-03 |
| purine nucleobase transport (GO:0006863) | 2 | 8.75E-03 |
| regulation of mitochondrial translation (GO:0070129) | 2 | 8.75E-03 |
| regulation of mitochondrion organization (GO:0010821) | 4 | 9.38E-03 |
| multi-organism process (GO:0051704) | 15 | 9.44E-03 |
| ATP catabolic process (GO:0006200) | 6 | 9.52E-03 |
| **META INPUT LIST** | | |
| **Pathway name** | **Gene** | **P-value** |
| single-organism process (GO:0044699) | 423 | 3.57E-60 |
| biological_process (GO:0008150) | 466 | 2.16E-58 |
| single-organism cellular process (GO:0044763) | 394 | 2.86E-55 |
| cellular process (GO:0009987) | 435 | 9.60E-55 |
| small molecule metabolic process (GO:0044281) | 187 | 5.94E-51 |
| generation of precursor metabolites and energy (GO:0006091) | 67 | 4.16E-36 |
| single-organism metabolic process (GO:0044710) | 212 | 3.18E-32 |
| cellular metabolic process (GO:0044237) | 306 | 3.48E-30 |
| transport (GO:0006810) | 180 | 6.20E-30 |
| establishment of localization (GO:0051234) | 182 | 9.38E-30 |
| energy derivation by oxidation of organic compounds (GO:0015980) | 55 | 1.95E-29 |
| single-organism transport (GO:0044765) | 157 | 4.61E-29 |
| single-organism localization (GO:1902578) | 162 | 7.00E-29 |
| catabolic process (GO:0009056) | 133 | 3.62E-28 |
| organonitrogen compound metabolic process (GO:1901564) | 121 | 9.56E-28 |
| single-organism catabolic process (GO:0044712) | 99 | 1.05E-26 |
| organic substance catabolic process (GO:1901575) | 123 | 1.32E-26 |
| localization (GO:0051179) | 196 | 6.87E-26 |
| cellular catabolic process (GO:0044248) | 117 | 7.06E-26 |
| phosphorus metabolic process (GO:0006793) | 134 | 3.91E-25 |
| organophosphate metabolic process (GO:0019637) | 96 | 5.17E-25 |
| cellular component organization (GO:0016043) | 194 | 8.50E-25 |
| establishment of localization in cell (GO:0051649) | 107 | 8.52E-25 |
| metabolic process (GO:0008152) | 322 | 1.04E-24 |
| nucleobase-containing small molecule metabolic process (GO:0055086) | 83 | 4.06E-24 |
| oxidation-reduction process (GO:0055114) | 83 | 4.98E-24 |
| cellular component organization or biogenesis (GO:0071840) | 195 | 6.84E-24 |
| nucleotide metabolic process (GO:0009117) | 80 | 7.94E-24 |
| nucleoside phosphate metabolic process (GO:0006753) | 80 | 1.13E-23 |
| purine ribonucleoside triphosphate metabolic process (GO:0009205) | 66 | 2.30E-23 |
| cellular localization (GO:0051641) | 115 | 3.24E-23 |
| ribonucleoside triphosphate metabolic process (GO:0009199) | 66 | 3.87E-23 |
| purine nucleoside triphosphate metabolic process (GO:0009144) | 66 | 3.87E-23 |
| purine nucleotide metabolic process (GO:0006163) | 72 | 4.41E-23 |
| phosphate-containing compound metabolic process (GO:0006796) | 128 | 1.09E-22 |
| nucleoside triphosphate metabolic process (GO:0009141) | 66 | 1.79E-22 |
| purine ribonucleotide metabolic process (GO:0009150) | 70 | 2.29E-22 |
| ribonucleotide metabolic process (GO:0009259) | 70 | 7.74E-22 |
| purine-containing compound metabolic process (GO:0072521) | 72 | 9.40E-22 |
| ribose phosphate metabolic process (GO:0019693) | 70 | 9.69E-22 |
| nervous system development (GO:0007399) | 114 | 1.03E-21 |
| cellular respiration (GO:0045333) | 35 | 4.12E-21 |
| glycosyl compound metabolic process (GO:1901657) | 69 | 1.23E-20 |
| purine ribonucleoside metabolic process (GO:0046128) | 66 | 1.30E-20 |
| purine nucleoside metabolic process (GO:0042278) | 66 | 1.64E-20 |
| synaptic transmission (GO:0007268) | 60 | 1.96E-20 |
| regulation of biological quality (GO:0065008) | 136 | 5.65E-20 |
| ribonucleoside metabolic process (GO:0009119) | 66 | 8.28E-20 |
| nucleoside metabolic process (GO:0009116) | 66 | 3.95E-19 |
| respiratory electron transport chain (GO:0022904) | 29 | 4.38E-19 |
| organonitrogen compound catabolic process (GO:1901565) | 69 | 5.00E-19 |
| electron transport chain (GO:0022900) | 29 | 7.03E-19 |
| response to stimulus (GO:0050896) | 250 | 9.24E-19 |
| organic substance metabolic process (GO:0071704) | 287 | 4.10E-18 |
| vesicle-mediated transport (GO:0016192) | 72 | 4.66E-17 |
| primary metabolic process (GO:0044238) | 276 | 4.02E-16 |
| intracellular transport (GO:0046907) | 77 | 4.06E-16 |
| response to stress (GO:0006950) | 140 | 4.32E-16 |
| response to organic substance (GO:0010033) | 113 | 4.99E-16 |
| anatomical structure development (GO:0048856) | 168 | 5.53E-16 |
| heterocycle catabolic process (GO:0046700) | 68 | 7.37E-16 |
| biological regulation (GO:0065007) | 310 | 1.17E-15 |
| single-multicellular organism process (GO:0044707) | 207 | 1.18E-15 |
| organic cyclic compound catabolic process (GO:1901361) | 69 | 1.36E-15 |
| response to chemical (GO:0042221) | 149 | 2.63E-15 |
| cellular nitrogen compound catabolic process (GO:0044270) | 67 | 2.72E-15 |
| neurogenesis (GO:0022008) | 80 | 6.09E-15 |
| organophosphate catabolic process (GO:0046434) | 53 | 1.05E-14 |
| nucleotide catabolic process (GO:0009166) | 51 | 1.11E-14 |
| system development (GO:0048731) | 149 | 1.35E-14 |
| developmental process (GO:0032502) | 180 | 1.46E-14 |
| purine nucleotide catabolic process (GO:0006195) | 50 | 1.65E-14 |
| nucleoside phosphate catabolic process (GO:1901292) | 51 | 1.69E-14 |
| carbohydrate derivative metabolic process (GO:1901135) | 84 | 1.83E-14 |
| purine-containing compound catabolic process (GO:0072523) | 50 | 2.36E-14 |
| single-organism developmental process (GO:0044767) | 178 | 2.79E-14 |
| generation of neurons (GO:0048699) | 76 | 3.03E-14 |
| aromatic compound catabolic process (GO:0019439) | 65 | 4.13E-14 |
| single-organism intracellular transport (GO:1902582) | 65 | 4.35E-14 |
| multicellular organismal process (GO:0032501) | 208 | 5.72E-14 |
| organic substance transport (GO:0071702) | 93 | 8.80E-14 |
| cell-cell signaling (GO:0007267) | 64 | 1.07E-13 |
| purine ribonucleotide catabolic process (GO:0009154) | 48 | 1.32E-13 |
| ribonucleotide catabolic process (GO:0009261) | 48 | 1.42E-13 |
| nucleobase-containing compound catabolic process (GO:0034655) | 62 | 1.93E-13 |
| regulation of neurotransmitter levels (GO:0001505) | 23 | 3.41E-13 |
| carboxylic acid metabolic process (GO:0019752) | 61 | 3.46E-13 |
| purine ribonucleoside triphosphate catabolic process (GO:0009207) | 46 | 4.48E-13 |
| ribonucleoside triphosphate catabolic process (GO:0009203) | 46 | 4.48E-13 |
| purine nucleoside triphosphate catabolic process (GO:0009146) | 46 | 5.55E-13 |
| nucleoside triphosphate catabolic process (GO:0009143) | 46 | 6.85E-13 |
| purine ribonucleoside catabolic process (GO:0046130) | 46 | 1.04E-12 |
| purine nucleoside catabolic process (GO:0006152) | 46 | 1.04E-12 |
| protein complex subunit organization (GO:0071822) | 72 | 1.27E-12 |
| ribonucleoside catabolic process (GO:0042454) | 46 | 2.06E-12 |
| single organism signaling (GO:0044700) | 177 | 3.44E-12 |
| signaling (GO:0023052) | 177 | 3.44E-12 |
| cell communication (GO:0007154) | 179 | 3.98E-12 |
| subthalamus development (GO:0021539) | 16 | 4.68E-12 |
| nucleoside catabolic process (GO:0009164) | 46 | 4.89E-12 |
| neuron development (GO:0048666) | 54 | 7.19E-12 |
| glycosyl compound catabolic process (GO:1901658) | 46 | 7.22E-12 |
| organic acid metabolic process (GO:0006082) | 63 | 1.33E-11 |
| multicellular organismal development (GO:0007275) | 157 | 1.37E-11 |
| neuron differentiation (GO:0030182) | 60 | 1.42E-11 |
| neurotransmitter transport (GO:0006836) | 22 | 1.53E-11 |
| oxoacid metabolic process (GO:0043436) | 62 | 2.07E-11 |
| purine ribonucleoside monophosphate metabolic process (GO:0009167) | 39 | 2.09E-11 |
| purine nucleoside monophosphate metabolic process (GO:0009126) | 39 | 2.25E-11 |
| neuron projection development (GO:0031175) | 48 | 2.75E-11 |
| carbohydrate derivative catabolic process (GO:1901136) | 50 | 3.28E-11 |
| intracellular protein transport (GO:0006886) | 47 | 3.39E-11 |
| establishment of protein localization (GO:0045184) | 68 | 3.73E-11 |
| cellular component assembly (GO:0022607) | 81 | 3.98E-11 |
| substantia nigra development (GO:0021762) | 15 | 4.24E-11 |
| hemostasis (GO:0007599) | 42 | 4.38E-11 |
| regulation of localization (GO:0032879) | 89 | 4.49E-11 |
| macromolecular complex subunit organization (GO:0043933) | 88 | 4.66E-11 |
| ribonucleoside monophosphate metabolic process (GO:0009161) | 39 | 5.07E-11 |
| ATP metabolic process (GO:0046034) | 37 | 6.29E-11 |
| protein transport (GO:0015031) | 65 | 9.05E-11 |
| organelle organization (GO:0006996) | 105 | 9.63E-11 |
| ion transport (GO:0006811) | 68 | 1.05E-10 |
| nucleoside monophosphate metabolic process (GO:0009123) | 39 | 1.11E-10 |
| GTP metabolic process (GO:0046039) | 29 | 1.16E-10 |
| coagulation (GO:0050817) | 41 | 1.37E-10 |
| blood coagulation (GO:0007596) | 41 | 1.37E-10 |
| regulation of cellular component organization (GO:0051128) | 81 | 1.44E-10 |
| nitrogen compound metabolic process (GO:0006807) | 187 | 1.56E-10 |
| GTP catabolic process (GO:0006184) | 28 | 2.08E-10 |
| protein folding (GO:0006457) | 27 | 2.14E-10 |
| cellular nitrogen compound metabolic process (GO:0034641) | 176 | 2.44E-10 |
| membrane organization (GO:0061024) | 49 | 2.50E-10 |
| guanosine-containing compound catabolic process (GO:1901069) | 28 | 2.84E-10 |
| guanosine-containing compound metabolic process (GO:1901068) | 29 | 3.48E-10 |
| endocytosis (GO:0006897) | 37 | 3.70E-10 |
| cell differentiation (GO:0030154) | 117 | 5.37E-10 |
| response to wounding (GO:0009611) | 48 | 6.69E-10 |
| cellular component biogenesis (GO:0044085) | 83 | 6.74E-10 |
| cellular developmental process (GO:0048869) | 120 | 7.88E-10 |
| cellular protein localization (GO:0034613) | 57 | 1.03E-09 |
| regulation of biological process (GO:0050789) | 282 | 1.08E-09 |
| single-organism biosynthetic process (GO:0044711) | 65 | 1.21E-09 |
| cellular macromolecule localization (GO:0070727) | 57 | 1.27E-09 |
| regulation of transport (GO:0051049) | 71 | 1.52E-09 |
| cell projection organization (GO:0030030) | 55 | 1.52E-09 |
| glucose metabolic process (GO:0006006) | 22 | 2.16E-09 |
| cell death (GO:0008219) | 68 | 2.23E-09 |
| protein localization (GO:0008104) | 75 | 2.35E-09 |
| wound healing (GO:0042060) | 44 | 2.48E-09 |
| death (GO:0016265) | 68 | 2.56E-09 |
| regulation of body fluid levels (GO:0050878) | 44 | 2.62E-09 |
| mitochondrial ATP synthesis coupled electron transport (GO:0042775) | 15 | 3.09E-09 |
| neuron projection morphogenesis (GO:0048812) | 40 | 3.25E-09 |
| cellular response to chemical stimulus (GO:0070887) | 91 | 3.37E-09 |
| oxidative phosphorylation (GO:0006119) | 16 | 3.85E-09 |
| ATP synthesis coupled electron transport (GO:0042773) | 15 | 3.94E-09 |
| cellular response to stimulus (GO:0051716) | 185 | 4.82E-09 |
| regulation of cellular process (GO:0050794) | 269 | 5.39E-09 |
| cellular response to organic substance (GO:0071310) | 78 | 5.51E-09 |
| cell morphogenesis involved in neuron differentiation (GO:0048667) | 39 | 5.67E-09 |
| neural nucleus development (GO:0048857) | 15 | 9.91E-09 |
| macromolecule localization (GO:0033036) | 82 | 9.99E-09 |
| neurotransmitter secretion (GO:0007269) | 16 | 1.07E-08 |
| regulation of signaling (GO:0023051) | 106 | 1.16E-08 |
| regulation of cell communication (GO:0010646) | 106 | 1.37E-08 |
| cellular aromatic compound metabolic process (GO:0006725) | 164 | 2.18E-08 |
| regulation of molecular function (GO:0065009) | 101 | 2.49E-08 |
| regulation of programmed cell death (GO:0043067) | 66 | 2.50E-08 |
| hexose metabolic process (GO:0019318) | 23 | 2.66E-08 |
| regulation of catalytic activity (GO:0050790) | 89 | 3.84E-08 |
| axonogenesis (GO:0007409) | 36 | 4.77E-08 |
| cellular protein metabolic process (GO:0044267) | 116 | 5.09E-08 |
| cell part morphogenesis (GO:0032990) | 44 | 5.35E-08 |
| monocarboxylic acid metabolic process (GO:0032787) | 36 | 5.68E-08 |
| cell projection morphogenesis (GO:0048858) | 43 | 8.92E-08 |
| protein metabolic process (GO:0019538) | 133 | 9.50E-08 |
| interspecies interaction between organisms (GO:0044419) | 44 | 1.02E-07 |
| symbiosis, encompassing mutualism through parasitism (GO:0044403) | 44 | 1.02E-07 |
| regulation of apoptotic process (GO:0042981) | 64 | 1.18E-07 |
| organic cyclic compound metabolic process (GO:1901360) | 167 | 1.23E-07 |
| negative regulation of biological process (GO:0048519) | 130 | 1.28E-07 |
| regulation of cell death (GO:0010941) | 66 | 1.29E-07 |
| axon development (GO:0061564) | 36 | 1.33E-07 |
| negative regulation of cellular process (GO:0048523) | 122 | 1.38E-07 |
| cell development (GO:0048468) | 68 | 1.42E-07 |
| viral process (GO:0016032) | 41 | 1.54E-07 |
| multi-organism cellular process (GO:0044764) | 41 | 1.87E-07 |
| energy reserve metabolic process (GO:0006112) | 20 | 1.94E-07 |
| 'de novo' protein folding (GO:0006458) | 13 | 1.94E-07 |
| macromolecular complex assembly (GO:0065003) | 56 | 2.45E-07 |
| mitochondrial electron transport, NADH to ubiquinone (GO:0006120) | 12 | 2.92E-07 |
| central nervous system development (GO:0007417) | 47 | 3.06E-07 |
| synaptic vesicle transport (GO:0048489) | 14 | 3.11E-07 |
| establishment of synaptic vesicle localization (GO:0097480) | 14 | 3.11E-07 |
| cell morphogenesis (GO:0000902) | 48 | 3.36E-07 |
| positive regulation of biological process (GO:0048518) | 142 | 3.38E-07 |
| synaptic vesicle localization (GO:0097479) | 14 | 3.75E-07 |
| monosaccharide biosynthetic process (GO:0046364) | 13 | 3.75E-07 |
| cellular component movement (GO:0006928) | 62 | 4.18E-07 |
| glycolytic process (GO:0006096) | 12 | 4.79E-07 |
| gluconeogenesis (GO:0006094) | 12 | 4.79E-07 |
| protein targeting (GO:0006605) | 30 | 5.12E-07 |
| heterocycle metabolic process (GO:0046483) | 159 | 5.25E-07 |
| cell morphogenesis involved in differentiation (GO:0000904) | 40 | 5.71E-07 |
| monosaccharide metabolic process (GO:0005996) | 23 | 6.07E-07 |
| secretion by cell (GO:0032940) | 32 | 6.13E-07 |
| 'de novo' posttranslational protein folding (GO:0051084) | 12 | 7.67E-07 |
| hexose biosynthetic process (GO:0019319) | 12 | 1.21E-06 |
| signal release (GO:0023061) | 18 | 1.24E-06 |
| cellular component morphogenesis (GO:0032989) | 49 | 1.41E-06 |
| platelet activation (GO:0030168) | 22 | 1.48E-06 |
| response to topologically incorrect protein (GO:0035966) | 18 | 1.95E-06 |
| regulation of synaptic transmission (GO:0050804) | 22 | 2.07E-06 |
| axon guidance (GO:0007411) | 29 | 2.22E-06 |
| neuron projection guidance (GO:0097485) | 29 | 2.22E-06 |
| secretion (GO:0046903) | 35 | 2.30E-06 |
| immune system process (GO:0002376) | 80 | 2.43E-06 |
| single-organism cellular localization (GO:1902580) | 37 | 2.50E-06 |
| protein complex assembly (GO:0006461) | 48 | 2.68E-06 |
| protein complex biogenesis (GO:0070271) | 48 | 3.10E-06 |
| response to external stimulus (GO:0009605) | 73 | 3.30E-06 |
| single-organism organelle organization (GO:1902589) | 69 | 3.60E-06 |
| cellular response to growth factor stimulus (GO:0071363) | 36 | 3.78E-06 |
| regulation of cellular localization (GO:0060341) | 47 | 4.26E-06 |
| diencephalon development (GO:0021536) | 16 | 4.94E-06 |
| response to unfolded protein (GO:0006986) | 17 | 5.22E-06 |
| transmembrane transport (GO:0055085) | 54 | 5.28E-06 |
| synapse organization (GO:0050808) | 16 | 5.57E-06 |
| exocytosis (GO:0006887) | 23 | 7.04E-06 |
| anatomical structure morphogenesis (GO:0009653) | 80 | 7.88E-06 |
| nucleobase-containing compound metabolic process (GO:0006139) | 150 | 8.00E-06 |
| response to growth factor (GO:0070848) | 36 | 8.19E-06 |
| pyruvate metabolic process (GO:0006090) | 13 | 8.48E-06 |
| response to endogenous stimulus (GO:0009719) | 59 | 8.56E-06 |
| epidermal growth factor receptor signaling pathway (GO:0007173) | 20 | 8.73E-06 |
| cellular amino acid metabolic process (GO:0006520) | 30 | 9.24E-06 |
| establishment of vesicle localization (GO:0051650) | 17 | 9.94E-06 |
| ERBB signaling pathway (GO:0038127) | 20 | 1.03E-05 |
| hydrogen ion transmembrane transport (GO:1902600) | 15 | 1.16E-05 |
| locomotion (GO:0040011) | 53 | 1.28E-05 |
| protein localization to organelle (GO:0033365) | 31 | 1.28E-05 |
| mitochondrion organization (GO:0007005) | 22 | 1.46E-05 |
| organelle localization (GO:0051640) | 23 | 1.76E-05 |
| positive regulation of molecular function (GO:0044093) | 67 | 1.77E-05 |
| forebrain development (GO:0030900) | 27 | 1.79E-05 |
| proton transport (GO:0015992) | 16 | 1.94E-05 |
| establishment of organelle localization (GO:0051656) | 20 | 1.99E-05 |
| vesicle localization (GO:0051648) | 17 | 2.23E-05 |
| negative regulation of epidermal growth factor receptor signaling pathway (GO:0042059) | 10 | 2.38E-05 |
| hydrogen transport (GO:0006818) | 16 | 2.40E-05 |
| response to organonitrogen compound (GO:0010243) | 39 | 2.86E-05 |
| negative regulation of ERBB signaling pathway (GO:1901185) | 10 | 2.98E-05 |
| carbohydrate biosynthetic process (GO:0016051) | 16 | 3.28E-05 |
| cytoskeleton organization (GO:0007010) | 40 | 3.30E-05 |
| single-organism carbohydrate catabolic process (GO:0044724) | 15 | 3.35E-05 |
| brain development (GO:0007420) | 36 | 3.48E-05 |
| mitochondrial transport (GO:0006839) | 16 | 5.42E-05 |
| cytoplasmic transport (GO:0016482) | 36 | 5.85E-05 |
| cell activation (GO:0001775) | 35 | 7.01E-05 |
| carbohydrate catabolic process (GO:0016052) | 15 | 7.16E-05 |
| locomotory behavior (GO:0007626) | 18 | 7.81E-05 |
| cellular macromolecular complex assembly (GO:0034622) | 32 | 7.97E-05 |
| establishment of protein localization to organelle (GO:0072594) | 24 | 8.69E-05 |
| regulation of synaptic plasticity (GO:0048167) | 14 | 8.99E-05 |
| programmed cell death (GO:0012501) | 50 | 9.48E-05 |
| purine ribonucleoside triphosphate biosynthetic process (GO:0009206) | 10 | 1.01E-04 |
| single-organism behavior (GO:0044708) | 25 | 1.05E-04 |
| actin filament-based process (GO:0030029) | 27 | 1.05E-04 |
| purine nucleoside triphosphate biosynthetic process (GO:0009145) | 10 | 1.22E-04 |
| cation transport (GO:0006812) | 41 | 1.22E-04 |
| phosphorylation (GO:0016310) | 49 | 1.27E-04 |
| neurotrophin TRK receptor signaling pathway (GO:0048011) | 22 | 1.42E-04 |
| apoptotic process (GO:0006915) | 49 | 1.42E-04 |
| positive regulation of cellular component organization (GO:0051130) | 37 | 1.45E-04 |
| ATP biosynthetic process (GO:0006754) | 9 | 1.49E-04 |
| purine ribonucleoside monophosphate biosynthetic process (GO:0009168) | 11 | 1.60E-04 |
| purine nucleoside monophosphate biosynthetic process (GO:0009127) | 11 | 1.60E-04 |
| actin cytoskeleton organization (GO:0030036) | 25 | 1.69E-04 |
| neurotrophin signaling pathway (GO:0038179) | 22 | 1.70E-04 |
| antigen processing and presentation of exogenous peptide antigen (GO:0002478) | 17 | 2.05E-04 |
| positive regulation of cellular process (GO:0048522) | 121 | 2.17E-04 |
| regulation of protein polymerization (GO:0032271) | 14 | 2.36E-04 |
| response to nitrogen compound (GO:1901698) | 39 | 2.37E-04 |
| antigen processing and presentation of exogenous antigen (GO:0019884) | 17 | 2.41E-04 |
| multi-organism process (GO:0051704) | 76 | 2.49E-04 |
| signal transduction (GO:0007165) | 139 | 2.70E-04 |
| regulation of epidermal growth factor receptor signaling pathway (GO:0042058) | 11 | 2.90E-04 |
| ribonucleoside triphosphate biosynthetic process (GO:0009201) | 10 | 2.93E-04 |
| nitrogen compound transport (GO:0071705) | 28 | 4.16E-04 |
| amino acid transport (GO:0006865) | 14 | 4.29E-04 |
| regulation of ERBB signaling pathway (GO:1901184) | 11 | 4.40E-04 |
| dicarboxylic acid transport (GO:0006835) | 10 | 4.75E-04 |
| protein tetramerization (GO:0051262) | 12 | 5.13E-04 |
| single-organism membrane organization (GO:0044802) | 31 | 5.98E-04 |
| regulation of response to stimulus (GO:0048583) | 102 | 5.99E-04 |
| positive regulation of catalytic activity (GO:0043085) | 56 | 6.04E-04 |
| immune response-regulating cell surface receptor signaling pathway involved in phagocytosis (GO:0002433) | 11 | 6.55E-04 |
| Fc-gamma receptor signaling pathway involved in phagocytosis (GO:0038096) | 11 | 6.55E-04 |
| negative regulation of signaling (GO:0023057) | 43 | 6.65E-04 |
| antigen processing and presentation of peptide antigen (GO:0048002) | 17 | 6.94E-04 |
| negative regulation of cell communication (GO:0010648) | 43 | 7.05E-04 |
| Fc receptor mediated stimulatory signaling pathway (GO:0002431) | 11 | 7.44E-04 |
| Fc-gamma receptor signaling pathway (GO:0038094) | 11 | 7.44E-04 |
| cellular response to stress (GO:0033554) | 55 | 7.69E-04 |
| ribonucleoside monophosphate biosynthetic process (GO:0009156) | 11 | 8.45E-04 |
| regulation of cell projection organization (GO:0031344) | 23 | 9.88E-04 |
| negative regulation of protein polymerization (GO:0032272) | 9 | 1.19E-03 |
| behavior (GO:0007610) | 28 | 1.24E-03 |
| establishment of protein localization to membrane (GO:0090150) | 18 | 1.29E-03 |
| cellular amino acid catabolic process (GO:0009063) | 13 | 1.39E-03 |
| response to toxic substance (GO:0009636) | 14 | 1.39E-03 |
| regulation of protein complex assembly (GO:0043254) | 18 | 1.47E-03 |
| nucleoside triphosphate biosynthetic process (GO:0009142) | 10 | 1.52E-03 |
| adult behavior (GO:0030534) | 14 | 1.52E-03 |
| negative regulation of protein complex assembly (GO:0031333) | 11 | 1.74E-03 |
| translation (GO:0006412) | 23 | 1.77E-03 |
| regulation of signal transduction (GO:0009966) | 82 | 1.90E-03 |
| platelet degranulation (GO:0002576) | 11 | 1.95E-03 |
| nucleoside monophosphate biosynthetic process (GO:0009124) | 11 | 1.95E-03 |
| intracellular signal transduction (GO:0035556) | 60 | 2.00E-03 |
| homeostatic process (GO:0042592) | 48 | 2.27E-03 |
| synaptic vesicle exocytosis (GO:0016079) | 7 | 2.40E-03 |
| purine ribonucleotide biosynthetic process (GO:0009152) | 13 | 2.40E-03 |
| glutamate secretion (GO:0014047) | 6 | 2.41E-03 |
| translational elongation (GO:0006414) | 12 | 2.81E-03 |
| small molecule catabolic process (GO:0044282) | 19 | 2.94E-03 |
| cellular response to endogenous stimulus (GO:0071495) | 40 | 3.02E-03 |
| cofactor metabolic process (GO:0051186) | 19 | 3.10E-03 |
| protein refolding (GO:0042026) | 6 | 3.28E-03 |
| chemotaxis (GO:0006935) | 31 | 3.30E-03 |
| taxis (GO:0042330) | 31 | 3.30E-03 |
| negative regulation of cellular component organization (GO:0051129) | 26 | 3.35E-03 |
| purine nucleotide biosynthetic process (GO:0006164) | 13 | 3.69E-03 |
| carboxylic acid transport (GO:0046942) | 17 | 3.87E-03 |
| regulation of transferase activity (GO:0051338) | 39 | 4.03E-03 |
| cellular aldehyde metabolic process (GO:0006081) | 8 | 4.12E-03 |
| protein localization to membrane (GO:0072657) | 20 | 4.18E-03 |
| organic acid transport (GO:0015849) | 17 | 4.37E-03 |
| positive regulation of transferase activity (GO:0051347) | 30 | 4.65E-03 |
| purine ribonucleoside biosynthetic process (GO:0046129) | 11 | 5.10E-03 |
| purine nucleoside biosynthetic process (GO:0042451) | 11 | 5.10E-03 |
| response to alkaloid (GO:0043279) | 12 | 5.36E-03 |
| neurotransmitter uptake (GO:0001504) | 5 | 5.59E-03 |
| regulation of organelle organization (GO:0033043) | 34 | 5.62E-03 |
| activation of immune response (GO:0002253) | 22 | 6.09E-03 |
| transmembrane receptor protein tyrosine kinase signaling pathway (GO:0007169) | 30 | 6.51E-03 |
| mitotic cell cycle process (GO:1903047) | 33 | 6.82E-03 |
| regulation of vesicle-mediated transport (GO:0060627) | 19 | 6.86E-03 |
| organonitrogen compound biosynthetic process (GO:1901566) | 30 | 6.96E-03 |
| antigen processing and presentation (GO:0019882) | 17 | 6.98E-03 |
| negative regulation of signal transduction (GO:0009968) | 39 | 7.09E-03 |
| immune response-activating signal transduction (GO:0002757) | 20 | 7.19E-03 |
| positive regulation of transport (GO:0051050) | 31 | 7.28E-03 |
| ribonucleotide biosynthetic process (GO:0009260) | 13 | 7.62E-03 |
| protein localization to mitochondrion (GO:0070585) | 9 | 7.75E-03 |
| chaperone-mediated protein complex assembly (GO:0051131) | 5 | 8.19E-03 |
| macromolecular complex disassembly (GO:0032984) | 13 | 8.22E-03 |
| mitotic cell cycle phase transition (GO:0044772) | 20 | 8.70E-03 |
| ribose phosphate biosynthetic process (GO:0046390) | 13 | 8.87E-03 |
| negative regulation of programmed cell death (GO:0043069) | 33 | 8.93E-03 |
| positive regulation of protein modification process (GO:0031401) | 38 | 8.95E-03 |
| cellular protein complex assembly (GO:0043623) | 18 | 9.17E-03 |
| cellular homeostasis (GO:0019725) | 29 | 9.49E-03 |
| synaptic vesicle endocytosis (GO:0048488) | 6 | 9.59E-03 |
| synaptic vesicle recycling (GO:0036465) | 6 | 9.59E-03 |

**Table 3**: Molecular Function Gene Ontology categories significantly over-represented in the EXP and META input lists. Analysis performed using the GO Consortium platform (http://geneontology.org/).

| **EXP INPUT LIST** | | | | |
| --- | --- | --- | --- | --- |
| **Pathway name** | **Gene** | | | **P-value** |
| RNA binding (GO:0003723) | 25 | | | 2.76E-13 |
| poly(A) RNA binding (GO:0044822) | 22 | | | 8.39E-13 |
| protein binding (GO:0005515) | 47 | | | 8.07E-11 |
| unfolded protein binding (GO:0051082) | 9 | | | 1.85E-10 |
| heterocyclic compound binding (GO:1901363) | 39 | | | 4.88E-10 |
| organic cyclic compound binding (GO:0097159) | 39 | | | 7.31E-10 |
| binding (GO:0005488) | 53 | | | 1.15E-08 |
| nucleic acid binding (GO:0003676) | 30 | | | 4.91E-08 |
| molecular_function (GO:0003674) | 56 | | | 2.04E-07 |
| nucleotide binding (GO:0000166) | 22 | | | 7.75E-07 |
| nucleoside phosphate binding (GO:1901265) | 22 | | | 7.81E-07 |
| small molecule binding (GO:0036094) | 22 | | | 6.04E-06 |
| carbohydrate derivative binding (GO:0097367) | 20 | | | 8.23E-06 |
| purine ribonucleoside triphosphate binding (GO:0035639) | 18 | | | 1.21E-05 |
| purine ribonucleoside binding (GO:0032550) | 18 | | | 1.31E-05 |
| purine nucleoside binding (GO:0001883) | 18 | | | 1.34E-05 |
| ribonucleoside binding (GO:0032549) | 18 | | | 1.35E-05 |
| nucleoside binding (GO:0001882) | 18 | | | 1.47E-05 |
| double-stranded RNA binding (GO:0003725) | 5 | | | 1.52E-05 |
| purine ribonucleotide binding (GO:0032555) | 18 | | | 1.68E-05 |
| ribonucleotide binding (GO:0032553) | 18 | | | 1.90E-05 |
| purine nucleotide binding (GO:0017076) | 18 | | | 1.98E-05 |
| porin activity (GO:0015288) | 3 | | | 4.71E-05 |
| anion binding (GO:0043168) | 20 | | | 1.13E-04 |
| nucleoside-triphosphatase activity (GO:0017111) | 11 | | | 1.26E-04 |
| enzyme binding (GO:0019899) | 14 | | | 1.80E-04 |
| pyrophosphatase activity (GO:0016462) | 11 | | | 2.17E-04 |
| hydrolase activity, acting on acid anhydrides, in phosphorus-containing anhydrides (GO:0016818) | 11 | | | 2.22E-04 |
| hydrolase activity, acting on acid anhydrides (GO:0016817) | 11 | | | 2.33E-04 |
| wide pore channel activity (GO:0022829) | 3 | | | 1.24E-03 |
| voltage-gated anion channel activity (GO:0008308) | 3 | | | 1.24E-03 |
| structure-specific DNA binding (GO:0043566) | 6 | | | 1.59E-03 |
| single-stranded DNA binding (GO:0003697) | 4 | | | 1.88E-03 |
| anion transmembrane transporter activity (GO:0008509) | 6 | | | 2.01E-03 |
| phospholipase A2 inhibitor activity (GO:0019834) | 2 | | | 2.68E-03 |
| substrate-specific transmembrane transporter activity (GO:0022891) | 10 | | | 2.83E-03 |
| ATP binding (GO:0005524) | 13 | | | 2.92E-03 |
| adenyl ribonucleotide binding (GO:0032559) | 13 | | | 3.68E-03 |
| adenyl nucleotide binding (GO:0030554) | 13 | | | 4.14E-03 |
| ribosome binding (GO:0043022) | 3 | | | 4.30E-03 |
| structural constituent of cytoskeleton (GO:0005200) | 4 | | | 5.30E-03 |
| transmembrane transporter activity (GO:0022857) | 10 | | | 6.02E-03 |
| structural molecule activity (GO:0005198) | 8 | | | 7.73E-03 |
| ion transmembrane transporter activity (GO:0015075) | 9 | | | 8.63E-03 |
| substrate-specific transporter activity (GO:0022892) | 10 | | | 9.53E-03 |
| **META INPUT LIST** | | | | |
| **Pathway name** | | **Gene** | **P-value** | |
| molecular_function (GO:0003674) | | 461 | 3.12E-56 | |
| protein binding (GO:0005515) | | 342 | 8.36E-52 | |
| binding (GO:0005488) | | 407 | 4.18E-43 | |
| catalytic activity (GO:0003824) | | 229 | 3.70E-27 | |
| small molecule binding (GO:0036094) | | 136 | 8.76E-22 | |
| anion binding (GO:0043168) | | 134 | 3.42E-21 | |
| RNA binding (GO:0003723) | | 91 | 6.79E-17 | |
| nucleotide binding (GO:0000166) | | 117 | 6.99E-17 | |
| nucleoside phosphate binding (GO:1901265) | | 117 | 7.23E-17 | |
| nucleoside-triphosphatase activity (GO:0017111) | | 61 | 1.52E-16 | |
| poly(A) RNA binding (GO:0044822) | | 76 | 2.78E-16 | |
| pyrophosphatase activity (GO:0016462) | | 62 | 5.15E-16 | |
| hydrolase activity, acting on acid anhydrides, in phosphorus-containing anhydrides (GO:0016818) | | 62 | 5.81E-16 | |
| hydrolase activity, acting on acid anhydrides (GO:0016817) | | 62 | 7.39E-16 | |
| purine nucleotide binding (GO:0017076) | | 97 | 2.70E-14 | |
| purine ribonucleoside triphosphate binding (GO:0035639) | | 95 | 2.86E-14 | |
| carbohydrate derivative binding (GO:0097367) | | 107 | 2.92E-14 | |
| protein complex binding (GO:0032403) | | 58 | 3.65E-14 | |
| purine ribonucleotide binding (GO:0032555) | | 96 | 3.89E-14 | |
| purine ribonucleoside binding (GO:0032550) | | 95 | 3.93E-14 | |
| purine nucleoside binding (GO:0001883) | | 95 | 4.37E-14 | |
| ribonucleoside binding (GO:0032549) | | 95 | 4.53E-14 | |
| nucleoside binding (GO:0001882) | | 95 | 6.42E-14 | |
| ribonucleotide binding (GO:0032553) | | 96 | 6.54E-14 | |
| cytoskeletal protein binding (GO:0008092) | | 56 | 9.15E-14 | |
| enzyme binding (GO:0019899) | | 76 | 5.70E-13 | |
| oxidoreductase activity (GO:0016491) | | 54 | 7.63E-13 | |
| hydrolase activity (GO:0016787) | | 110 | 1.35E-12 | |
| organic cyclic compound binding (GO:0097159) | | 200 | 2.38E-12 | |
| oxidoreductase activity, acting on NAD(P)H, quinone or similar compound as acceptor (GO:0016655) | | 17 | 1.04E-11 | |
| GTPase activity (GO:0003924) | | 28 | 1.09E-11 | |
| heterocyclic compound binding (GO:1901363) | | 196 | 1.30E-11 | |
| structural molecule activity (GO:0005198) | | 45 | 2.77E-10 | |
| oxidoreductase activity, acting on NAD(P)H (GO:0016651) | | 19 | 3.83E-10 | |
| NADH dehydrogenase (quinone) activity (GO:0050136) | | 14 | 6.83E-10 | |
| NADH dehydrogenase activity (GO:0003954) | | 14 | 6.83E-10 | |
| NADH dehydrogenase (ubiquinone) activity (GO:0008137) | | 14 | 6.83E-10 | |
| ion binding (GO:0043167) | | 192 | 5.36E-09 | |
| structural constituent of cytoskeleton (GO:0005200) | | 17 | 1.22E-08 | |
| ATP binding (GO:0005524) | | 71 | 1.83E-08 | |
| adenyl nucleotide binding (GO:0030554) | | 72 | 3.21E-08 | |
| adenyl ribonucleotide binding (GO:0032559) | | 71 | 4.91E-08 | |
| identical protein binding (GO:0042802) | | 55 | 2.82E-07 | |
| substrate-specific transporter activity (GO:0022892) | | 53 | 3.59E-07 | |
| transporter activity (GO:0005215) | | 60 | 4.58E-07 | |
| guanyl ribonucleotide binding (GO:0032561) | | 30 | 6.93E-07 | |
| guanyl nucleotide binding (GO:0019001) | | 30 | 7.35E-07 | |
| GTP binding (GO:0005525) | | 29 | 7.89E-07 | |
| unfolded protein binding (GO:0051082) | | 15 | 1.19E-06 | |
| ATPase activity, coupled to transmembrane movement of ions (GO:0042625) | | 13 | 1.40E-06 | |
| calmodulin binding (GO:0005516) | | 19 | 2.30E-06 | |
| enzyme regulator activity (GO:0030234) | | 46 | 2.93E-06 | |
| substrate-specific transmembrane transporter activity (GO:0022891) | | 46 | 4.02E-06 | |
| ATPase activity (GO:0016887) | | 27 | 4.26E-06 | |
| electron carrier activity (GO:0009055) | | 15 | 4.39E-06 | |
| active transmembrane transporter activity (GO:0022804) | | 25 | 6.09E-06 | |
| transmembrane transporter activity (GO:0022857) | | 48 | 8.36E-06 | |
| ion transmembrane transporter activity (GO:0015075) | | 43 | 9.35E-06 | |
| cation-transporting ATPase activity (GO:0019829) | | 12 | 9.36E-06 | |
| tubulin binding (GO:0015631) | | 21 | 1.06E-05 | |
| monovalent inorganic cation transmembrane transporter activity (GO:0015077) | | 26 | 1.10E-05 | |
| SNARE binding (GO:0000149) | | 11 | 1.36E-05 | |
| hydrogen ion transmembrane transporter activity (GO:0015078) | | 14 | 1.90E-05 | |
| actin binding (GO:0003779) | | 27 | 2.25E-05 | |
| ATPase activity, coupled to transmembrane movement of substances (GO:0042626) | | 14 | 2.70E-05 | |
| protein phosphatase regulator activity (GO:0019888) | | 11 | 2.72E-05 | |
| ATPase activity, coupled to movement of substances (GO:0043492) | | 14 | 3.03E-05 | |
| hydrolase activity, acting on acid anhydrides, catalyzing transmembrane movement of substances (GO:0016820) | | 14 | 3.39E-05 | |
| inorganic cation transmembrane transporter activity (GO:0022890) | | 31 | 3.82E-05 | |
| P-P-bond-hydrolysis-driven transmembrane transporter activity (GO:0015405) | | 14 | 5.87E-05 | |
| primary active transmembrane transporter activity (GO:0015399) | | 14 | 5.87E-05 | |
| protein phosphatase type 2A regulator activity (GO:0008601) | | 7 | 9.93E-05 | |
| phosphatase regulator activity (GO:0019208) | | 11 | 1.09E-04 | |
| G-protein coupled receptor activity (GO:0004930) | | 1 | 1.18E-04 | |
| kinase binding (GO:0019900) | | 29 | 1.24E-04 | |
| cation transmembrane transporter activity (GO:0008324) | | 34 | 1.28E-04 | |
| ATPase activity, coupled to transmembrane movement of ions, phosphorylative mechanism (GO:0015662) | | 8 | 1.42E-04 | |
| carboxylic acid binding (GO:0031406) | | 18 | 1.56E-04 | |
| organic acid binding (GO:0043177) | | 18 | 1.68E-04 | |
| syntaxin-1 binding (GO:0017075) | | 6 | 3.22E-04 | |
| cofactor binding (GO:0048037) | | 20 | 3.24E-04 | |
| lyase activity (GO:0016829) | | 16 | 3.49E-04 | |
| protein domain specific binding (GO:0019904) | | 31 | 6.89E-04 | |
| receptor activity (GO:0004872) | | 10 | 9.51E-04 | |
| transmembrane signaling receptor activity (GO:0004888) | | 6 | 1.01E-03 | |
| syntaxin binding (GO:0019905) | | 8 | 1.10E-03 | |
| microtubule binding (GO:0008017) | | 15 | 1.21E-03 | |
| amino acid binding (GO:0016597) | | 12 | 1.41E-03 | |
| beta-tubulin binding (GO:0048487) | | 7 | 1.41E-03 | |
| ATPase activity, coupled (GO:0042623) | | 19 | 1.90E-03 | |
| cation:amino acid symporter activity (GO:0005416) | | 5 | 2.11E-03 | |
| signaling receptor activity (GO:0038023) | | 8 | 2.86E-03 | |
| protein kinase binding (GO:0019901) | | 24 | 4.62E-03 | |
| porin activity (GO:0015288) | | 4 | 5.25E-03 | |
| MHC class II protein complex binding (GO:0023026) | | 5 | 6.11E-03 | |
| MHC protein complex binding (GO:0023023) | | 5 | 6.11E-03 | |
| sodium ion transmembrane transporter activity (GO:0015081) | | 12 | 6.96E-03 | |
| GTPase binding (GO:0051020) | | 15 | 8.31E-03 | |

**Table 4**: Cellular Component Gene Ontology categories significantly over-represented in the META input list. Analysis performed using the GO Consortium platform (http://geneontology.org/).

| **META INPUT LIST** | | |
| --- | --- | --- |
| **Pathway name** | **Gene** | **P-value** |
| vesicle (GO:0031982) | 239 | 2.66E-75 |
| extracellular organelle (GO:0043230) | 210 | 1.37E-74 |
| extracellular vesicular exosome (GO:0070062) | 210 | 1.37E-74 |
| extracellular membrane-bounded organelle (GO:0065010) | 210 | 1.37E-74 |
| membrane-bounded vesicle (GO:0031988) | 235 | 2.82E-74 |
| cytoplasmic part (GO:0044444) | 344 | 4.24E-71 |
| cytoplasm (GO:0005737) | 393 | 9.93E-70 |
| extracellular region part (GO:0044421) | 227 | 2.05E-61 |
| intracellular (GO:0005622) | 427 | 2.28E-55 |
| intracellular part (GO:0044424) | 424 | 1.01E-54 |
| cell part (GO:0044464) | 453 | 1.88E-54 |
| cell (GO:0005623) | 453 | 1.99E-54 |
| organelle (GO:0043226) | 409 | 3.08E-51 |
| extracellular region (GO:0005576) | 232 | 3.32E-49 |
| membrane-bounded organelle (GO:0043227) | 389 | 2.79E-48 |
| organelle part (GO:0044422) | 310 | 8.31E-48 |
| intracellular organelle part (GO:0044446) | 303 | 4.71E-46 |
| cellular_component (GO:0005575) | 465 | 1.49E-44 |
| cytosol (GO:0005829) | 177 | 1.21E-43 |
| macromolecular complex (GO:0032991) | 228 | 4.82E-41 |
| intracellular organelle (GO:0043229) | 376 | 1.02E-39 |
| protein complex (GO:0043234) | 205 | 9.01E-39 |
| mitochondrial part (GO:0044429) | 92 | 8.11E-37 |
| mitochondrion (GO:0005739) | 124 | 9.06E-36 |
| intracellular membrane-bounded organelle (GO:0043231) | 341 | 1.55E-31 |
| neuron part (GO:0097458) | 86 | 1.30E-29 |
| cell projection (GO:0042995) | 107 | 1.97E-28 |
| organelle inner membrane (GO:0019866) | 59 | 1.29E-27 |
| neuron projection (GO:0043005) | 75 | 1.72E-27 |
| mitochondrial inner membrane (GO:0005743) | 56 | 3.58E-27 |
| mitochondrial envelope (GO:0005740) | 66 | 3.82E-26 |
| mitochondrial membrane (GO:0031966) | 64 | 4.15E-26 |
| intracellular non-membrane-bounded organelle (GO:0043232) | 181 | 5.07E-21 |
| non-membrane-bounded organelle (GO:0043228) | 181 | 5.07E-21 |
| organelle envelope (GO:0031967) | 74 | 1.21E-20 |
| envelope (GO:0031975) | 74 | 1.57E-20 |
| membrane (GO:0016020) | 282 | 1.03E-19 |
| mitochondrial matrix (GO:0005759) | 43 | 6.52E-19 |
| organelle membrane (GO:0031090) | 132 | 1.63E-18 |
| cytoskeletal part (GO:0044430) | 87 | 1.60E-17 |
| cytoskeleton (GO:0005856) | 104 | 5.50E-17 |
| membrane-enclosed lumen (GO:0031974) | 149 | 2.15E-15 |
| axon (GO:0030424) | 35 | 1.57E-14 |
| organelle lumen (GO:0043233) | 143 | 1.10E-13 |
| cell body (GO:0044297) | 38 | 4.08E-13 |
| intracellular organelle lumen (GO:0070013) | 139 | 8.75E-13 |
| oxidoreductase complex (GO:1990204) | 20 | 2.62E-12 |
| somatodendritic compartment (GO:0036477) | 44 | 3.69E-12 |
| cell periphery (GO:0071944) | 168 | 7.37E-12 |
| microtubule cytoskeleton (GO:0015630) | 60 | 7.46E-12 |
| cytoplasmic vesicle (GO:0031410) | 64 | 1.56E-11 |
| nucleus (GO:0005634) | 209 | 1.75E-11 |
| melanosome (GO:0042470) | 20 | 1.94E-11 |
| pigment granule (GO:0048770) | 20 | 1.94E-11 |
| synapse (GO:0045202) | 44 | 1.98E-11 |
| mitochondrial membrane part (GO:0044455) | 24 | 4.62E-11 |
| respiratory chain (GO:0070469) | 18 | 1.45E-10 |
| cytoplasmic membrane-bounded vesicle (GO:0016023) | 59 | 1.74E-10 |
| respiratory chain complex I (GO:0045271) | 14 | 4.41E-10 |
| NADH dehydrogenase complex (GO:0030964) | 14 | 4.41E-10 |
| mitochondrial respiratory chain complex I (GO:0005747) | 14 | 4.41E-10 |
| plasma membrane (GO:0005886) | 160 | 4.68E-10 |
| cell projection part (GO:0044463) | 46 | 8.37E-10 |
| perinuclear region of cytoplasm (GO:0048471) | 40 | 3.77E-09 |
| neuronal cell body (GO:0043025) | 30 | 5.24E-09 |
| catalytic complex (GO:1902494) | 50 | 1.32E-08 |
| mitochondrial respiratory chain (GO:0005746) | 15 | 1.72E-08 |
| COP9 signalosome (GO:0008180) | 10 | 1.37E-06 |
| microtubule (GO:0005874) | 28 | 2.02E-06 |
| axon part (GO:0033267) | 16 | 2.60E-06 |
| neuron projection terminus (GO:0044306) | 13 | 2.78E-06 |
| clathrin-coated vesicle membrane (GO:0030665) | 13 | 2.78E-06 |
| synapse part (GO:0044456) | 29 | 2.90E-06 |
| dendrite (GO:0030425) | 27 | 5.54E-06 |
| cell cortex (GO:0005938) | 20 | 1.22E-05 |
| cytosolic part (GO:0044445) | 19 | 1.46E-05 |
| actin cytoskeleton (GO:0015629) | 27 | 2.80E-05 |
| coated pit (GO:0005905) | 11 | 3.86E-05 |
| cytoplasmic vesicle part (GO:0044433) | 31 | 4.13E-05 |
| mitochondrial nucleoid (GO:0042645) | 9 | 6.04E-05 |
| axon terminus (GO:0043679) | 11 | 6.12E-05 |
| nucleoid (GO:0009295) | 9 | 9.12E-05 |
| endocytic vesicle (GO:0030139) | 18 | 1.27E-04 |
| nuclear part (GO:0044428) | 106 | 1.56E-04 |
| clathrin-sculpted gamma-aminobutyric acid transport vesicle membrane (GO:0061202) | 5 | 2.59E-04 |
| clathrin-sculpted gamma-aminobutyric acid transport vesicle (GO:0061200) | 5 | 2.59E-04 |
| coated vesicle (GO:0030135) | 16 | 3.03E-04 |
| plasma membrane part (GO:0044459) | 79 | 3.12E-04 |
| coated vesicle membrane (GO:0030662) | 13 | 3.77E-04 |
| blood microparticle (GO:0072562) | 13 | 4.15E-04 |
| eukaryotic translation elongation factor 1 complex (GO:0005853) | 4 | 5.12E-04 |
| cell cortex part (GO:0044448) | 12 | 5.61E-04 |
| clathrin-coated vesicle (GO:0030136) | 13 | 7.21E-04 |
| cell leading edge (GO:0031252) | 21 | 9.69E-04 |
| presynaptic membrane (GO:0042734) | 9 | 1.14E-03 |
| protein serine/threonine phosphatase complex (GO:0008287) | 8 | 1.14E-03 |
| phosphatase complex (GO:1903293) | 8 | 1.14E-03 |
| synaptic vesicle (GO:0008021) | 12 | 1.34E-03 |
| protein phosphatase type 2A complex (GO:0000159) | 6 | 1.44E-03 |
| clathrin coat (GO:0030118) | 8 | 1.59E-03 |
| cortical cytoskeleton (GO:0030863) | 9 | 1.72E-03 |
| clathrin coat of coated pit (GO:0030132) | 5 | 1.83E-03 |
| clathrin-sculpted vesicle (GO:0060198) | 5 | 1.83E-03 |
| cytoplasmic vesicle membrane (GO:0030659) | 23 | 2.42E-03 |
| mitochondrial proton-transporting ATP synthase complex (GO:0005753) | 6 | 2.45E-03 |
| proton-transporting two-sector ATPase complex (GO:0016469) | 8 | 2.97E-03 |
| proton-transporting ATP synthase complex (GO:0045259) | 6 | 3.15E-03 |
| heterotrimeric G-protein complex (GO:0005834) | 7 | 3.29E-03 |
| cortical actin cytoskeleton (GO:0030864) | 7 | 3.29E-03 |
| nuclear lumen (GO:0031981) | 92 | 3.35E-03 |
| vesicle membrane (GO:0012506) | 23 | 4.11E-03 |
| extracellular space (GO:0005615) | 48 | 4.24E-03 |
| bounding membrane of organelle (GO:0098588) | 72 | 4.71E-03 |
| cell junction (GO:0030054) | 37 | 5.07E-03 |
| endocytic vesicle membrane (GO:0030666) | 12 | 5.60E-03 |
| nuclear lamina (GO:0005652) | 4 | 7.65E-03 |
| midbody (GO:0030496) | 11 | 9.95E-03 |
| organelle envelope lumen (GO:0031970) | 9 | 9.98E-03 |

**Table 5**: Biochemical pathways significantly over-represented in the EXP and META input lists. Analysis performed using Reactome (http://www.reactome.org/).

| **EXP INPUT LIST** | | |
| --- | --- | --- |
| **Pathway name** | **Gene** | **P-value** |
| Attenuation phase (REACT_200624) | 14 | 7.16E-14 |
| HSF1 activation (REACT_200744) | 13 | 7.16E-14 |
| HSF1-dependent transactivation (REACT_200775) | 14 | 7.16E-14 |
| Cellular response to heat stress (REACT_200819) | 15 | 1.25E-13 |
| Cellular responses to stress (REACT_120956) | 23 | 2.16E-12 |
| Regulation of HSF1-mediated heat shock response (REACT_200780) | 12 | 2.82E-11 |
| Post-chaperonin tubulin folding pathway (REACT_16967) | 6 | 3.94E-06 |
| Gap junction trafficking (REACT_9411) | 7 | 1.77E-05 |
| Recycling pathway of L1 (REACT_22365) | 7 | 2.29E-05 |
| Microtubule-dependent trafficking of connexons from Golgi to the plasma membrane (REACT_11039) | 5 | 2.29E-05 |
| Gap junction trafficking and regulation (REACT_9480) | 7 | 2.29E-05 |
| Transport of connexons to the plasma membrane (REACT_11050) | 5 | 2.61E-05 |
| Mitochondrial protein import (REACT_118595) | 7 | 4.48E-05 |
| M Phase (REACT_910) | 14 | 4.48E-05 |
| Protein folding (REACT_16952) | 7 | 5.93E-05 |
| Recruitment of NuMA to mitotic centrosomes (REACT_15510) | 5 | 2.49E-04 |
| Translocation of GLUT4 to the plasma membrane (REACT_147867) | 7 | 4.22E-04 |
| Cooperation of Prefoldin and TriC/CCT in actin and tubulin folding (REACT_17029) | 5 | 4.22E-04 |
| Packaging Of Telomere Ends (REACT_7963) | 5 | 4.63E-04 |
| Gap junction assembly (REACT_9509) | 5 | 5.20E-04 |
| Meiotic synapsis (REACT_75792) | 6 | 6.95E-04 |
| Metabolism of proteins (REACT_17015) | 21 | 7.69E-04 |
| Mitotic Prophase (REACT_765) | 8 | 7.89E-04 |
| DNA Damage/Telomere Stress Induced Senescence (REACT_169185) | 6 | 7.89E-04 |
| Cell Cycle, Mitotic (REACT_152) | 15 | 1.35E-03 |
| Kinesins (REACT_25201) | 5 | 1.52E-03 |
| Formation of tubulin folding intermediates by CCT/TriC (REACT_16956) | 4 | 1.91E-03 |
| Mitotic Anaphase (REACT_1275) | 9 | 1.94E-03 |
| Mitotic Metaphase and Anaphase (REACT_150314) | 9 | 1.94E-03 |
| Glucose metabolism (REACT_723) | 7 | 2.05E-03 |
| L1CAM interactions (REACT_22205) | 7 | 2.53E-03 |
| Chaperonin-mediated protein folding (REACT_17004) | 5 | 2.55E-03 |
| Centrosome maturation (REACT_15479) | 6 | 2.55E-03 |
| Recruitment of mitotic centrosome proteins and complexes (REACT_15296) | 6 | 2.55E-03 |
| Condensation of Prophase Chromosomes (REACT_172744) | 5 | 2.55E-03 |
| Muscle contraction (REACT_17044) | 5 | 3.02E-03 |
| Meiosis (REACT_111183) | 6 | 3.58E-03 |
| Meiotic recombination (REACT_27271) | 5 | 3.63E-03 |
| RNA Polymerase I Promoter Opening (REACT_2232) | 4 | 3.88E-03 |
| Cell Cycle (REACT_115566) | 15 | 3.88E-03 |
| G2/M Transition (REACT_2203) | 7 | 3.93E-03 |
| Mitotic G2-G2/M phases (REACT_21391) | 7 | 4.29E-03 |
| DNA methylation (REACT_267652) | 4 | 4.62E-03 |
| Formation of the beta-catenin:TCF transactivating complex (REACT_200753) | 5 | 4.62E-03 |
| Factors involved in megakaryocyte development and platelet production (REACT_24970) | 7 | 6.46E-03 |
| Axon guidance (REACT_18266) | 11 | 6.46E-03 |
| Telomere Maintenance (REACT_7970) | 5 | 7.71E-03 |
| Vpr-mediated induction of apoptosis by mitochondrial outer membrane permeabilization (REACT_8016) | 2 | 8.09E-03 |
| PRC2 methylates histones and DNA (REACT_200808) | 4 | 8.72E-03 |
| Sema3A PAK dependent Axon repulsion (REACT_19236) | 3 | 8.72E-03 |
| SIRT1 negatively regulates rRNA Expression (REACT_200827) | 4 | 8.94E-03 |
| Transcriptional regulation by small RNAs (REACT_267668) | 5 | 8.96E-03 |
| Membrane Trafficking (REACT_11123) | 8 | 9.57E-03 |
| Deposition of new CENPA-containing nucleosomes at the centromere (REACT_22186) | 4 | 9.57E-03 |
| Nucleosome assembly (REACT_22344) | 4 | 9.57E-03 |
| Hedgehog 'off' state (REACT_267634) | 6 | 9.91E-03 |
| **META INPUT LIST** | | |
| **Pathway name** | **Gene** | **P-value** |
| Recycling pathway of L1 ((REACT_22365) | 23 | 7.31E-11 |
| L1CAM interactions (REACT_22205) | 32 | 3.39E-09 |
| Respiratory electron transport, ATP synthesis by chemiosmotic coupling, and heat production by uncoupling proteins. (REACT_6305 | 30 | 4.58E-07 |
| Gap junction trafficking (REACT_9411) | 17 | 1.22E-06 |
| Formation of tubulin folding intermediates by CCT/TriC (REACT_16956) | 13 | 1.43E-06 |
| Respiratory electron transport (REACT_22393) | 25 | 1.51E-06 |
| Gap junction trafficking and regulation (REACT_9480) | 17 | 2.21E-06 |
| Cooperation of Prefoldin and TriC/CCT in actin and tubulin folding (REACT_17029) | 14 | 2.21E-06 |
| Cellular response to heat stress (REACT_200819) | 21 | 2.33E-06 |
| Packaging Of Telomere Ends (REACT_7963) | 14 | 2.33E-06 |
| HSF1-dependent transactivation (REACT_200775) | 18 | 2.33E-06 |
| Prefoldin mediated transfer of substrate to CCT/TriC (REACT_16936) | 13 | 2.33E-06 |
| The citric acid (TCA) cycle and respiratory electron transport (REACT_111083) | 35 | 2.77E-06 |
| MHC class II antigen presentation (REACT_121399) | 28 | 3.31E-06 |
| Attenuation phase (REACT_200624) | 16 | 3.91E-06 |
| Microtubule-dependent trafficking of connexons from Golgi to the plasma membrane (REACT_11039) | 10 | 4.03E-06 |
| Hemostasis (REACT_604) | 68 | 4.58E-06 |
| Transport of connexons to the plasma membrane (REACT_11050) | 10 | 6.43E-06 |
| Cellular responses to stress (REACT_120956) | 46 | 7.51E-06 |
| HSF1 activation (REACT_200744) | 15 | 7.51E-06 |
| Kinesins (REACT_25201) | 15 | 9.50E-06 |
| Transmission across Chemical Synapses (REACT_13477) | 39 | 1.05E-05 |
| Axon guidance (REACT_18266) | 49 | 1.18E-05 |
| Translocation of GLUT4 to the plasma membrane (REACT_147867) | 20 | 1.29E-05 |
| Regulation of HSF1-mediated heat shock response (REACT_200780) | 16 | 3.12E-05 |
| Post-chaperonin tubulin folding pathway (REACT_16967) | 10 | 3.34E-05 |
| Meiotic synapsis (REACT_75792) | 16 | 7.05E-05 |
| Opioid Signalling (REACT_15295) | 21 | 9.77E-05 |
| GABA synthesis, release, reuptake and degradation (REACT_23947) | 12 | 1.15E-04 |
| DNA Damage/Telomere Stress Induced Senescence (REACT_169185) | 16 | 1.17E-04 |
| Signaling by Wnt (REACT_11045) | 42 | 1.22E-04 |
| M Phase (REACT_910) | 41 | 1.28E-04 |
| Membrane Trafficking (REACT_11123) | 32 | 1.85E-04 |
| Chaperonin-mediated protein folding (REACT_17004) | 14 | 2.27E-04 |
| RNA Polymerase I Promoter Opening (REACT_2232) | 11 | 2.44E-04 |
| Formation of annular gap junctions (REACT_11049) | 7 | 2.59E-04 |
| Recruitment of NuMA to mitotic centrosomes (REACT_15510) | 10 | 3.26E-04 |
| Gap junction degradation (REACT_11035) | 7 | 4.31E-04 |
| DNA methylation (REACT_267652) | 11 | 5.06E-04 |
| Protein folding (REACT_16952) | 14 | 6.53E-04 |
| EGFR downregulation (REACT_12484) | 10 | 7.01E-04 |
| Glycolysis (REACT_1383) | 13 | 9.84E-04 |
| Glucose metabolism (REACT_723) | 20 | 1.01E-03 |
| Regulation of insulin secretion (REACT_18325) | 18 | 1.33E-03 |
| Gap junction assembly (REACT_9509) | 10 | 1.33E-03 |
| Factors involved in megakaryocyte development and platelet production (REACT_24970) | 23 | 1.93E-03 |
| Neuronal System (REACT_13685) | 39 | 2.34E-03 |
| PRC2 methylates histones and DNA (REACT_200808) | 11 | 2.36E-03 |
| Retrograde neurotrophin signalling (REACT_12435) | 7 | 2.77E-03 |
| SIRT1 negatively regulates rRNA Expression (REACT_200827) | 11 | 2.78E-03 |
| G-protein activation (REACT_15457) | 9 | 3.10E-03 |
| PLC beta mediated events (REACT_15426) | 13 | 3.15E-03 |
| Condensation of Prophase Chromosomes (REACT_172744) | 12 | 3.29E-03 |
| G-protein mediated events (REACT_15526) | 13 | 3.55E-03 |
| Nef Mediated CD8 Down-regulation (REACT_11200) | 5 | 3.63E-03 |
| Nucleosome assembly (REACT_22344) | 11 | 3.63E-03 |
| Deposition of new CENPA-containing nucleosomes at the centromere (REACT_22186) | 11 | 3.63E-03 |
| Mitotic Anaphase (REACT_1275) | 26 | 3.81E-03 |
| Mitochondrial protein import (REACT_118595) | 12 | 3.81E-03 |
| ADP signalling through P2Y purinoceptor 12 (REACT_20653) | 8 | 3.81E-03 |
| Adrenaline,noradrenaline inhibits insulin secretion (REACT_18339) | 9 | 3.81E-03 |
| Mitotic Metaphase and Anaphase (REACT_150314) | 26 | 3.81E-03 |
| Meiosis (REACT_111183) | 16 | 3.81E-03 |
| Gluconeogenesis (REACT_1520) | 13 | 3.81E-03 |
| formation of the beta-catenin:TCF transactivating complex (REACT_200753) | 13 | 3.81E-03 |
| Telomere Maintenance (REACT_7970) | 14 | 3.81E-03 |
| EPH-Ephrin signaling (REACT_228170) | 17 | 4.29E-03 |
| Interaction between L1 and Ankyrins (REACT_22266) | 9 | 4.46E-03 |
| Platelet homeostasis (REACT_23876) | 17 | 4.62E-03 |
| Centrosome maturation (REACT_15479) | 15 | 4.62E-03 |
| Recruitment of mitotic centrosome proteins and complexes (REACT_15296) | 15 | 4.62E-03 |
| WNT5A-dependent internalization of FZD4 (REACT_172599) | 7 | 4.85E-03 |
| Meiotic recombination (REACT_27271) | 12 | 5.13E-03 |
| Integration of energy metabolism (REACT_1505) | 20 | 5.13E-03 |
| Neurotransmitter Receptor Binding And Downstream Transmission In The Postsynaptic Cell (REACT_15370) | 22 | 6.03E-03 |
| TCF dependent signaling in response to WNT (REACT_200777) | 26 | 6.08E-03 |
| misspliced LRP5 mutants have enhanced beta-catenin-dependent signaling (REACT_228096) | 26 | 6.08E-03 |
| RNF mutants show enhanced WNT signaling and proliferation (REACT_228188) | 26 | 6.08E-03 |
| XAV939 inhibits tankyrase, stabilizing AXIN (REACT_228279) | 26 | 6.26E-03 |
| Mitotic Prophase (REACT_765) | 19 | 6.26E-03 |
| Nef Mediated CD4 Down-regulation (REACT_11166) | 5 | 6.42E-03 |
| GABA B receptor activation (REACT_25031) | 10 | 8.10E-03 |
| Activation of GABAB receptors (REACT_25330) | 10 | 8.10E-03 |
| Signal amplification (REACT_20524) | 9 | 8.10E-03 |
| EPH-ephrin mediated repulsion of cells (REACT_228189) | 11 | 8.52E-03 |
| HDACs deacetylate histones (REACT_228222) | 12 | 8.52E-03 |

**Table 6**: Biochemical pathways significantly over-represented in the EXP and META input lists. Analysis performed using the Webgestalt platform (<http://bioinfo.vanderbilt.edu/webgestalt/>), KEGG enrichment tool.

| **EXP INPUT LIST** | | |
| --- | --- | --- |
| **PathwayName** | **Gene** | **P-value** |
| Parkinson's disease | 8 | 1.20E-07 |
| Huntington's disease | 8 | 8.74E-07 |
| Antigen processing and presentation | 6 | 9.00E-07 |
| Protein processing in endoplasmic reticulum | 7 | 3.84E-06 |
| Pathogenic Escherichia coli infection | 4 | 1.00E-04 |
| Calcium signaling pathway | 5 | 1.00E-03 |
| Systemic lupus erythematosus | 4 | 3.10E-03 |
| Glyoxylate and dicarboxylate metabolism | 2 | 4.60E-03 |
| Alzheimer's disease | 4 | 4.80E-03 |
| **META INPUT LIST** | | |
| **PathwayName** | **Gene** | **P-value** |
| Parkinson's disease | 39 | 3.31E-30 |
| Huntington's disease | 41 | 1.51E-26 |
| Alzheimer's disease | 34 | 1.29E-20 |
| Oxidative phosphorylation | 30 | 1.02E-19 |
| Metabolic pathways | 76 | 2.57E-15 |
| Pathogenic Escherichia coli infection | 16 | 2.13E-12 |
| Endocytosis | 22 | 5.01E-08 |
| Glycolysis / Gluconeogenesis | 13 | 5.61E-08 |
| Gap junction | 13 | 2.56E-06 |
| Butanoate metabolism | 8 | 4.73E-06 |
| Phagosome | 16 | 8.12E-06 |
| Bacterial invasion of epithelial cells | 11 | 8.12E-06 |
| Calcium signaling pathway | 17 | 1.15E-05 |
| Protein processing in endoplasmic reticulum | 16 | 1.82E-05 |
| Cardiac muscle contraction | 11 | 1.82E-05 |
| Regulation of actin cytoskeleton | 18 | 2.69E-05 |
| Pyruvate metabolism | 8 | 2.97E-05 |
| Alanine, aspartate and glutamate metabolism | 7 | 6.15E-05 |
| Antigen processing and presentation | 10 | 8.52E-05 |
| Oocyte meiosis | 12 | 9.51E-05 |
| Pentose phosphate pathway | 6 | 2.00E-04 |
| Phenylalanine metabolism | 5 | 2.00E-04 |
| Fc gamma R-mediated phagocytosis | 10 | 5.00E-04 |
| beta-Alanine metabolism | 5 | 6.00E-04 |
| mRNA surveillance pathway | 9 | 6.00E-04 |
| Fructose and mannose metabolism | 6 | 1.10E-03 |
| Arginine and proline metabolism | 7 | 1.60E-03 |
| Tyrosine metabolism | 6 | 2.00E-03 |
| Valine, leucine and isoleucine degradation | 6 | 2.40E-03 |
| Citrate cycle (TCA cycle) | 5 | 2.80E-03 |
| Neurotrophin signaling pathway | 10 | 4.10E-03 |
| Tight junction | 10 | 5.20E-03 |
| Toxoplasmosis | 10 | 5.20E-03 |
| Prion diseases | 5 | 5.40E-03 |
| Systemic lupus erythematosus | 10 | 5.90E-03 |
| Amyotrophic lateral sclerosis (ALS) | 6 | 5.90E-03 |
| Gastric acid secretion | 7 | 6.90E-03 |

**Table 7**: Biochemical pathways significantly over-represented in the EXP and META input lists. Analysis performed using the Webgestalt platform (<http://bioinfo.vanderbilt.edu/webgestalt/>), Wikipathways enrichment tool.

| **EXP INPUT LIST** | | |
| --- | --- | --- |
| **PathwayName** | **Gene** | **P-value** |
| [Parkin-Ubiquitin Proteasomal System pathway](http://bioinfo.vanderbilt.edu/webgestalt/draw_wikipathway.php?organism=hsapiens&wp_id=WP2359&values=NA,NA,NA,NA,NA,NA,NA&enriched_ids=3305,84790,3313,3303,3309,10376,3310&timestamp=1404052282) | [7](http://bioinfo.vanderbilt.edu/webgestalt/htdocs/final_wiki_geneset_file_1404052282.html#Parkin-Ubiquitin Proteasomal System pathway) | 2.16e-08 |
| [Electron Transport Chain](http://bioinfo.vanderbilt.edu/webgestalt/draw_wikipathway.php?organism=hsapiens&wp_id=WP111&values=NA,NA,NA,NA,NA&enriched_ids=4722,498,292,10476,293&timestamp=1404052282) | [5](http://bioinfo.vanderbilt.edu/webgestalt/htdocs/final_wiki_geneset_file_1404052282.html#Electron Transport Chain) | 0.0001 |
| [Pathogenic Escherichia coli infection](http://bioinfo.vanderbilt.edu/webgestalt/draw_wikipathway.php?organism=hsapiens&wp_id=WP2272&values=NA,NA,NA,NA&enriched_ids=60,84790,4691,10376&timestamp=1404052282) | [4](http://bioinfo.vanderbilt.edu/webgestalt/htdocs/final_wiki_geneset_file_1404052282.html#Pathogenic Escherichia coli infection) | 0.0002 |
| [Glycolysis and Gluconeogenesis](http://bioinfo.vanderbilt.edu/webgestalt/draw_wikipathway.php?organism=hsapiens&wp_id=WP534&values=NA,NA,NA&enriched_ids=2597,5315,4191&timestamp=1404052282) | [3](http://bioinfo.vanderbilt.edu/webgestalt/htdocs/final_wiki_geneset_file_1404052282.html#Glycolysis and Gluconeogenesis) | 0.0017 |
| [Oxidative phosphorylation](http://bioinfo.vanderbilt.edu/webgestalt/draw_wikipathway.php?organism=hsapiens&wp_id=WP623&values=NA,NA,NA&enriched_ids=4722,498,10476&timestamp=1404052282) | 3 | 0.0024 |
| [Lymphocyte TarBase](http://bioinfo.vanderbilt.edu/webgestalt/draw_wikipathway.php?organism=hsapiens&wp_id=WP2004&values=NA,NA,NA,NA,NA,NA,NA&enriched_ids=94081,4691,302,5315,10487,708,10165&timestamp=1404052282) | 7 | 0.0026 |
| [Leukocyte TarBase](http://bioinfo.vanderbilt.edu/webgestalt/draw_wikipathway.php?organism=hsapiens&wp_id=WP2003&values=NA,NA,NA,NA&enriched_ids=94081,302,5315,10487&timestamp=1404052282) | [4](http://bioinfo.vanderbilt.edu/webgestalt/htdocs/final_wiki_geneset_file_1404052282.html#Leukocyte TarBase) | 0.0029 |
| [TCA Cycle](http://bioinfo.vanderbilt.edu/webgestalt/draw_wikipathway.php?organism=hsapiens&wp_id=WP78&values=NA,NA&enriched_ids=4191,1431&timestamp=1404052282) | [2](http://bioinfo.vanderbilt.edu/webgestalt/htdocs/final_wiki_geneset_file_1404052282.html#TCA Cycle) | 0.0032 |
| [Prostaglandin Synthesis and Regulation](http://bioinfo.vanderbilt.edu/webgestalt/draw_wikipathway.php?organism=hsapiens&wp_id=WP98&values=NA,NA&enriched_ids=306,302&timestamp=1404052282) | [2](http://bioinfo.vanderbilt.edu/webgestalt/htdocs/final_wiki_geneset_file_1404052282.html#Prostaglandin Synthesis and Regulation) | 0.0070 |
| **META INPUT LIST** | | |
| **PathwayName** | **Gene** | **P-value** |
| Synaptic Vesicle Pathway | 23 | 1.25E-21 |
| Electron Transport Chain | 28 | 1.62E-20 |
| Parkin-Ubiquitin Proteasomal System pathway | 23 | 4.82E-19 |
| Oxidative phosphorylation | 16 | 8.47E-12 |
| Glycolysis and Gluconeogenesis | 15 | 8.47E-12 |
| Pathogenic Escherichia coli infection | 16 | 2.11E-11 |
| Calcium Regulation in the Cardiac Cell | 20 | 6.77E-09 |
| Glial Cell Differentiation | 5 | 1.87E-06 |
| G Protein Signaling Pathways | 13 | 4.08E-06 |
| EGF-EGFR Signaling Pathway | 17 | 7.30E-06 |
| SIDS Susceptibility Pathways | 18 | 3.06E-05 |
| Regulation of Actin Cytoskeleton | 15 | 3.56E-05 |
| Insulin Signaling | 15 | 5.66E-05 |
| Myometrial Relaxation and Contraction Pathways | 15 | 5.66E-05 |
| Biogenic Amine Synthesis | 5 | 3.00E-04 |
| Leukocyte TarBase | 13 | 6.00E-04 |
| Nicotine Activity on Dopaminergic Neurons | 6 | 6.00E-04 |
| Alanine and aspartate metabolism | 4 | 1.10E-03 |
| Squamous cell TarBase | 12 | 1.10E-03 |
| Acetylcholine Synthesis | 3 | 2.00E-03 |
| Lymphocyte TarBase | 26 | 2.00E-03 |
| Alzheimers Disease | 8 | 2.80E-03 |
| Proteasome Degradation | 7 | 3.20E-03 |
| Serotonin HTR1 Group and FOS Pathway | 5 | 3.50E-03 |
| Epithelium TarBase | 18 | 5.00E-03 |
| Translation Factors | 6 | 5.00E-03 |
| TSH signaling pathway | 7 | 5.00E-03 |
| G13 Signaling Pathway | 5 | 6.10E-03 |
| Glycogen Metabolism | 5 | 6.10E-03 |

**Table 8**: Biochemical pathways significantly over-represented in the EXP and META input lists. Analysis performed using the Webgestalt platform (<http://bioinfo.vanderbilt.edu/webgestalt/>), Pathways Commons enrichment tool.

| **EXP INPUT LIST** | | |
| --- | --- | --- |
| **PathwayName** | **Gene** | **P-value** |
| [Platelet degranulation](http://www.pathwaycommons.org/pc/webservice.do?version=3.0&snapshot_id=GLOBAL_FILTER_SETTINGS&record_type=PATHWAY&q=%27Platelet%20degranulation%27&format=html&cmd=get_by_keyword) | [4](http://bioinfo.vanderbilt.edu/webgestalt/htdocs/final_pc_geneset_file_1404052282.html#Platelet degranulation) | 0.0002 |
| [Response to elevated platelet cytosolic Ca2+](http://www.pathwaycommons.org/pc/webservice.do?version=3.0&snapshot_id=GLOBAL_FILTER_SETTINGS&record_type=PATHWAY&q=%27Response%20to%20elevated%20platelet%20cytosolic%20Ca2+%27&format=html&cmd=get_by_keyword) | [4](http://bioinfo.vanderbilt.edu/webgestalt/htdocs/final_pc_geneset_file_1404052282.html#Response to elevated platelet cytosolic Ca2+) | 0.0003 |
| [The citric acid (TCA) cycle and respiratory electron transport](http://www.pathwaycommons.org/pc/webservice.do?version=3.0&snapshot_id=GLOBAL_FILTER_SETTINGS&record_type=PATHWAY&q=%27The%20citric%20acid%20(TCA)%20cycle%20and%20respiratory%20electron%20transport%27&format=html&cmd=get_by_keyword) | 5 | 0.0008 |
| [Vpr-mediated induction of apoptosis by mitochondrial outer membrane permeabilization](http://www.pathwaycommons.org/pc/webservice.do?version=3.0&snapshot_id=GLOBAL_FILTER_SETTINGS&record_type=PATHWAY&q=%27Vpr-mediated%20induction%20of%20apoptosis%20by%20mitochondrial%20outer%20membrane%20permeabilization%27&format=html&cmd=get_by_keyword) | 2 | 0.0008 |
| [Validated targets of C-MYC transcriptional activation](http://www.pathwaycommons.org/pc/webservice.do?version=3.0&snapshot_id=GLOBAL_FILTER_SETTINGS&record_type=PATHWAY&q=%27Validated%20targets%20of%20C-MYC%20transcriptional%20activation%27&format=html&cmd=get_by_keyword) | [4](http://bioinfo.vanderbilt.edu/webgestalt/htdocs/final_pc_geneset_file_1404052282.html#Validated targets of C-MYC transcriptional activation) | 0.0023 |
| [Meiotic Synapsis](http://www.pathwaycommons.org/pc/webservice.do?version=3.0&snapshot_id=GLOBAL_FILTER_SETTINGS&record_type=PATHWAY&q=%27Meiotic%20Synapsis%27&format=html&cmd=get_by_keyword) | [3](http://bioinfo.vanderbilt.edu/webgestalt/htdocs/final_pc_geneset_file_1404052282.html#Meiotic Synapsis) | 0.0053 |
| [Activation of Chaperones by ATF6-alpha](http://www.pathwaycommons.org/pc/webservice.do?version=3.0&snapshot_id=GLOBAL_FILTER_SETTINGS&record_type=PATHWAY&q=%27Activation%20of%20Chaperones%20by%20ATF6-alpha%27&format=html&cmd=get_by_keyword) | [2](http://bioinfo.vanderbilt.edu/webgestalt/htdocs/final_pc_geneset_file_1404052282.html#Activation of Chaperones by ATF6-alpha) | 0.0053 |
| [Metabolism](http://www.pathwaycommons.org/pc/webservice.do?version=3.0&snapshot_id=GLOBAL_FILTER_SETTINGS&record_type=PATHWAY&q=%27Diabetes%20pathways%27&format=html&cmd=get_by_keyword) | 9 | 0.0062 |
| [Influenza Infection](http://www.pathwaycommons.org/pc/webservice.do?version=3.0&snapshot_id=GLOBAL_FILTER_SETTINGS&record_type=PATHWAY&q=%27Influenza%20Infection%27&format=html&cmd=get_by_keyword) | [4](http://bioinfo.vanderbilt.edu/webgestalt/htdocs/final_pc_geneset_file_1404052282.html#Platelet activation, signaling and aggregation) | 0.0062 |
| [Platelet activation, signaling and aggregation](http://www.pathwaycommons.org/pc/webservice.do?version=3.0&snapshot_id=GLOBAL_FILTER_SETTINGS&record_type=PATHWAY&q=%27Platelet%20activation,%20signaling%20and%20aggregation%27&format=html&cmd=get_by_keyword) | [4](http://bioinfo.vanderbilt.edu/webgestalt/htdocs/final_pc_geneset_file_1404052282.html#Influenza Infection) | 0.0062 |
| Metabolism of protein | 5 | 0.0062 |
| [Formation of ATP by chemiosmotic coupling](http://www.pathwaycommons.org/pc/webservice.do?version=3.0&snapshot_id=GLOBAL_FILTER_SETTINGS&record_type=PATHWAY&q=%27Formation%20of%20ATP%20by%20chemiosmotic%20coupling%27&format=html&cmd=get_by_keyword) | 2 | 0.0062 |
| [Diabetes pathways](http://www.pathwaycommons.org/pc/webservice.do?version=3.0&snapshot_id=GLOBAL_FILTER_SETTINGS&record_type=PATHWAY&q=%27Host%20Interactions%20of%20HIV%20factors%27&format=html&cmd=get_by_keyword) | 5 | 0.0062 |
| [Sema3A PAK dependent Axon repulsion](http://www.pathwaycommons.org/pc/webservice.do?version=3.0&snapshot_id=GLOBAL_FILTER_SETTINGS&record_type=PATHWAY&q=%27Sema3A%20PAK%20dependent%20Axon%20repulsion%27&format=html&cmd=get_by_keyword) | 2 | 0.0062 |
| [Unfolded Protein Response](http://www.pathwaycommons.org/pc/webservice.do?version=3.0&snapshot_id=GLOBAL_FILTER_SETTINGS&record_type=PATHWAY&q=%27Unfolded%20Protein%20Response%27&format=html&cmd=get_by_keyword) | 3 | 0.0062 |
| Host Interactions of HIV factors | 4 | 0.0062 |
| [C-MYC pathway](http://www.pathwaycommons.org/pc/webservice.do?version=3.0&snapshot_id=GLOBAL_FILTER_SETTINGS&record_type=PATHWAY&q=%27C-MYC%20pathway%27&format=html&cmd=get_by_keyword) | [2](http://bioinfo.vanderbilt.edu/webgestalt/htdocs/final_pc_geneset_file_1404052282.html#TCA cycle variation III (eukaryotic)) | 0.0062 |
| [TCA cycle variation III (eukaryotic)](http://www.pathwaycommons.org/pc/webservice.do?version=3.0&snapshot_id=GLOBAL_FILTER_SETTINGS&record_type=PATHWAY&q=%27TCA%20cycle%20variation%20III%20(eukaryotic)%27&format=html&cmd=get_by_keyword) | [4](http://bioinfo.vanderbilt.edu/webgestalt/htdocs/final_pc_geneset_file_1404052282.html#C-MYC pathway) | 0.0065 |
| [Meiosis](http://www.pathwaycommons.org/pc/webservice.do?version=3.0&snapshot_id=GLOBAL_FILTER_SETTINGS&record_type=PATHWAY&q=%27Meiosis%27&format=html&cmd=get_by_keyword) | [3](http://bioinfo.vanderbilt.edu/webgestalt/htdocs/final_pc_geneset_file_1404052282.html#Meiosis) | 0.0078 |
| [Citric acid cycle (TCA cycle)](http://www.pathwaycommons.org/pc/webservice.do?version=3.0&snapshot_id=GLOBAL_FILTER_SETTINGS&record_type=PATHWAY&q=%27Citric%20acid%20cycle%20(TCA%20cycle)%27&format=html&cmd=get_by_keyword) | [2](http://bioinfo.vanderbilt.edu/webgestalt/htdocs/final_pc_geneset_file_1404052282.html#Citric acid cycle (TCA cycle)) | 0.0087 |
| [Gluconeogenesis](http://www.pathwaycommons.org/pc/webservice.do?version=3.0&snapshot_id=GLOBAL_FILTER_SETTINGS&record_type=PATHWAY&q=%27Gluconeogenesis%27&format=html&cmd=get_by_keyword) | [2](http://bioinfo.vanderbilt.edu/webgestalt/htdocs/final_pc_geneset_file_1404052282.html#Gluconeogenesis) | 0.0089 |
| [Packaging Of Telomere Ends](http://www.pathwaycommons.org/pc/webservice.do?version=3.0&snapshot_id=GLOBAL_FILTER_SETTINGS&record_type=PATHWAY&q=%27Packaging%20Of%20Telomere%20Ends%27&format=html&cmd=get_by_keyword) | [2](http://bioinfo.vanderbilt.edu/webgestalt/htdocs/final_pc_geneset_file_1404052282.html#Packaging Of Telomere Ends) | 0.0096 |
| Peptide chain elongation | 3 | 0.0097 |
| **META INPUT LIST** | | |
| **PathwayName** | **Gene** | **P-value** |
| Metabolism | 83 | 4.05E-27 |
| The citric acid (TCA) cycle and respiratory electron transport | 32 | 9.16E-23 |
| Respiratory electron transport, ATP synthesis by chemiosmotic coupling, and heat production by uncoupling proteins. | 27 | 2.42E-20 |
| Transmission across Chemical Synapses | 29 | 3.70E-19 |
| Respiratory electron transport | 22 | 2.36E-16 |
| Neuronal System | 29 | 8.09E-14 |
| TRAIL signaling pathway | 80 | 4.86E-13 |
| GABA synthesis, release, reuptake and degradation | 11 | 8.32E-13 |
| Signaling events mediated by VEGFR1 and VEGFR2 | 77 | 2.75E-12 |
| VEGF and VEGFR signaling network | 77 | 3.41E-12 |
| Plasma membrane estrogen receptor signaling | 76 | 8.05E-12 |
| Neurotransmitter Release Cycle | 12 | 8.39E-12 |
| Signaling events mediated by focal adhesion kinase | 74 | 1.26E-11 |
| Endothelins | 75 | 1.26E-11 |
| Glypican 1 network | 75 | 1.26E-11 |
| Syndecan-1-mediated signaling events | 75 | 1.26E-11 |
| S1P1 pathway | 74 | 1.26E-11 |
| PAR1-mediated thrombin signaling events | 75 | 1.26E-11 |
| Sphingosine 1-phosphate (S1P) pathway | 75 | 1.26E-11 |
| Arf6 trafficking events | 74 | 1.26E-11 |
| Arf6 signaling events | 74 | 1.26E-11 |
| Class I PI3K signaling events | 74 | 1.26E-11 |
| Insulin Pathway | 74 | 1.26E-11 |
| Nectin adhesion pathway | 75 | 1.26E-11 |
| LKB1 signaling events | 75 | 1.26E-11 |
| Thrombin/protease-activated receptor (PAR) pathway | 75 | 1.26E-11 |
| PDGFR-beta signaling pathway | 74 | 1.26E-11 |
| Urokinase-type plasminogen activator (uPA) and uPAR-mediated signaling | 74 | 1.26E-11 |
| ErbB receptor signaling network | 75 | 1.26E-11 |
| EGF receptor (ErbB1) signaling pathway | 74 | 1.26E-11 |
| Class I PI3K signaling events mediated by Akt | 74 | 1.26E-11 |
| ErbB1 downstream signaling | 74 | 1.26E-11 |
| Arf6 downstream pathway | 74 | 1.26E-11 |
| Internalization of ErbB1 | 74 | 1.26E-11 |
| mTOR signaling pathway | 74 | 1.26E-11 |
| EGFR-dependent Endothelin signaling events | 74 | 1.28E-11 |
| IL5-mediated signaling events | 74 | 1.31E-11 |
| GMCSF-mediated signaling events | 74 | 1.31E-11 |
| PDGF receptor signaling network | 74 | 1.31E-11 |
| IGF1 pathway | 74 | 1.31E-11 |
| Signaling events mediated by Hepatocyte Growth Factor Receptor (c-Met) | 74 | 1.31E-11 |
| IL3-mediated signaling events | 74 | 1.37E-11 |
| IFN-gamma pathway | 74 | 1.39E-11 |
| Alpha9 beta1 integrin signaling events | 74 | 1.91E-11 |
| Glypican pathway | 75 | 2.27E-11 |
| Proteoglycan syndecan-mediated signaling events | 75 | 2.87E-11 |
| Integrin family cell surface interactions | 76 | 3.36E-11 |
| Beta1 integrin cell surface interactions | 75 | 3.41E-11 |
| Signal transduction by L1 | 12 | 1.47E-10 |
| Hemostasis | 33 | 1.01E-09 |
| EGFR downregulation | 10 | 3.05E-09 |
| L1CAM interactions | 15 | 4.42E-09 |
| Platelet activation, signaling and aggregation | 19 | 6.63E-09 |
| Signaling by EGFR | 16 | 1.15E-08 |
| Acetylcholine Neurotransmitter Release Cycle | 7 | 1.18E-08 |
| Neurotransmitter Receptor Binding And Downstream Transmission In The Postsynaptic Cell | 15 | 2.32E-08 |
| Axon guidance | 23 | 2.32E-08 |
| Diabetes pathways | 22 | 9.77E-08 |
| Serotonin Neurotransmitter Release Cycle | 6 | 1.20E-07 |
| Dopamine Neurotransmitter Release Cycle | 6 | 1.20E-07 |
| Recycling pathway of L1 | 9 | 3.25E-07 |
| Host Interactions of HIV factors | 17 | 3.82E-07 |
| Regulation of CDC42 activity | 44 | 5.33E-07 |
| Norepinephrine Neurotransmitter Release Cycle | 6 | 5.78E-07 |
| Retrograde neurotrophin signalling | 6 | 5.78E-07 |
| Glutamate Neurotransmitter Release Cycle | 6 | 5.78E-07 |
| CDC42 signaling events | 43 | 8.46E-07 |
| Nef Mediated CD8 Down-regulation | 5 | 1.16E-06 |
| Developmental Biology | 30 | 1.25E-06 |
| GABA B receptor activation | 8 | 1.41E-06 |
| Protein folding | 8 | 1.41E-06 |
| Activation of GABAB receptors | 8 | 1.41E-06 |
| Response to elevated platelet cytosolic Ca2+ | 9 | 1.77E-06 |
| Opioid Signalling | 10 | 2.81E-06 |
| Metabolism of amino acids and derivatives | 18 | 3.90E-06 |
| Integration of energy metabolism | 12 | 3.94E-06 |
| Effects of Botulinum toxin | 5 | 5.85E-06 |
| Nef Mediated CD4 Down-regulation | 5 | 5.85E-06 |
| Metabolism of proteins | 21 | 7.24E-06 |
| GABA receptor activation | 8 | 1.05E-05 |
| Reduction of cytosolic Ca++ levels | 5 | 1.11E-05 |
| Trk receptor signaling mediated by PI3K and PLC-gamma | 10 | 1.35E-05 |
| TNF receptor signaling pathway | 22 | 1.81E-05 |
| Insulin Synthesis and Processing | 14 | 2.09E-05 |
| Signaling by Aurora kinases | 12 | 2.14E-05 |
| Ion transport by P-type ATPases | 7 | 2.46E-05 |
| Aurora B signaling | 8 | 2.74E-05 |
| Gluconeogenesis | 6 | 3.04E-05 |
| Nef-mediates down modulation of cell surface receptors by recruiting them to clathrin adapters | 6 | 4.12E-05 |
| HIV Infection | 17 | 4.46E-05 |
| Signalling by NGF | 14 | 4.53E-05 |
| NGF signalling via TRKA from the plasma membrane | 12 | 4.99E-05 |
| Alpha-synuclein signaling | 7 | 5.74E-05 |
| S1P4 pathway | 5 | 7.02E-05 |
| Metabolism of mRNA | 18 | 7.28E-05 |
| Gene Expression | 24 | 7.28E-05 |
| Vpr-mediated induction of apoptosis by mitochondrial outer membrane permeabilization | 3 | 7.37E-05 |
| Metabolism of RNA | 20 | 7.46E-05 |
| AP-1 transcription factor network | 33 | 7.83E-05 |
| Alpha6Beta4Integrin | 8 | 8.14E-05 |
| Integrin-linked kinase signaling | 34 | 8.93E-05 |
| G protein gated Potassium channels | 5 | 9.22E-05 |
| Inhibition of voltage gated Ca2+ channels via Gbeta/gamma subunits | 5 | 9.22E-05 |
| Formation of ATP by chemiosmotic coupling | 5 | 9.22E-05 |
| Activation of G protein gated Potassium channels | 5 | 9.22E-05 |
| Neurotrophic factor-mediated Trk receptor signaling | 11 | 1.00E-04 |
| LPA receptor mediated events | 11 | 1.00E-04 |
| Platelet calcium homeostasis | 5 | 1.00E-04 |
| Translation | 12 | 1.00E-04 |
| Signaling mediated by p38-alpha and p38-beta | 8 | 1.00E-04 |
| Nongenotropic Androgen signaling | 6 | 1.00E-04 |
| S1P2 pathway | 6 | 1.00E-04 |
| Signaling events mediated by HDAC Class II | 7 | 1.00E-04 |
| PLC beta mediated events | 7 | 1.00E-04 |
| C-MYC pathway | 13 | 2.00E-04 |
| Regulation of mRNA Stability by Proteins that Bind AU-rich Elements | 11 | 2.00E-04 |
| CXCR4-mediated signaling events | 15 | 2.00E-04 |
| Destabilization of mRNA by AUF1 (hnRNP D0) | 9 | 2.00E-04 |
| G-protein mediated events | 7 | 2.00E-04 |
| Platelet degranulation | 6 | 2.00E-04 |
| Folding of actin by CCT/TriC | 4 | 2.00E-04 |
| Eukaryotic Translation Elongation | 10 | 2.00E-04 |
| Platelet homeostasis | 7 | 2.00E-04 |
| The role of Nef in HIV-1 replication and disease pathogenesis | 6 | 2.00E-04 |
| G alpha (s) signalling events | 6 | 3.00E-04 |
| Posttranslational regulation of adherens junction stability and dissassembly | 16 | 4.00E-04 |
| Unfolded Protein Response | 8 | 4.00E-04 |
| p38 signaling mediated by MAPKAP kinases | 5 | 4.00E-04 |
| Ion channel transport | 7 | 4.00E-04 |
| Glucagon signaling in metabolic regulation | 6 | 4.00E-04 |
| Post-chaperonin tubulin folding pathway | 4 | 4.00E-04 |
| Influenza Infection | 12 | 4.00E-04 |
| aspartate degradation II | 3 | 4.00E-04 |
| IL1-mediated signaling events | 16 | 4.00E-04 |
| N-cadherin signaling events | 17 | 4.00E-04 |
| p75(NTR)-mediated signaling | 14 | 4.00E-04 |
| Botulinum neurotoxicity | 3 | 4.00E-04 |
| Semaphorin interactions | 8 | 4.00E-04 |
| Validated targets of C-MYC transcriptional activation | 9 | 4.00E-04 |
| ADP signalling through P2Y purinoceptor 12 | 3 | 8.00E-04 |
| G-protein activation | 4 | 8.00E-04 |
| Inwardly rectifying K+ channels | 5 | 8.00E-04 |
| CXCR3-mediated signaling events | 6 | 8.00E-04 |
| Membrane Trafficking | 9 | 8.00E-04 |
| Glucose metabolism | 6 | 8.00E-04 |
| Stabilization and expansion of the E-cadherin adherens junction | 17 | 1.10E-03 |
| Cooperation of Prefoldin and TriC/CCT in actin and tubulin folding | 4 | 1.10E-03 |
| FAS (CD95) signaling pathway | 11 | 1.10E-03 |
| E-cadherin signaling in the nascent adherens junction | 17 | 1.10E-03 |
| BMP receptor signaling | 15 | 1.10E-03 |
| IL8- and CXCR1-mediated signaling events | 5 | 1.40E-03 |
| Trafficking of GluR2-containing AMPA receptors | 3 | 1.40E-03 |
| E-cadherin signaling events | 17 | 1.40E-03 |
| Influenza Life Cycle | 11 | 1.40E-03 |
| Apoptosis | 12 | 1.40E-03 |
| Chaperonin-mediated protein folding | 4 | 1.40E-03 |
| EGFR1 | 11 | 1.40E-03 |
| Signal amplification | 4 | 1.40E-03 |
| sucrose degradation | 3 | 1.40E-03 |
| S1P3 pathway | 5 | 1.70E-03 |
| Insulin-mediated glucose transport | 5 | 1.70E-03 |
| Mitotic Prophase | 7 | 2.00E-03 |
| methylglyoxal degradation I | 2 | 2.00E-03 |
| Loss of Nlp from mitotic centrosomes | 7 | 2.00E-03 |
| p38 MAPK signaling pathway | 13 | 2.00E-03 |
| Activation of Chaperones by IRE1alpha | 6 | 2.00E-03 |
| Golgi Cisternae Pericentriolar Stack Reorganization | 7 | 2.00E-03 |
| Loss of proteins required for interphase microtubule organizationÃ‚Â from the centrosome | 7 | 2.00E-03 |
| Pyruvate metabolism and Citric Acid (TCA) cycle | 5 | 2.20E-03 |
| L13a-mediated translational silencing of Ceruloplasmin expression | 9 | 2.20E-03 |
| Formation of tubulin folding intermediates by CCT/TriC | 3 | 2.20E-03 |
| S1P5 pathway | 3 | 2.20E-03 |
| Disinhibition of SNARE formation | 3 | 2.20E-03 |
| 3' -UTR-mediated translational regulation | 9 | 2.20E-03 |
| Peptide chain elongation | 8 | 2.50E-03 |
| GTP hydrolysis and joining of the 60S ribosomal subunit | 9 | 2.50E-03 |
| fatty acid beta-oxidation I | 4 | 2.80E-03 |
| Association of TriC/CCT with target proteins during biosynthesis | 3 | 3.10E-03 |
| IL8- and CXCR2-mediated signaling events | 5 | 3.40E-03 |
| Glutamate Binding, Activation of AMPA Receptors and Synaptic Plasticity | 4 | 3.80E-03 |
| Cap-dependent Translation Initiation | 9 | 3.80E-03 |
| Centrosome maturation | 7 | 3.80E-03 |
| Arf1 pathway | 4 | 3.80E-03 |
| Recruitment of mitotic centrosome proteins and complexes | 7 | 3.80E-03 |
| G alpha (12/13) signalling events | 4 | 3.80E-03 |
| Trafficking of AMPA receptors | 4 | 3.80E-03 |
| Eukaryotic Translation Initiation | 9 | 3.80E-03 |
| Amino acid synthesis and interconversion (transamination) | 3 | 4.10E-03 |
| Metabolism of carbohydrates | 8 | 4.40E-03 |
| aspartate biosynthesis | 2 | 4.50E-03 |
| Utilization of Ketone Bodies | 2 | 4.50E-03 |
| Formation of a pool of free 40S subunits | 8 | 4.50E-03 |
| Proteolytic cleavage of SNARE complex proteins | 2 | 4.50E-03 |
| glutamate degradation II | 2 | 4.50E-03 |
| G alpha (z) signalling events | 4 | 4.50E-03 |
| Signaling by FGFR | 8 | 5.00E-03 |
| Transmembrane transport of small molecules | 19 | 5.00E-03 |
| Regulation of p38-alpha and p38-beta | 11 | 5.00E-03 |
| Mitotic M-M/G1 phases | 14 | 5.20E-03 |
| isoleucine degradation I | 3 | 5.20E-03 |
| IL8-mediated signaling events | 5 | 5.20E-03 |
| Apoptotic cleavage of cellular proteins | 5 | 5.70E-03 |
| Syndecan-2-mediated signaling events | 7 | 6.00E-03 |
| Hormone-sensitive lipase (HSL)-mediated triacylglycerol hydrolysis | 3 | 6.40E-03 |
| Influenza Viral RNA Transcription and Replication | 8 | 6.40E-03 |
| Interaction between L1 and Ankyrins | 3 | 6.40E-03 |
| valine degradation I | 3 | 6.40E-03 |
| Regulation of nuclear SMAD2/3 signaling | 16 | 6.50E-03 |
| TNF alpha/NF-kB | 11 | 6.50E-03 |
| Regulation of cytoplasmic and nuclear SMAD2/3 signaling | 16 | 6.50E-03 |
| TGF-beta receptor signaling | 16 | 6.50E-03 |
| ER-Phagosome pathway | 7 | 7.20E-03 |
| Hypoxic and oxygen homeostasis regulation of HIF-1-alpha | 7 | 7.20E-03 |
| Autodegradation of Cdh1 by Cdh1:APC/C | 6 | 7.20E-03 |
| glutamate degradation III (via 4-aminobutyrate) | 2 | 7.90E-03 |
| Inhibition of adenylate cyclase pathway | 3 | 7.90E-03 |
| Catecholamine biosynthesis | 2 | 7.90E-03 |
| Adenylate cyclase inhibitory pathway | 3 | 7.90E-03 |
| Breakdown of the nuclear lamina | 2 | 7.90E-03 |
| heme degradation | 2 | 7.90E-03 |
| Reuptake of GABA | 2 | 7.90E-03 |
| TCA cycle variation III (eukaryotic) | 3 | 7.90E-03 |
| catecholamine biosynthesis | 2 | 7.90E-03 |
| Fatty acid, triacylglycerol, and ketone body metabolism | 7 | 8.20E-03 |
| Nonsense-Mediated Decay | 8 | 8.20E-03 |
| Viral mRNA Translation | 7 | 8.20E-03 |
| Eukaryotic Translation Termination | 7 | 8.20E-03 |
| Nonsense Mediated Decay Enhanced by the Exon Junction Complex | 8 | 8.20E-03 |
| RAC1 signaling pathway | 12 | 8.30E-03 |
| Regulation of RhoA activity | 12 | 8.30E-03 |
| Regulation of RAC1 activity | 12 | 8.30E-03 |
| Ca-dependent events | 4 | 8.30E-03 |
| RhoA signaling pathway | 12 | 8.30E-03 |
| DNA Replication | 14 | 8.90E-03 |
| mRNA Splicing | 8 | 8.90E-03 |
| mRNA Splicing - Major Pathway | 8 | 8.90E-03 |
| Prefoldin mediated transfer of substrate to CCT/TriC | 3 | 9.10E-03 |
| Endosomal Sorting Complex Required For Transport (ESCRT) | 4 | 9.20E-03 |
| Signaling events mediated by the Hedgehog family | 6 | 9.60E-03 |
| Antigen processing-Cross presentation | 7 | 9.60E-03 |
| ALK1 signaling events | 16 | 9.60E-03 |
| Adaptive Immune System | 13 | 9.80E-03 |

**Table 9**: Subnetwork clustering analysis of the D2 PPI network of the META list. Analysis performed using the GO Consortium platform (http://geneontology.org/).

| **Cluster** | **Biological Process** | **Molecular Function** | **Cellular component** |
| --- | --- | --- | --- |
| **CYC1, ERCC8, HSPA1L, MACF1, MARCKS, OLA1, OPTN, PLXND1, PRMT5, PSMA2, PSMA3, PSMA5, SGK1, SIP1, SIRT3, SNRPD1, SNRPF, UQCRC1, UQCRH, UQCRQ** | energy derivation by oxidation of organic compounds (GO:0015980)  cellular respiration (GO:0045333)  cellular metabolic process (GO:0044237)  single-organism metabolic process (GO:0044710)  generation of precursor metabolites and energy (GO:0006091)  respiratory electron transport chain (GO:0022904)  electron transport chain (GO:0022900)  spliceosomal snRNP assembly (GO:0000387)  metabolic process (GO:0008152)  mRNA metabolic process (GO:0016071)  aerobic respiration (GO:0009060)  mitochondrial electron transport, ubiquinol to cytochrome c (GO:0006122)  protein polyubiquitination (GO:0000209)  small molecule metabolic process (GO:0044281)  regulation of cellular amino acid metabolic process (GO:0006521)  proteasome-mediated ubiquitin-dependent protein catabolic process (GO:0043161)  DNA damage response, signal transduction by p53 class mediator resulting in cell cycle arrest (GO:0006977)  proteasomal protein catabolic process (GO:0010498)  signal transduction involved in mitotic G1 DNA damage checkpoint (GO:0072431)  intracellular signal transduction involved in G1 DNA damage checkpoint (GO:1902400)  negative regulation of ubiquitin-protein ligase activity involved in mitotic cell cycle (GO:0051436)  signal transduction involved in DNA damage checkpoint (GO:0072422)  signal transduction involved in mitotic cell cycle checkpoint (GO:0072413)  signal transduction involved in DNA integrity checkpoint (GO:0072401)  signal transduction involved in mitotic DNA integrity checkpoint (GO:1902403)  signal transduction involved in mitotic DNA damage checkpoint (GO:1902402)  signal transduction involved in cell cycle checkpoint (GO:0072395)  negative regulation of ubiquitin-protein transferase activity (GO:0051444)  negative regulation of ligase activity (GO:0051352)  positive regulation of ubiquitin-protein ligase activity involved in mitotic cell cycle (GO:0051437)  antigen processing and presentation of exogenous peptide antigen via MHC class I, TAP-dependent (GO:0002479)  mitotic G1/S transition checkpoint (GO:0044819)  mitotic G1 DNA damage checkpoint (GO:0031571)  G1 DNA damage checkpoint (GO:0044783)  regulation of cellular amine metabolic process (GO:0033238)  regulation of ubiquitin-protein ligase activity involved in mitotic cell cycle (GO:0051439)  antigen processing and presentation of exogenous peptide antigen via MHC class I (GO:0042590)  anaphase-promoting complex-dependent proteasomal ubiquitin-dependent protein catabolic process (GO:0031145)  positive regulation of cell cycle arrest (GO:0071158)  positive regulation of ubiquitin-protein transferase activity (GO:0051443)  positive regulation of ligase activity (GO:0051351)  DNA damage response, signal transduction by p53 class mediator (GO:0030330)  mitotic DNA damage checkpoint (GO:0044773)  cellular process (GO:0009987)  negative regulation of G1/S transition of mitotic cell cycle (GO:2000134)  regulation of ubiquitin-protein transferase activity (GO:0051438)  negative regulation of cell cycle G1/S phase transition (GO:1902807)  mitotic DNA integrity checkpoint (GO:0044774)  antigen processing and presentation of peptide antigen via MHC class I (GO:0002474)  regulation of cell cycle arrest (GO:0071156)  regulation of ligase activity (GO:0051340)  signal transduction in response to DNA damage (GO:0042770)  hydrogen ion transmembrane transport (GO:1902600)  negative regulation of protein ubiquitination (GO:0031397)  mitotic cell cycle phase transition (GO:0044772)  cell cycle phase transition (GO:0044770)  oxidation-reduction process (GO:0055114)  negative regulation of protein modification by small protein conjugation or removal (GO:1903321)  S phase (GO:0051320)  mitotic S phase (GO:0000084)  regulation of G1/S transition of mitotic cell cycle (GO:2000045)  regulation of cell cycle G1/S phase transition (GO:1902806)  cellular response to DNA damage stimulus (GO:0006974)  proton transport (GO:0015992)  hydrogen transport (GO:0006818)  signal transduction by p53 class mediator (GO:0072331)  ribonucleoprotein complex assembly (GO:0022618)  Golgi to plasma membrane protein transport (GO:0043001) | ubiquinol-cytochrome-c reductase activity (GO:0008121)  oxidoreductase activity, acting on diphenols and related substances as donors (GO:0016679)  threonine-type endopeptidase activity (GO:0004298)  threonine-type peptidase activity (GO:0070003)  protein binding (GO:0005515)  hydrogen ion transmembrane transporter activity (GO:0015078)  binding (GO:0005488)  protein complex binding (GO:0032403) | proteasome core complex, alpha-subunit complex (GO:0019773)  methylosome (GO:0034709)  intracellular organelle part (GO:0044446)  organelle part (GO:0044422)  respiratory chain (GO:0070469)  proteasome core complex (GO:0005839)  cytoplasmic part (GO:0044444)  intracellular membrane-bounded organelle (GO:0043231)  pICln-Sm protein complex (GO:0034715)  respiratory chain complex III (GO:0045275)  mitochondrial respiratory chain complex III (GO:0005750)  intracellular organelle lumen (GO:0070013)  proteasome complex (GO:0000502)  U4 snRNP (GO:0005687)  organelle lumen (GO:0043233)  intracellular (GO:0005622)  membrane-enclosed lumen (GO:0031974)  membrane-bounded organelle (GO:0043227)  intracellular organelle (GO:0043229)  methyltransferase complex (GO:0034708)  mitochondrial part (GO:0044429)  cytoplasm (GO:0005737)  catalytic complex (GO:1902494)  U1 snRNP (GO:0005685)  protein complex (GO:0043234)  nuclear part (GO:0044428)  SMN-Sm protein complex (GO:0034719)  cytochrome complex (GO:0070069)  nucleoplasm (GO:0005654)  organelle (GO:0043226)  cytosol (GO:0005829)  U12-type spliceosomal complex (GO:0005689)  mitochondrion (GO:0005739)  nuclear lumen (GO:0031981)  mitochondrial inner membrane (GO:0005743) |
| **AHSG, AKR1B1, BCAS1, CAMKK2, CDC42, CEP63, DYNLL1, ELF3, ETFA, FTSJ1, FUBP1, GDI1, GSTM3, HIST1H2AB, MPG, MSN, RBL1, RUVBL2, SERBP1, SMN1, SNCG, SNRNP70, SRCAP, TFE3, TSPAN7, VIM** | epithelial cell development (GO:0002064)  positive regulation of gene expression (GO:0010628)  organic cyclic compound metabolic process (GO:1901360)  epithelial cell differentiation (GO:0030855) | protein binding (GO:0005515) | extracellular organelle (GO:0043230)  extracellular vesicular exosome (GO:0070062)  extracellular membrane-bounded organelle (GO:0065010)  intracellular non-membrane-bounded organelle (GO:0043232)  non-membrane-bounded organelle (GO:0043228)  organelle (GO:0043226)  membrane-bounded organelle (GO:0043227)  intracellular organelle (GO:0043229)  membrane-bounded vesicle (GO:0031988)  intracellular part (GO:0044424)  vesicle (GO:0031982)  intracellular (GO:0005622)  extracellular region part (GO:0044421)  spindle (GO:0005819) |
| **ACTR6, CS, DHRS2, EEF1A2, EEF1B2, HSPB1, MAP3K8, PFKL, PHB2, PTPLAD1, SERINC3, TAB2, TAGLN2, TUBA3D, USP1, VDAC2** | regulation of intrinsic apoptotic signaling pathway (GO:2001242)  regulation of cellular response to stress (GO:0080135)  regulation of signaling (GO:0023051)  regulation of cell communication (GO:0010646)  regulation of intracellular signal transduction (GO:1902531)  regulation of response to stress (GO:0080134)  regulation of response to stimulus (GO:0048583)  MAPK cascade (GO:0000165)  regulation of signal transduction (GO:0009966)  signal transduction by phosphorylation (GO:0023014)  activation of protein kinase activity (GO:0032147)  I-kappaB kinase/NF-kappaB signaling (GO:0007249)  positive regulation of endoplasmic reticulum stress-induced intrinsic apoptotic signaling pathway (GO:1902237) | translation elongation factor activity (GO:0003746)  citrate synthase activity (GO:0036440)  citrate (Si)-synthase activity (GO:0004108) | eukaryotic translation elongation factor 1 complex (GO:0005853)  cytoplasmic part (GO:0044444)  extracellular organelle (GO:0043230)  extracellular vesicular exosome (GO:0070062)  extracellular membrane-bounded organelle (GO:0065010) |
| **AP2A1, AP2A2, AP2S1, ARHGEF7, ATP2B2, CCDC50, CUL3, EPS15, GABBR2, GRHPR, HSP90AA1, LASS2, LSM1, NARS, OTUD7B, PRKCD, RPS3A, SHC1, SLC9A3R2, TNFRSF10A, TOE1** | regulation of epidermal growth factor receptor signaling pathway (GO:0042058)  regulation of ERBB signaling pathway (GO:1901184)  epidermal growth factor receptor signaling pathway (GO:0007173)  ERBB signaling pathway (GO:0038127)  negative regulation of epidermal growth factor receptor signaling pathway (GO:0042059)  negative regulation of ERBB signaling pathway (GO:1901185)  negative regulation of signal transduction (GO:0009968)  negative regulation of signaling (GO:0023057)  negative regulation of cell communication (GO:0010648)  negative regulation of response to stimulus (GO:0048585)  neurotrophin TRK receptor signaling pathway (GO:0048011)  neurotrophin signaling pathway (GO:0038179)  regulation of signaling (GO:0023051)  regulation of cell communication (GO:0010646)  transmembrane receptor protein tyrosine kinase signaling pathway (GO:0007169)  regulation of signal transduction (GO:0009966)  endocytosis (GO:0006897)  regulation of response to stimulus (GO:0048583)  regulation of defense response to virus by virus (GO:0050690)  enzyme linked receptor protein signaling pathway (GO:0007167)  cell surface receptor signaling pathway (GO:0007166)  vesicle coating (GO:0006901)  cellular response to growth factor stimulus (GO:0071363)  response to growth factor (GO:0070848)  cellular component organization (GO:0016043)  membrane budding (GO:0006900)  cell morphogenesis involved in differentiation (GO:0000904)  cellular component organization or biogenesis (GO:0071840)  vesicle-mediated transport (GO:0016192)  locomotion (GO:0040011)  negative regulation of cellular process (GO:0048523)  intracellular transport (GO:0046907)  regulation of defense response to virus (GO:0050688)  protein complex subunit organization (GO:0071822)  cell morphogenesis (GO:0000902)  clathrin coat assembly (GO:0048268)  cell morphogenesis involved in neuron differentiation (GO:0048667)  Golgi to endosome transport (GO:0006895)  negative regulation of biological process (GO:0048519)  antigen processing and presentation of exogenous peptide antigen via MHC class II (GO:0019886)  antigen processing and presentation of peptide antigen via MHC class II (GO:0002495)  antigen processing and presentation of peptide or polysaccharide antigen via MHC class II (GO:0002504)  protein complex assembly (GO:0006461)  protein complex biogenesis (GO:0070271)  cell projection organization (GO:0030030)  cellular component morphogenesis (GO:0032989)  regulation of response to biotic stimulus (GO:0002831)  regulation of intracellular signal transduction (GO:1902531)  vesicle organization (GO:0016050)  intracellular protein transport (GO:0006886)  negative regulation of intracellular signal transduction (GO:1902532)  cytoplasmic transport (GO:0016482)  macromolecular complex assembly (GO:0065003)  cellular component assembly (GO:0022607)  establishment of localization in cell (GO:0051649) | protein binding (GO:0005515)  protein transporter activity (GO:0008565)  enzyme binding (GO:0019899)  binding (GO:0005488)  protein C-terminus binding (GO:0008022)  polyubiquitin binding (GO:0031593) | clathrin adaptor complex (GO:0030131)  AP-2 adaptor complex (GO:0030122)  clathrin-coated endocytic vesicle membrane (GO:0030669)  AP-type membrane coat adaptor complex (GO:0030119)  clathrin coat of endocytic vesicle (GO:0030128)  clathrin-coated endocytic vesicle (GO:0045334)  clathrin coat (GO:0030118)  clathrin coat of coated pit (GO:0030132)  coated pit (GO:0005905)  cytosol (GO:0005829)  clathrin-coated vesicle membrane (GO:0030665)  clathrin vesicle coat (GO:0030125)  membrane coat (GO:0030117)  coated membrane (GO:0048475)  endocytic vesicle (GO:0030139)  coated vesicle membrane (GO:0030662)  clathrin-coated vesicle (GO:0030136)  endocytic vesicle membrane (GO:0030666)  vesicle coat (GO:0030120)  coated vesicle (GO:0030135)  membrane-bounded vesicle (GO:0031988)  cytoplasm (GO:0005737)  vesicle (GO:0031982)  cytoplasmic vesicle part (GO:0044433)  intracellular part (GO:0044424)  intracellular (GO:0005622)  cytoplasmic vesicle membrane (GO:0030659)  vesicle membrane (GO:0012506) |
| **ACTBL2, ARRB2, CHEK2, CLTB, CRMP1, DLG5, DLST, HNRNPH1, HNRNPH2, NDRG1, NSUN2, PFN1, PKM, PPP2CB, PPP2R2A, PPP2R5B, PPP2R5C, PPP2R5D, PPP2R5E, RPS12, SAG, SGOL2, TOB1** | organic substance catabolic process (GO:1901575)  catabolic process (GO:0009056)  cellular macromolecule catabolic process (GO:0044265)  signal transduction in response to DNA damage (GO:0042770)  macromolecule catabolic process (GO:0009057)  cell death (GO:0008219)  death (GO:0016265)  cellular catabolic process (GO:0044248) | protein phosphatase type 2A regulator activity (GO:0008601)  protein phosphatase regulator activity (GO:0019888)  phosphatase regulator activity (GO:0019208)  enzyme regulator activity (GO:0030234)  protein serine/threonine phosphatase activity (GO:0004722)  binding (GO:0005488)  protein binding (GO:0005515)  molecular_function (GO:0003674) | protein phosphatase type 2A complex (GO:0000159)  protein serine/threonine phosphatase complex (GO:0008287)  phosphatase complex (GO:1903293)  intracellular part (GO:0044424)  cytosol (GO:0005829)  intracellular (GO:0005622)  catalytic complex (GO:1902494)  macromolecular complex (GO:0032991)  chromosomal region (GO:0098687)  cell part (GO:0044464)  cell (GO:0005623)  cytoplasm (GO:0005737)  nucleus (GO:0005634) |
| **ACTR3B, ARHGEF1, ARPC5, ARPC5L, ATP5A1, ATP5B, ATP5C1, ATP5F1, ATP5H, CCT2, CCT7, DDT, DSTYK, EIF3G, EIF3H, GNA13, MTPN, NGEF, PPP2R2D, PRAF2, RHOB, USP3** | mitochondrial ATP synthesis coupled proton transport (GO:0042776)  ATP synthesis coupled proton transport (GO:0015986)  energy coupled proton transport, down electrochemical gradient (GO:0015985)  ATP biosynthetic process (GO:0006754)  purine ribonucleoside triphosphate biosynthetic process (GO:0009206)  purine nucleoside triphosphate biosynthetic process (GO:0009145)  ribonucleoside triphosphate biosynthetic process (GO:0009201)  purine ribonucleoside monophosphate biosynthetic process (GO:0009168)  purine nucleoside monophosphate biosynthetic process (GO:0009127)  nucleoside triphosphate biosynthetic process (GO:0009142)  ribonucleoside monophosphate biosynthetic process (GO:0009156)  nucleoside monophosphate biosynthetic process (GO:0009124)  purine ribonucleoside biosynthetic process (GO:0046129)  purine nucleoside biosynthetic process (GO:0042451)  hydrogen ion transmembrane transport (GO:1902600)  ribonucleoside biosynthetic process (GO:0042455)  respiratory electron transport chain (GO:0022904)  electron transport chain (GO:0022900)  nucleoside biosynthetic process (GO:0009163)  glycosyl compound biosynthetic process (GO:1901659)  purine ribonucleotide biosynthetic process (GO:0009152)  purine nucleotide biosynthetic process (GO:0006164)  proton transport (GO:0015992)  hydrogen transport (GO:0006818)  ribonucleotide biosynthetic process (GO:0009260)  mitochondrial transport (GO:0006839)  ribose phosphate biosynthetic process (GO:0046390)  purine-containing compound biosynthetic process (GO:0072522)  purine ribonucleoside triphosphate catabolic process (GO:0009207)  ribonucleoside triphosphate catabolic process (GO:0009203)  purine nucleoside triphosphate catabolic process (GO:0009146)  cellular respiration (GO:0045333)  nucleoside triphosphate catabolic process (GO:0009143)  purine ribonucleoside catabolic process (GO:0046130)  purine nucleoside catabolic process (GO:0006152)  ribonucleoside catabolic process (GO:0042454)  purine ribonucleotide catabolic process (GO:0009154)  ribonucleotide catabolic process (GO:0009261)  nucleoside catabolic process (GO:0009164)  purine nucleotide catabolic process (GO:0006195)  glycosyl compound catabolic process (GO:1901658)  purine-containing compound catabolic process (GO:0072523)  nucleotide catabolic process (GO:0009166)  nucleoside phosphate catabolic process (GO:1901292)  nucleotide biosynthetic process (GO:0009165)  nucleoside phosphate biosynthetic process (GO:1901293)  organophosphate catabolic process (GO:0046434)  purine ribonucleoside triphosphate metabolic process (GO:0009205)  ribonucleoside triphosphate metabolic process (GO:0009199)  purine nucleoside triphosphate metabolic process (GO:0009144)  nucleoside triphosphate metabolic process (GO:0009141)  carbohydrate derivative catabolic process (GO:1901136)  purine ribonucleoside metabolic process (GO:0046128)  purine nucleoside metabolic process (GO:0042278)  ribonucleoside metabolic process (GO:0009119)  purine ribonucleotide metabolic process (GO:0009150)  nucleoside metabolic process (GO:0009116)  ribonucleotide metabolic process (GO:0009259)  ribose phosphate metabolic process (GO:0019693)  purine nucleotide metabolic process (GO:0006163)  glycosyl compound metabolic process (GO:1901657)  ATP catabolic process (GO:0006200)  purine ribonucleoside monophosphate catabolic process (GO:0009169)  ribonucleoside monophosphate catabolic process (GO:0009158)  purine nucleoside monophosphate catabolic process (GO:0009128)  purine-containing compound metabolic process (GO:0072521)  nucleoside monophosphate catabolic process (GO:0009125)  organonitrogen compound catabolic process (GO:1901565)  energy derivation by oxidation of organic compounds (GO:0015980)  Rho protein signal transduction (GO:0007266)  nucleobase-containing compound catabolic process (GO:0034655)  nucleotide metabolic process (GO:0009117)  nucleoside phosphate metabolic process (GO:0006753)  cellular nitrogen compound catabolic process (GO:0044270)  aromatic compound catabolic process (GO:0019439)  heterocycle catabolic process (GO:0046700)  organic cyclic compound catabolic process (GO:1901361)  nucleobase-containing small molecule metabolic process (GO:0055086)  monovalent inorganic cation transport (GO:0015672)  ATP metabolic process (GO:0046034)  generation of precursor metabolites and energy (GO:0006091)  purine ribonucleoside monophosphate metabolic process (GO:0009167)  purine nucleoside monophosphate metabolic process (GO:0009126)  ribonucleoside monophosphate metabolic process (GO:0009161)  inorganic cation transmembrane transport (GO:0098662)  organophosphate biosynthetic process (GO:0090407)  nucleoside monophosphate metabolic process (GO:0009123)  cation transmembrane transport (GO:0098655)  inorganic ion transmembrane transport (GO:0098660)  organophosphate metabolic process (GO:0019637)  formation of translation preinitiation complex (GO:0001731)  single-organism catabolic process (GO:0044712)  cellular catabolic process (GO:0044248)  carbohydrate derivative biosynthetic process (GO:1901137)  organonitrogen compound metabolic process (GO:1901564)  organonitrogen compound biosynthetic process (GO:1901566) | proton-transporting ATP synthase activity, rotational mechanism (GO:0046933)  ATPase activity, coupled to transmembrane movement of ions, rotational mechanism (GO:0044769)  hydrogen ion transmembrane transporter activity (GO:0015078)  cation-transporting ATPase activity (GO:0019829)  ATPase activity, coupled to transmembrane movement of ions (GO:0042625)  proton-transporting ATPase activity, rotational mechanism (GO:0046961)  hydrogen-exporting ATPase activity (GO:0036442)  ATPase activity, coupled to transmembrane movement of substances (GO:0042626)  ATPase activity, coupled to movement of substances (GO:0043492)  hydrolase activity, acting on acid anhydrides, catalyzing transmembrane movement of substances (GO:0016820)  P-P-bond-hydrolysis-driven transmembrane transporter activity (GO:0015405)  primary active transmembrane transporter activity (GO:0015399)  nucleoside-triphosphatase activity (GO:0017111)  pyrophosphatase activity (GO:0016462)  hydrolase activity, acting on acid anhydrides, in phosphorus-containing anhydrides (GO:0016818)  hydrolase activity, acting on acid anhydrides (GO:0016817)  monovalent inorganic cation transmembrane transporter activity (GO:0015077)  ATPase activity (GO:0016887)  MHC class I protein binding (GO:0042288)  inorganic cation transmembrane transporter activity (GO:0022890)  ATPase activity, coupled (GO:0042623)  nucleotide binding (GO:0000166)  nucleoside phosphate binding (GO:1901265)  purine ribonucleoside triphosphate binding (GO:0035639)  MHC protein binding (GO:0042287)  purine ribonucleoside binding (GO:0032550)  purine nucleoside binding (GO:0001883)  ribonucleoside binding (GO:0032549)  active transmembrane transporter activity (GO:0022804)  nucleoside binding (GO:0001882)  purine ribonucleotide binding (GO:0032555)  ribonucleotide binding (GO:0032553)  purine nucleotide binding (GO:0017076)  cation transmembrane transporter activity (GO:0008324) | mitochondrial proton-transporting ATP synthase complex (GO:0005753)  proton-transporting ATP synthase complex (GO:0045259)  proton-transporting two-sector ATPase complex (GO:0016469)  extracellular organelle (GO:0043230)  extracellular vesicular exosome (GO:0070062)  extracellular membrane-bounded organelle (GO:0065010)  proton-transporting ATP synthase complex, catalytic core F(1) (GO:0045261)  membrane-bounded vesicle (GO:0031988)  vesicle (GO:0031982)  extracellular region part (GO:0044421)  cytoplasm (GO:0005737)  mitochondrial membrane part (GO:0044455)  proton-transporting two-sector ATPase complex, catalytic domain (GO:0033178)  extracellular region (GO:0005576)  intracellular part (GO:0044424)  intracellular (GO:0005622)  macromolecular complex (GO:0032991)  protein complex (GO:0043234)  mitochondrial proton-transporting ATP synthase complex, catalytic core F(1) (GO:0000275)  mitochondrial proton-transporting ATP synthase complex, coupling factor F(o) (GO:0000276)  mitochondrial inner membrane (GO:0005743)  chaperonin-containing T-complex (GO:0005832)  organelle inner membrane (GO:0019866)  zona pellucida receptor complex (GO:0002199)  proton-transporting ATP synthase complex, coupling factor F(o) (GO:0045263)  eukaryotic 48S preinitiation complex (GO:0033290)  eukaryotic 43S preinitiation complex (GO:0016282)  translation preinitiation complex (GO:0070993)  eukaryotic translation initiation factor 3 complex (GO:0005852)  cytoplasmic part (GO:0044444)  mitochondrial membrane (GO:0031966)  cell part (GO:0044464)  cell (GO:0005623)  mitochondrial envelope (GO:0005740)  proton-transporting two-sector ATPase complex, proton-transporting domain (GO:0033177)  mitochondrial matrix (GO:0005759) |
| **ATP12A, C21ORF33, CAMK2A, CAMK2B, CHMP5, CLTA, CPLX1, CPSF2, CTNNB1, EEF1D, FRA10AC1, GLRX3, HSP90B1, INPP5D, MME, NDUFB4, PIP4K2B, RBM39, RNPS1, RXRB, STAMBP** | regulation of biological quality (GO:0065008)  negative regulation of osteoclast differentiation (GO:0045671)  biological regulation (GO:0065007) | protein binding (GO:0005515)  glutamate receptor binding (GO:0035254)  calmodulin-dependent protein kinase activity (GO:0004683) | cytosol (GO:0005829)  endocytic vesicle (GO:0030139)  cytoplasmic part (GO:0044444)  intracellular organelle lumen (GO:0070013)  nuclear lumen (GO:0031981)  organelle lumen (GO:0043233)  membrane-enclosed lumen (GO:0031974)  intracellular part (GO:0044424)  intracellular (GO:0005622)  endocytic vesicle membrane (GO:0030666)  membrane-bounded vesicle (GO:0031988)  nuclear part (GO:0044428)  nucleoplasm (GO:0005654)  cell part (GO:0044464)  cell (GO:0005623)  vesicle (GO:0031982)  organelle part (GO:0044422)  nucleus (GO:0005634) |
| **ACHE, ACTB, ALDOA, DDX24, DPYSL5, E2F1, ENO1, FHIT, GNAI2, GNB2L1, HSP90AB1, HSPA5, HSPD1, LAP3, MDH2, MRPL10, MYH9, NDUFA13, RPSA, STARD13, TPI1, TRRAP, TUBA1B, TUBA4A, USP22, VDAC3, XRN1** | nucleobase-containing small molecule metabolic process (GO:0055086)  organic substance catabolic process (GO:1901575)  catabolic process (GO:0009056)  nucleotide metabolic process (GO:0009117)  nucleoside phosphate metabolic process (GO:0006753)  organophosphate metabolic process (GO:0019637)  single-organism catabolic process (GO:0044712)  organonitrogen compound metabolic process (GO:1901564)  nucleobase-containing compound metabolic process (GO:0006139)  small molecule metabolic process (GO:0044281)  cellular nitrogen compound catabolic process (GO:0044270)  heterocycle catabolic process (GO:0046700)  heterocycle metabolic process (GO:0046483)  cellular aromatic compound metabolic process (GO:0006725)  primary metabolic process (GO:0044238)  organic cyclic compound catabolic process (GO:1901361)  metabolic process (GO:0008152)  organic substance metabolic process (GO:0071704)  purine nucleotide metabolic process (GO:0006163)  cellular nitrogen compound metabolic process (GO:0034641)  organic cyclic compound metabolic process (GO:1901360)  purine-containing compound metabolic process (GO:0072521)  gluconeogenesis (GO:0006094)  cellular catabolic process (GO:0044248)  hexose biosynthetic process (GO:0019319)  'de novo' protein folding (GO:0006458)  purine ribonucleoside triphosphate metabolic process (GO:0009205)  nucleobase-containing compound catabolic process (GO:0034655)  cellular metabolic process (GO:0044237)  ribonucleoside triphosphate metabolic process (GO:0009199)  purine nucleoside triphosphate metabolic process (GO:0009144)  nitrogen compound metabolic process (GO:0006807)  monosaccharide biosynthetic process (GO:0046364)  nucleoside triphosphate metabolic process (GO:0009141)  aromatic compound catabolic process (GO:0019439)  single-organism metabolic process (GO:0044710)  purine ribonucleoside metabolic process (GO:0046128)  purine nucleoside metabolic process (GO:0042278)  ribonucleoside metabolic process (GO:0009119)  purine ribonucleotide metabolic process (GO:0009150)  purine ribonucleoside triphosphate catabolic process (GO:0009207)  ribonucleoside triphosphate catabolic process (GO:0009203)  purine nucleoside triphosphate catabolic process (GO:0009146)  nucleoside metabolic process (GO:0009116)  ribonucleotide metabolic process (GO:0009259)  nucleoside triphosphate catabolic process (GO:0009143)  ribose phosphate metabolic process (GO:0019693)  purine ribonucleoside catabolic process (GO:0046130)  purine nucleoside catabolic process (GO:0006152)  glycosyl compound metabolic process (GO:1901657)  ribonucleoside catabolic process (GO:0042454)  purine ribonucleotide catabolic process (GO:0009154)  ribonucleotide catabolic process (GO:0009261)  cellular response to interleukin-4 (GO:0071353)  nucleoside catabolic process (GO:0009164)  purine nucleotide catabolic process (GO:0006195)  glycosyl compound catabolic process (GO:1901658)  purine-containing compound catabolic process (GO:0072523)  nucleotide catabolic process (GO:0009166)  organonitrogen compound catabolic process (GO:1901565)  protein metabolic process (GO:0019538)  nucleoside phosphate catabolic process (GO:1901292)  phosphate-containing compound metabolic process (GO:0006796)  protein folding (GO:0006457)  phosphorus metabolic process (GO:0006793)  response to interleukin-4 (GO:0070670)  macromolecule metabolic process (GO:0043170)  organophosphate catabolic process (GO:0046434)  carbohydrate derivative catabolic process (GO:1901136)  single-organism cellular process (GO:0044763)  carbohydrate biosynthetic process (GO:0016051)  macromolecular complex subunit organization (GO:0043933)  cellular process (GO:0009987)  glycolytic process (GO:0006096)  'de novo' posttranslational protein folding (GO:0051084)  carbohydrate derivative metabolic process (GO:1901135)  glucose metabolic process (GO:0006006)  cell activation (GO:0001775)  hexose metabolic process (GO:0019318)  regulation of proteasomal ubiquitin-dependent protein catabolic process (GO:0032434)  ATP metabolic process (GO:0046034)  generation of precursor metabolites and energy (GO:0006091)  pyruvate metabolic process (GO:0006090)  anatomical structure arrangement (GO:0048532)  platelet activation (GO:0030168)  purine ribonucleoside monophosphate metabolic process (GO:0009167)  purine nucleoside monophosphate metabolic process (GO:0009126)  monosaccharide metabolic process (GO:0005996)  response to wounding (GO:0009611) | poly(A) RNA binding (GO:0044822)  RNA binding (GO:0003723)  hydrolase activity (GO:0016787)  heterocyclic compound binding (GO:1901363)  organic cyclic compound binding (GO:0097159)  catalytic activity (GO:0003824)  pyrophosphatase activity (GO:0016462)  hydrolase activity, acting on acid anhydrides, in phosphorus-containing anhydrides (GO:0016818)  hydrolase activity, acting on acid anhydrides (GO:0016817)  protein binding (GO:0005515)  binding (GO:0005488)  nucleotide binding (GO:0000166)  nucleoside phosphate binding (GO:1901265)  small molecule binding (GO:0036094)  nucleoside-triphosphatase activity (GO:0017111)  purine ribonucleoside triphosphate binding (GO:0035639)  purine ribonucleoside binding (GO:0032550)  purine nucleoside binding (GO:0001883)  ribonucleoside binding (GO:0032549)  nucleoside binding (GO:0001882)  purine ribonucleotide binding (GO:0032555)  ribonucleotide binding (GO:0032553)  purine nucleotide binding (GO:0017076)  double-stranded RNA binding (GO:0003725)  nucleic acid binding (GO:0003676)  structural molecule activity (GO:0005198)  molecular_function (GO:0003674)  carbohydrate derivative binding (GO:0097367)  structural constituent of cytoskeleton (GO:0005200)  unfolded protein binding (GO:0051082) | extracellular organelle (GO:0043230)  extracellular vesicular exosome (GO:0070062)  extracellular membrane-bounded organelle (GO:0065010)  extracellular region part (GO:0044421)  membrane-bounded vesicle (GO:0031988)  vesicle (GO:0031982)  extracellular region (GO:0005576)  macromolecular complex (GO:0032991)  cytoplasmic part (GO:0044444)  organelle part (GO:0044422)  cytosol (GO:0005829)  intracellular organelle part (GO:0044446)  nucleus (GO:0005634)  intracellular organelle (GO:0043229)  intracellular part (GO:0044424)  intracellular (GO:0005622)  cytoplasm (GO:0005737)  organelle (GO:0043226)  membrane-bounded organelle (GO:0043227)  intracellular non-membrane-bounded organelle (GO:0043232)  non-membrane-bounded organelle (GO:0043228)  protein complex (GO:0043234)  mitochondrion (GO:0005739)  COP9 signalosome (GO:0008180)  organelle lumen (GO:0043233)  membrane-enclosed lumen (GO:0031974)  intracellular membrane-bounded organelle (GO:0043231)  nuclear part (GO:0044428)  cell part (GO:0044464)  cell (GO:0005623)  intracellular organelle lumen (GO:0070013)  histone acetyltransferase complex (GO:0000123)  protein acetyltransferase complex (GO:0031248)  acetyltransferase complex (GO:1902493)  NuA4 histone acetyltransferase complex (GO:0035267)  H4 histone acetyltransferase complex (GO:1902562)  H4/H2A histone acetyltransferase complex (GO:0043189) |
| **AARS2, AP2B1, AP2M1, ASAP2, CAPZB, CASK, CHM, COPS2, EEF1A1, GDA, ITSN1, MEGF10, MLH1, MPST, MRPL20, SF3A3, SIRPA, SMNDC1, TMSB4X, TRIM2, UBE2E1, UIMC1** | cellular process (GO:0009987) | protein binding (GO:0005515)  binding (GO:0005488) | coated pit (GO:0005905)  cell part (GO:0044464)  cell (GO:0005623) |
| **BTF3, CCND1, CDC34, CDK6, CTNNBIP1, ESR1, PPP1CB, PPP1CC, RPA3, SH2D4A, SH3GL1, SH3GL2, TCEA2, TIMM13** | negative regulation of response to stimulus (GO:0048585)  negative regulation of signal transduction (GO:0009968)  negative regulation of signaling (GO:0023057)  negative regulation of cell communication (GO:0010648)  mitotic cell cycle phase transition (GO:0044772)  cell cycle phase transition (GO:0044770)  mitotic cell cycle (GO:0000278)  cell cycle G1/S phase transition (GO:0044843)  G1/S transition of mitotic cell cycle (GO:0000082)  mammary gland epithelial cell proliferation (GO:0033598)  entrainment of circadian clock by photoperiod (GO:0043153)  cell cycle phase (GO:0022403)  mitotic cell cycle process (GO:1903047)  biological phase (GO:0044848)  entrainment of circadian clock (GO:0009649)  mammary gland lobule development (GO:0061377)  mammary gland alveolus development (GO:0060749)  photoperiodism (GO:0009648)  cell cycle (GO:0007049)  triglyceride catabolic process (GO:0019433)  acylglycerol catabolic process (GO:0046464)  neutral lipid catabolic process (GO:0046461)  mitotic interphase (GO:0051329)  interphase (GO:0051325)  glycerolipid catabolic process (GO:0046503)  regulation of response to stimulus (GO:0048583)  G1 phase (GO:0051318)  mitotic G1 phase (GO:0000080)  cell cycle process (GO:0022402)  regulation of signal transduction (GO:0009966) | protein binding (GO:0005515)  enzyme binding (GO:0019899)  binding (GO:0005488) | nuclear lumen (GO:0031981)  PTW/PP1 phosphatase complex (GO:0072357)  MLL5-L complex (GO:0070688)  nuclear part (GO:0044428)  cyclin-dependent protein kinase holoenzyme complex (GO:0000307)  nucleolus (GO:0005730)  intracellular organelle lumen (GO:0070013)  organelle lumen (GO:0043233)  membrane-enclosed lumen (GO:0031974)  protein complex (GO:0043234)  nucleus (GO:0005634)  nucleoplasm (GO:0005654)  intracellular non-membrane-bounded organelle (GO:0043232)  non-membrane-bounded organelle (GO:0043228)  intracellular membrane-bounded organelle (GO:0043231)  protein serine/threonine phosphatase complex (GO:0008287)  phosphatase complex (GO:1903293)  transferase complex (GO:1990234)  macromolecular complex (GO:0032991)  intracellular part (GO:0044424)  intracellular organelle part (GO:0044446)  intracellular (GO:0005622)  organelle part (GO:0044422) |
| **APP, CAT, CBFB, DBN1, DLGAP5, HSD17B10, HSPA4, HSPA6, IKBKG, MAP2, P4HB, PDIA3, PPIA, PRSS23, PTPRN, PTPRS, RG9MTD1, SCHIP1, SFXN1, SLC8A1, STXBP1, SUPT5H, TAB1, TAP1, TRAF2, TUBB2B, TUFM** | response to stress (GO:0006950)  single-organism cellular process (GO:0044763)  phosphate-containing compound metabolic process (GO:0006796)  phosphorus metabolic process (GO:0006793)  protein folding (GO:0006457)  positive regulation of NF-kappaB transcription factor activity (GO:0051092)  nucleotide-binding domain, leucine rich repeat containing receptor signaling pathway (GO:0035872)  single-organism process (GO:0044699)  cell communication by electrical coupling (GO:0010644)  cellular metabolic process (GO:0044237)  protein metabolic process (GO:0019538)  positive regulation of sequence-specific DNA binding transcription factor activity (GO:0051091)  platelet degranulation (GO:0002576)  metabolic process (GO:0008152) | protein binding (GO:0005515)  binding (GO:0005488)  enzyme binding (GO:0019899)  molecular_function (GO:0003674)  identical protein binding (GO:0042802)  transmembrane receptor protein tyrosine phosphatase activity (GO:0005001)  transmembrane receptor protein phosphatase activity (GO:0019198)  hydrolase activity (GO:0016787)  poly(A) RNA binding (GO:0044822)  intramolecular oxidoreductase activity, transposing S-S bonds (GO:0016864)  protein disulfide isomerase activity (GO:0003756)  catalytic activity (GO:0003824) | cell part (GO:0044464)  cell (GO:0005623)  extracellular organelle (GO:0043230)  extracellular vesicular exosome (GO:0070062)  extracellular membrane-bounded organelle (GO:0065010)  intracellular part (GO:0044424)  cytoplasm (GO:0005737)  intracellular (GO:0005622)  organelle (GO:0043226)  intracellular organelle (GO:0043229) |
| **ACTG1, ADAM22, CFL1, EPB41L2, KIF5C, KLC3, NCKAP1, OSBPL3, RAB5A, RABEP1, YWHAB, YWHAE, YWHAH, YWHAQ, YWHAZ** | positive regulation of protein insertion into mitochondrial membrane involved in apoptotic signaling pathway (GO:1900740)  regulation of protein insertion into mitochondrial membrane involved in apoptotic signaling pathway (GO:1900739)  regulation of mitochondrial outer membrane permeabilization involved in apoptotic signaling pathway (GO:1901028)  positive regulation of mitochondrion organization (GO:0010822)  positive regulation of organelle organization (GO:0010638)  macromolecule localization (GO:0033036)  transport (GO:0006810)  regulation of mitochondrion organization (GO:0010821)  establishment of localization (GO:0051234)  organic substance transport (GO:0071702)  localization (GO:0051179)  intrinsic apoptotic signaling pathway (GO:0097193)  protein localization (GO:0008104)  positive regulation of cellular component organization (GO:0051130)  membrane organization (GO:0061024)  regulation of organelle organization (GO:0033043)  protein transport (GO:0015031)  establishment of protein localization (GO:0045184)  regulation of protein localization (GO:0032880)  nervous system development (GO:0007399)  single-organism transport (GO:0044765)  intracellular protein transport (GO:0006886)  intracellular signal transduction (GO:0035556)  apoptotic signaling pathway (GO:0097190)  apoptotic process (GO:0006915)  regulation of apoptotic signaling pathway (GO:2001233)  programmed cell death (GO:0012501)  single-organism localization (GO:1902578)  regulation of cellular component organization (GO:0051128)  substantia nigra development (GO:0021762)  protein targeting (GO:0006605)  negative regulation of protein dephosphorylation (GO:0035308)  regulation of intracellular transport (GO:0032386)  subthalamus development (GO:0021539)  intracellular transport (GO:0046907)  endocytosis (GO:0006897)  regulation of establishment of protein localization (GO:0070201)  small GTPase mediated signal transduction (GO:0007264)  cellular component movement (GO:0006928)  negative regulation of dephosphorylation (GO:0035305)  cell death (GO:0008219)  death (GO:0016265)  cellular component organization (GO:0016043)  neural nucleus development (GO:0048857)  cellular component organization or biogenesis (GO:0071840)  regulation of cellular localization (GO:0060341)  Fc receptor signaling pathway (GO:0038093)  immune response-regulating cell surface receptor signaling pathway involved in phagocytosis (GO:0002433)  Fc-gamma receptor signaling pathway involved in phagocytosis (GO:0038096)  Fc receptor mediated stimulatory signaling pathway (GO:0002431)  Fc-gamma receptor signaling pathway (GO:0038094)  single-organism intracellular transport (GO:1902582)  cellular protein localization (GO:0034613)  cellular macromolecule localization (GO:0070727)  vesicle-mediated transport (GO:0016192)  establishment of localization in cell (GO:0051649)  immune response-regulating cell surface receptor signaling pathway (GO:0002768)  diencephalon development (GO:0021536)  actin cytoskeleton organization (GO:0030036)  regulation of protein dephosphorylation (GO:0035304)  regulation of localization (GO:0032879)  cellular localization (GO:0051641)  regulation of apoptotic process (GO:0042981)  actin filament-based process (GO:0030029)  regulation of programmed cell death (GO:0043067)  phagocytosis (GO:0006909)  neurogenesis (GO:0022008)  single-organism process (GO:0044699)  immune effector process (GO:0002252)  Ras protein signal transduction (GO:0007265)  central nervous system development (GO:0007417)  regulation of biological quality (GO:0065008)  immune response-regulating signaling pathway (GO:0002764)  system development (GO:0048731)  hippo signaling (GO:0035329)  regulation of cell death (GO:0010941)  regulation of transport (GO:0051049)  immune response-activating cell surface receptor signaling pathway (GO:0002429)  regulation of response to stimulus (GO:0048583)  single-organism cellular process (GO:0044763) | protein complex binding (GO:0032403)  protein binding (GO:0005515)  protein domain specific binding (GO:0019904)  phosphoserine binding (GO:0050815)  protein phosphorylated amino acid binding (GO:0045309)  cytoskeletal protein binding (GO:0008092)  binding (GO:0005488)  phosphoprotein binding (GO:0051219) | membrane-bounded vesicle (GO:0031988)  vesicle (GO:0031982)  extracellular organelle (GO:0043230)  extracellular vesicular exosome (GO:0070062)  extracellular membrane-bounded organelle (GO:0065010)  melanosome (GO:0042470)  pigment granule (GO:0048770)  cytoplasmic membrane-bounded vesicle (GO:0016023)  cytoplasmic vesicle (GO:0031410)  cytoplasmic vesicle membrane (GO:0030659)  vesicle membrane (GO:0012506)  kinesin complex (GO:0005871)  extracellular region part (GO:0044421)  cytoskeleton (GO:0005856)  cytosol (GO:0005829)  cytoskeletal part (GO:0044430)  cytoplasmic vesicle part (GO:0044433)  cell leading edge (GO:0031252)  lamellipodium membrane (GO:0031258)  extracellular region (GO:0005576)  cytoplasm (GO:0005737)  microtubule associated complex (GO:0005875)  actin cytoskeleton (GO:0015629)  intracellular organelle part (GO:0044446)  cytoplasmic part (GO:0044444)  organelle part (GO:0044422)  cortical actin cytoskeleton (GO:0030864) |
| **ANK2, CDK5, CDKN1B, CLU, PPARG, PSMC5, PSMD1, STMN1, TAF9, UBA1** | negative regulation of protein metabolic process (GO:0051248)  regulation of cell cycle arrest (GO:0071156)  regulation of apoptotic process (GO:0042981)  cell death (GO:0008219)  death (GO:0016265)  regulation of programmed cell death (GO:0043067)  regulation of cell death (GO:0010941)  positive regulation of macromolecule metabolic process (GO:0010604)  regulation of transferase activity (GO:0051338)  positive regulation of metabolic process (GO:0009893)  positive regulation of molecular function (GO:0044093)  regulation of cellular component organization (GO:0051128)  negative regulation of macromolecule metabolic process (GO:0010605)  negative regulation of cellular metabolic process (GO:0031324)  regulation of proteolysis (GO:0030162)  regulation of protein ubiquitination (GO:0031396)  regulation of protein catabolic process (GO:0042176)  positive regulation of inclusion body assembly (GO:0090261)  negative regulation of cellular protein metabolic process (GO:0032269)  negative regulation of metabolic process (GO:0009892)  regulation of protein modification by small protein conjugation or removal (GO:1903320)  apoptotic process (GO:0006915)  programmed cell death (GO:0012501)  positive regulation of biological process (GO:0048518)  DNA damage response, signal transduction by p53 class mediator resulting in cell cycle arrest (GO:0006977)  signal transduction involved in mitotic G1 DNA damage checkpoint (GO:0072431)  intracellular signal transduction involved in G1 DNA damage checkpoint (GO:1902400)  signal transduction involved in DNA damage checkpoint (GO:0072422)  signal transduction involved in mitotic cell cycle checkpoint (GO:0072413)  signal transduction involved in DNA integrity checkpoint (GO:0072401)  signal transduction involved in mitotic DNA integrity checkpoint (GO:1902403)  signal transduction involved in mitotic DNA damage checkpoint (GO:1902402)  signal transduction involved in cell cycle checkpoint (GO:0072395)  regulation of protein metabolic process (GO:0051246)  cellular response to DNA damage stimulus (GO:0006974)  mitotic G1/S transition checkpoint (GO:0044819)  mitotic G1 DNA damage checkpoint (GO:0031571)  response to stress (GO:0006950)  G1 DNA damage checkpoint (GO:0044783)  negative regulation of transferase activity (GO:0051348)  positive regulation of cell cycle arrest (GO:0071158)  protein stabilization (GO:0050821)  positive regulation of catalytic activity (GO:0043085)  negative regulation of cellular process (GO:0048523)  negative regulation of cell death (GO:0060548)  DNA damage response, signal transduction by p53 class mediator (GO:0030330)  regulation of cell growth (GO:0001558)  mitotic DNA damage checkpoint (GO:0044773)  negative regulation of G1/S transition of mitotic cell cycle (GO:2000134)  negative regulation of cell cycle G1/S phase transition (GO:1902807)  mitotic DNA integrity checkpoint (GO:0044774)  response to stimulus (GO:0050896)  signal transduction in response to DNA damage (GO:0042770)  negative regulation of protein ubiquitination (GO:0031397)  negative regulation of biological process (GO:0048519)  negative regulation of protein modification by small protein conjugation or removal (GO:1903321)  intracellular signal transduction (GO:0035556)  regulation of molecular function (GO:0065009)  regulation of cellular protein metabolic process (GO:0032268)  protein metabolic process (GO:0019538)  positive regulation of cellular process (GO:0048522)  S phase (GO:0051320)  mitotic S phase (GO:0000084)  regulation of G1/S transition of mitotic cell cycle (GO:2000045)  negative regulation of protein modification process (GO:0031400)  regulation of cell cycle G1/S phase transition (GO:1902806)  cellular response to lithium ion (GO:0071285)  regulation of inclusion body assembly (GO:0090083)  signal transduction by p53 class mediator (GO:0072331)  regulation of protein stability (GO:0031647)  DNA damage checkpoint (GO:0000077)  positive regulation of protein ubiquitination (GO:0031398)  cellular response to stimulus (GO:0051716)  regulation of cellular component biogenesis (GO:0044087)  mitotic interphase (GO:0051329)  interphase (GO:0051325)  DNA integrity checkpoint (GO:0031570)  regulation of cell cycle process (GO:0010564)  negative regulation of cell growth (GO:0030308)  positive regulation of protein modification by small protein conjugation or removal (GO:1903322)  regulation of microtubule polymerization (GO:0031113)  positive regulation of protein metabolic process (GO:0051247)  mitotic cell cycle checkpoint (GO:0007093)  regulation of cellular ketone metabolic process (GO:0010565)  cell cycle G1/S phase transition (GO:0044843)  G1/S transition of mitotic cell cycle (GO:0000082)  negative regulation of nitrogen compound metabolic process (GO:0051172)  response to lithium ion (GO:0010226)  regulation of protein processing (GO:0070613)  regulation of protein maturation (GO:1903317)  negative regulation of intrinsic apoptotic signaling pathway in response to DNA damage (GO:1902230)  negative regulation of mitotic cell cycle phase transition (GO:1901991)  negative regulation of cell cycle phase transition (GO:1901988)  positive regulation of transferase activity (GO:0051347)  regulation of growth (GO:0040008)  regulation of cellular component movement (GO:0051270)  regulation of catalytic activity (GO:0050790)  regulation of intrinsic apoptotic signaling pathway in response to DNA damage (GO:1902229)  negative regulation of growth (GO:0045926)  regulation of protein modification process (GO:0031399)  positive regulation of cell cycle process (GO:0090068)  cell cycle (GO:0007049)  regulation of protein complex assembly (GO:0043254)  neurogenesis (GO:0022008)  cellular response to stress (GO:0033554)  regulation of mitotic cell cycle phase transition (GO:1901990)  response to organic substance (GO:0010033)  mitotic cell cycle process (GO:1903047)  negative regulation of apoptotic process (GO:0043066)  negative regulation of cell cycle process (GO:0010948)  positive regulation of cellular component organization (GO:0051130)  single organism signaling (GO:0044700)  signaling (GO:0023052)  negative regulation of response to DNA damage stimulus (GO:2001021)  positive regulation of cellular metabolic process (GO:0031325)  regulation of cell cycle phase transition (GO:1901987)  cell cycle checkpoint (GO:0000075)  negative regulation of programmed cell death (GO:0043069)  cell communication (GO:0007154) | p53 binding (GO:0002039)  activating transcription factor binding (GO:0033613)  acetylcholine receptor activator activity (GO:0030549)  thyrotropin-releasing hormone receptor binding (GO:0031531)  transcription factor binding (GO:0008134)  acetylcholine receptor regulator activity (GO:0030548) | cytosol (GO:0005829)  organelle part (GO:0044422)  proteasome accessory complex (GO:0022624)  macromolecular complex (GO:0032991)  inclusion body (GO:0016234)  nucleoplasm (GO:0005654)  intracellular organelle part (GO:0044446)  cytoplasmic part (GO:0044444)  proteasome complex (GO:0000502) |
| **ANXA2, BAG6, C1QBP, CDH1, CTSB, DCXR, DMWD, ECHS1, GABARAPL2, GLO1, GSPT1, GSTK1, IKBKE, LMNA, NCDN, NCKIPSD, NIPSNAP1, PDXK, PGRMC2, RGS2, SRM, STAC** | single-organism process (GO:0044699)  endomembrane system organization (GO:0010256)  single-organism cellular localization (GO:1902580)  negative regulation of proteolysis involved in cellular protein catabolic process (GO:1903051)  negative regulation of proteasomal protein catabolic process (GO:1901799)  activation of immune response (GO:0002253)  osteoclast differentiation (GO:0030316)  cellular component disassembly (GO:0022411)  negative regulation of multicellular organismal process (GO:0051241) | beta-tubulin binding (GO:0048487)  ribosome binding (GO:0043022) | cytoplasmic part (GO:0044444)  cytosol (GO:0005829)  cytoplasm (GO:0005737)  Schmidt-Lanterman incisure (GO:0043220)  compact myelin (GO:0043218) |
| **AES, ATPIF1, CBL, CEP76, DGKE, FABP3, GAB1, GABARAP, GFAP, HIST4H4, INPPL1, MAP1LC3C, NBR1, NEDD4, PAICS, PDHA1, PDHB, PIK3CA, PTPRO, RIBC2, RPL18A, SORBS1, SQSTM1, VPS28** | response to insulin (GO:0032868)  response to peptide hormone (GO:0043434)  acetyl-CoA biosynthetic process from pyruvate (GO:0006086)  response to peptide (GO:1901652)  autophagy (GO:0006914)  cellular metabolic process (GO:0044237)  negative regulation of cellular process (GO:0048523)  acetyl-CoA biosynthetic process (GO:0006085)  glucose metabolic process (GO:0006006)  negative regulation of biological process (GO:0048519)  regulation of cellular process (GO:0050794)  hexose metabolic process (GO:0019318)  response to organic substance (GO:0010033)  cellular response to insulin stimulus (GO:0032869)  regulation of fatty acid metabolic process (GO:0019217)  response to organonitrogen compound (GO:0010243)  single-organism cellular process (GO:0044763)  ubiquitin-dependent protein catabolic process via the multivesicular body sorting pathway (GO:0043162)  regulation of acetyl-CoA biosynthetic process from pyruvate (GO:0010510)  metabolic process (GO:0008152)  monosaccharide metabolic process (GO:0005996)  regulation of biological process (GO:0050789)  regulation of acyl-CoA biosynthetic process (GO:0050812)  cellular process (GO:0009987)  regulation of coenzyme metabolic process (GO:0051196)  regulation of cofactor metabolic process (GO:0051193)  response to nitrogen compound (GO:1901698)  single-organism process (GO:0044699)  regulation of lipid metabolic process (GO:0019216)  response to hormone (GO:0009725)  single-organism biosynthetic process (GO:0044711) | pyruvate dehydrogenase (acetyl-transferring) activity (GO:0004739)  pyruvate dehydrogenase activity (GO:0004738)  protein binding (GO:0005515)  oxidoreductase activity, acting on the aldehyde or oxo group of donors, disulfide as acceptor (GO:0016624)  ubiquitin binding (GO:0043130)  small conjugating protein binding (GO:0032182)  kinase binding (GO:0019900)  binding (GO:0005488)  protein phosphorylated amino acid binding (GO:0045309)  enzyme binding (GO:0019899) | cytosol (GO:0005829)  autophagic vacuole (GO:0005776)  cytoplasmic part (GO:0044444)  macromolecular complex (GO:0032991)  cytoplasm (GO:0005737)  pyruvate dehydrogenase complex (GO:0045254)  intracellular part (GO:0044424)  intracellular (GO:0005622)  protein complex (GO:0043234)  cell part (GO:0044464)  cell (GO:0005623)  autophagic vacuole membrane (GO:0000421)  catalytic complex (GO:1902494)  pre-autophagosomal structure (GO:0000407) |
| **ACTR1A, AIFM1, CTSD, CYB5B, DCC, EIF4A2, ERP29, ESD, GNG12, GOT1, H2AFX, HADHA, HLA-B, LMNB1, MAPK1, MCC, PACSIN3, PEBP1, PRDX3, PREP, RPL10A, SPG20, STC2** | negative regulation of collateral sprouting (GO:0048671)  response to peptide hormone (GO:0043434)  response to peptide (GO:1901652)  response to organonitrogen compound (GO:0010243)  response to nitrogen compound (GO:1901698)  regulation of collateral sprouting (GO:0048670)  single-organism process (GO:0044699)  response to organic substance (GO:0010033)  cell death (GO:0008219)  death (GO:0016265)  cellular catabolic process (GO:0044248)  polyamine metabolic process (GO:0006595)  maternal placenta development (GO:0001893)  regulation of mitochondrial membrane potential (GO:0051881) | binding (GO:0005488)  molecular_function (GO:0003674)  protein binding (GO:0005515) | intracellular part (GO:0044424)  intracellular (GO:0005622)  extracellular organelle (GO:0043230)  extracellular vesicular exosome (GO:0070062)  extracellular membrane-bounded organelle (GO:0065010)  membrane-bounded vesicle (GO:0031988)  vesicle (GO:0031982)  cytoplasm (GO:0005737)  membrane-bounded organelle (GO:0043227)  extracellular region part (GO:0044421)  cytoplasmic part (GO:0044444)  membrane-enclosed lumen (GO:0031974)  mitochondrion (GO:0005739)  cell part (GO:0044464)  cell (GO:0005623)  mitochondrial part (GO:0044429)  organelle (GO:0043226)  intracellular organelle part (GO:0044446)  intracellular organelle lumen (GO:0070013)  organelle lumen (GO:0043233)  extracellular region (GO:0005576)  organelle part (GO:0044422) |
| **BRE, CAPN2, CAPNS1, CNP, GNAI1, GNB1, GNB2, GNB4, LRPPRC, MTNR1A, MTNR1B, RAB1A, SLC25A13, STAU1, SYT5, TERF1, TUBA1A, TUBB3, USP32, USP50, VPS35** | GTP catabolic process (GO:0006184)  guanosine-containing compound catabolic process (GO:1901069)  GTP metabolic process (GO:0046039)  guanosine-containing compound metabolic process (GO:1901068)  nucleotide catabolic process (GO:0009166)  nucleoside phosphate catabolic process (GO:1901292)  organophosphate catabolic process (GO:0046434)  nucleotide metabolic process (GO:0009117)  nucleoside phosphate metabolic process (GO:0006753)  purine ribonucleoside triphosphate metabolic process (GO:0009205)  ribonucleoside triphosphate metabolic process (GO:0009199)  purine nucleoside triphosphate metabolic process (GO:0009144)  nucleoside triphosphate metabolic process (GO:0009141)  nucleobase-containing small molecule metabolic process (GO:0055086)  purine ribonucleoside metabolic process (GO:0046128)  purine nucleoside metabolic process (GO:0042278)  ribonucleoside metabolic process (GO:0009119)  purine ribonucleotide metabolic process (GO:0009150)  nucleoside metabolic process (GO:0009116)  ribonucleotide metabolic process (GO:0009259)  ribose phosphate metabolic process (GO:0019693)  purine nucleotide metabolic process (GO:0006163)  glycosyl compound metabolic process (GO:1901657)  cellular response to glucagon stimulus (GO:0071377)  purine-containing compound metabolic process (GO:0072521)  purine ribonucleoside triphosphate catabolic process (GO:0009207)  ribonucleoside triphosphate catabolic process (GO:0009203)  purine nucleoside triphosphate catabolic process (GO:0009146)  nucleoside triphosphate catabolic process (GO:0009143)  purine ribonucleoside catabolic process (GO:0046130)  purine nucleoside catabolic process (GO:0006152)  ribonucleoside catabolic process (GO:0042454)  purine ribonucleotide catabolic process (GO:0009154)  ribonucleotide catabolic process (GO:0009261)  nucleoside catabolic process (GO:0009164)  purine nucleotide catabolic process (GO:0006195)  glycosyl compound catabolic process (GO:1901658)  energy derivation by oxidation of organic compounds (GO:0015980)  purine-containing compound catabolic process (GO:0072523)  organophosphate metabolic process (GO:0019637)  nucleobase-containing compound catabolic process (GO:0034655)  energy reserve metabolic process (GO:0006112)  response to glucagon (GO:0033762)  G-protein coupled receptor signaling pathway, coupled to cyclic nucleotide second messenger (GO:0007187)  synaptic transmission (GO:0007268)  cell-cell signaling (GO:0007267)  cellular nitrogen compound catabolic process (GO:0044270)  aromatic compound catabolic process (GO:0019439)  heterocycle catabolic process (GO:0046700)  cellular catabolic process (GO:0044248)  organic cyclic compound catabolic process (GO:1901361)  generation of precursor metabolites and energy (GO:0006091)  organic substance catabolic process (GO:1901575)  carbohydrate derivative catabolic process (GO:1901136)  microtubule-based process (GO:0007017)  small molecule metabolic process (GO:0044281)  catabolic process (GO:0009056)  cytoskeleton-dependent intracellular transport (GO:0030705)  organonitrogen compound catabolic process (GO:1901565)  single-organism catabolic process (GO:0044712)  single-organism intracellular transport (GO:1902582)  cellular localization (GO:0051641)  single-organism metabolic process (GO:0044710)  carbohydrate derivative metabolic process (GO:1901135)  response to peptide hormone (GO:0043434)  response to organonitrogen compound (GO:0010243) | GTPase activity (GO:0003924)  melatonin receptor activity (GO:0008502)  hydrolase activity (GO:0016787)  nucleoside-triphosphatase activity (GO:0017111)  protein complex binding (GO:0032403)  protein binding (GO:0005515)  pyrophosphatase activity (GO:0016462)  hydrolase activity, acting on acid anhydrides, in phosphorus-containing anhydrides (GO:0016818)  hydrolase activity, acting on acid anhydrides (GO:0016817)  binding (GO:0005488)  calcium-dependent cysteine-type endopeptidase activity (GO:0004198)  GTP binding (GO:0005525) | lysosomal membrane (GO:0005765)  vacuolar membrane (GO:0005774)  vacuolar part (GO:0044437)  microtubule cytoskeleton (GO:0015630)  cytoskeletal part (GO:0044430)  extracellular organelle (GO:0043230)  extracellular vesicular exosome (GO:0070062)  extracellular membrane-bounded organelle (GO:0065010)  macromolecular complex (GO:0032991)  intracellular organelle part (GO:0044446)  membrane-bounded vesicle (GO:0031988)  vesicle (GO:0031982)  organelle part (GO:0044422)  bounding membrane of organelle (GO:0098588)  lytic vacuole (GO:0000323)  lysosome (GO:0005764)  organelle membrane (GO:0031090)  pseudopodium (GO:0031143)  cytoplasm (GO:0005737)  vacuole (GO:0005773)  perinuclear region of cytoplasm (GO:0048471)  cytoskeleton (GO:0005856)  protein complex (GO:0043234)  cytoplasmic part (GO:0044444) |
| **C19ORF62, COX2, COX5A, COX6B1, COX6C, CSTB, DAZAP2, EIF4H, GZMA, HSPA1A, LASP1, LIN54, MDM2, NDUFA12, NDUFA2, NDUFA4, NDUFA5, NDUFA8, NDUFA9, NDUFB5, NDUFB7, NDUFS1, NDUFS3, PHB, PRDX5, PSMF1, PTPRD, RHOXF2, RNH1, TNIP3, TOLLIP, USP9X** | respiratory electron transport chain (GO:0022904)  electron transport chain (GO:0022900)  cellular respiration (GO:0045333)  mitochondrial ATP synthesis coupled electron transport (GO:0042775)  ATP synthesis coupled electron transport (GO:0042773)  energy derivation by oxidation of organic compounds (GO:0015980)  oxidative phosphorylation (GO:0006119)  mitochondrial electron transport, NADH to ubiquinone (GO:0006120)  generation of precursor metabolites and energy (GO:0006091)  oxidation-reduction process (GO:0055114)  ATP metabolic process (GO:0046034)  purine ribonucleoside monophosphate metabolic process (GO:0009167)  purine nucleoside monophosphate metabolic process (GO:0009126)  ribonucleoside monophosphate metabolic process (GO:0009161)  nucleoside monophosphate metabolic process (GO:0009123)  purine ribonucleoside triphosphate metabolic process (GO:0009205)  ribonucleoside triphosphate metabolic process (GO:0009199)  purine nucleoside triphosphate metabolic process (GO:0009144)  nucleoside triphosphate metabolic process (GO:0009141)  nucleotide metabolic process (GO:0009117)  purine ribonucleoside metabolic process (GO:0046128)  purine nucleoside metabolic process (GO:0042278)  nucleoside phosphate metabolic process (GO:0006753)  ribonucleoside metabolic process (GO:0009119)  purine ribonucleotide metabolic process (GO:0009150)  nucleoside metabolic process (GO:0009116)  ribonucleotide metabolic process (GO:0009259)  nucleobase-containing small molecule metabolic process (GO:0055086)  ribose phosphate metabolic process (GO:0019693)  purine nucleotide metabolic process (GO:0006163)  glycosyl compound metabolic process (GO:1901657)  purine-containing compound metabolic process (GO:0072521)  small molecule metabolic process (GO:0044281)  organophosphate metabolic process (GO:0019637)  phosphorylation (GO:0016310)  cellular metabolic process (GO:0044237)  hydrogen ion transmembrane transport (GO:1902600)  phosphate-containing compound metabolic process (GO:0006796)  phosphorus metabolic process (GO:0006793)  proton transport (GO:0015992)  hydrogen transport (GO:0006818)  carbohydrate derivative metabolic process (GO:1901135)  organonitrogen compound metabolic process (GO:1901564)  single-organism metabolic process (GO:0044710)  metabolic process (GO:0008152)  monovalent inorganic cation transport (GO:0015672)  single-organism process (GO:0044699)  nucleobase-containing compound metabolic process (GO:0006139)  single-organism cellular process (GO:0044763)  heterocycle metabolic process (GO:0046483)  cellular aromatic compound metabolic process (GO:0006725)  cellular nitrogen compound metabolic process (GO:0034641)  organic cyclic compound metabolic process (GO:1901360)  negative regulation of hydrolase activity (GO:0051346)  negative regulation of protein processing (GO:0010955)  negative regulation of protein maturation (GO:1903318)  reactive oxygen species metabolic process (GO:0072593)  negative regulation of endopeptidase activity (GO:0010951) | NADH dehydrogenase (quinone) activity (GO:0050136)  NADH dehydrogenase activity (GO:0003954)  NADH dehydrogenase (ubiquinone) activity (GO:0008137)  oxidoreductase activity, acting on NAD(P)H, quinone or similar compound as acceptor (GO:0016655)  oxidoreductase activity, acting on NAD(P)H (GO:0016651)  oxidoreductase activity (GO:0016491)  electron carrier activity (GO:0009055)  heme-copper terminal oxidase activity (GO:0015002)  cytochrome-c oxidase activity (GO:0004129)  oxidoreductase activity, acting on a heme group of donors, oxygen as acceptor (GO:0016676)  oxidoreductase activity, acting on a heme group of donors (GO:0016675)  hydrogen ion transmembrane transporter activity (GO:0015078)  catalytic activity (GO:0003824)  molecular_function (GO:0003674)  monovalent inorganic cation transmembrane transporter activity (GO:0015077)  protein binding (GO:0005515) | respiratory chain complex I (GO:0045271)  NADH dehydrogenase complex (GO:0030964)  mitochondrial respiratory chain complex I (GO:0005747)  respiratory chain (GO:0070469)  mitochondrial inner membrane (GO:0005743)  organelle inner membrane (GO:0019866)  mitochondrial respiratory chain (GO:0005746)  oxidoreductase complex (GO:1990204)  mitochondrial membrane (GO:0031966)  mitochondrial envelope (GO:0005740)  mitochondrial part (GO:0044429)  mitochondrial membrane part (GO:0044455)  mitochondrion (GO:0005739)  organelle envelope (GO:0031967)  envelope (GO:0031975)  organelle membrane (GO:0031090)  catalytic complex (GO:1902494)  cytoplasmic part (GO:0044444)  intracellular organelle part (GO:0044446)  protein complex (GO:0043234)  organelle part (GO:0044422)  mitochondrial intermembrane space (GO:0005758)  organelle envelope lumen (GO:0031970)  cytoplasm (GO:0005737)  intracellular part (GO:0044424)  intracellular membrane-bounded organelle (GO:0043231)  intracellular (GO:0005622)  membrane-bounded organelle (GO:0043227)  intracellular organelle (GO:0043229)  macromolecular complex (GO:0032991)  respiratory chain complex IV (GO:0045277)  cell part (GO:0044464)  cell (GO:0005623)  organelle (GO:0043226)  membrane-enclosed lumen (GO:0031974)  membrane (GO:0016020)  cytochrome complex (GO:0070069) |
| **ANK3, ANXA3, ARL15, ARL3, ATP6V1D, BCR, CHMP7, GAPDH, GRIPAP1, IGSF21, LAMA4, LRRK1, MYO5A, NUDT21, PAFAH1B3, RIMS1, RPLP1, SMAD2, TUBB2A, UBR1** | cellular component organization (GO:0016043)  cellular localization (GO:0051641)  cellular component organization or biogenesis (GO:0071840)  protein transport (GO:0015031)  establishment of localization in cell (GO:0051649)  establishment of protein localization (GO:0045184)  organic substance transport (GO:0071702)  protein localization (GO:0008104)  signal release (GO:0023061)  membrane organization (GO:0061024)  single-organism transport (GO:0044765)  insulin secretion (GO:0030073) | GTP binding (GO:0005525)  guanyl ribonucleotide binding (GO:0032561)  guanyl nucleotide binding (GO:0019001) | extracellular organelle (GO:0043230)  extracellular vesicular exosome (GO:0070062)  extracellular membrane-bounded organelle (GO:0065010)  membrane-bounded vesicle (GO:0031988)  vesicle (GO:0031982)  extracellular region part (GO:0044421)  extracellular region (GO:0005576)  macromolecular complex (GO:0032991)  microtubule cytoskeleton (GO:0015630)  protein complex (GO:0043234) |
| **ABI2, ATL1, CBR1, CCDC53, CDKN2A, CKB, CPLX2, EIF2C3, EXOC7, GOT2, HGS, HIST1H2BF, IFT20, KIF5B, NAPA, NME2, OXCT1, PCBD1, RTN4, SNAP23, SNAP25, SSBP1, STIP1, SYT1, TFCP2, TUBA1C, TUBB2C, UBQLN1, USPL1, VDAC1, ZDHHC17** | establishment of vesicle localization (GO:0051650)  organelle localization (GO:0051640)  vesicle localization (GO:0051648)  establishment of organelle localization (GO:0051656)  synaptic vesicle exocytosis (GO:0016079)  vesicle docking (GO:0048278)  exocytosis (GO:0006887)  membrane docking (GO:0022406)  cellular ketone metabolic process (GO:0042180)  cellular localization (GO:0051641)  biological_process (GO:0008150)  cellular process (GO:0009987)  single-organism transport (GO:0044765)  synaptic vesicle transport (GO:0048489)  establishment of synaptic vesicle localization (GO:0097480)  localization (GO:0051179)  cellular component organization (GO:0016043)  synaptic vesicle localization (GO:0097479)  establishment of localization in cell (GO:0051649)  neurotransmitter secretion (GO:0007269)  cellular component organization or biogenesis (GO:0071840)  single-organism cellular process (GO:0044763)  single-organism localization (GO:1902578)  secretion by cell (GO:0032940)  glutamate secretion (GO:0014047) | protein binding (GO:0005515)  SNARE binding (GO:0000149)  binding (GO:0005488)  syntaxin binding (GO:0019905)  molecular_function (GO:0003674)  phosphotransferase activity, nitrogenous group as acceptor (GO:0016775)  syntaxin-1 binding (GO:0017075) | cytoplasmic part (GO:0044444)  organelle (GO:0043226)  cytoplasm (GO:0005737)  intracellular part (GO:0044424)  neuron part (GO:0097458)  intracellular (GO:0005622)  neuron projection (GO:0043005)  intracellular organelle (GO:0043229)  cell projection (GO:0042995)  vesicle (GO:0031982)  membrane-bounded organelle (GO:0043227)  membrane-bounded vesicle (GO:0031988)  cell part (GO:0044464)  cell (GO:0005623)  SNARE complex (GO:0031201)  extracellular organelle (GO:0043230)  extracellular vesicular exosome (GO:0070062)  extracellular membrane-bounded organelle (GO:0065010)  extracellular region part (GO:0044421)  cytosol (GO:0005829)  intracellular membrane-bounded organelle (GO:0043231)  intracellular organelle part (GO:0044446)  extracellular region (GO:0005576)  organelle part (GO:0044422) |
| **ASNS, ATP2A2, CHGB, CSE1L, CUTC, KARS, MGLL, NEFL, NEFM, NR4A2, RAN, SIRT4, SLC1A3, SLC25A3, SLC25A4, SLC25A5, SLC25A6, STRN4, TMEM62, TNF, TNFRSF1B, TRADD** | regulation of insulin secretion (GO:0050796)  regulation of peptide hormone secretion (GO:0090276)  regulation of peptide secretion (GO:0002791)  regulation of hormone secretion (GO:0046883)  regulation of peptide transport (GO:0090087)  tumor necrosis factor-mediated signaling pathway (GO:0033209)  adenine transport (GO:0015853)  purine nucleobase transport (GO:0006863)  death (GO:0016265)  single-organism transport (GO:0044765)  nucleobase transport (GO:0015851)  single-organism localization (GO:1902578)  positive regulation of hair follicle development (GO:0051798)  glutamine family amino acid metabolic process (GO:0009064)  cellular amino acid metabolic process (GO:0006520)  small molecule metabolic process (GO:0044281)  regulation of hair follicle development (GO:0051797)  positive regulation of membrane protein ectodomain proteolysis (GO:0051044)  cellular response to tumor necrosis factor (GO:0071356)  transport (GO:0006810)  regulation of secretion (GO:0051046)  cell death (GO:0008219)  carboxylic acid metabolic process (GO:0019752)  regulation of membrane protein ectodomain proteolysis (GO:0051043)  establishment of localization (GO:0051234)  organic acid biosynthetic process (GO:0016053)  carboxylic acid biosynthetic process (GO:0046394)  localization (GO:0051179)  response to tumor necrosis factor (GO:0034612)  regulation of hair cycle (GO:0042634)  cellular amino acid biosynthetic process (GO:0008652)  single-organism intracellular transport (GO:1902582)  oxoacid metabolic process (GO:0043436)  glutamine metabolic process (GO:0006541) | adenine transmembrane transporter activity (GO:0015207)  nucleobase-containing compound transmembrane transporter activity (GO:0015932)  purine nucleobase transmembrane transporter activity (GO:0005345)  protein binding (GO:0005515)  nucleobase transmembrane transporter activity (GO:0015205)  substrate-specific transporter activity (GO:0022892)  transporter activity (GO:0005215)  substrate-specific transmembrane transporter activity (GO:0022891)  binding (GO:0005488)  transmembrane transporter activity (GO:0022857)  active transmembrane transporter activity (GO:0022804)  tumor necrosis factor receptor binding (GO:0005164) | cytoplasm (GO:0005737)  mitochondrial part (GO:0044429)  cytoplasmic part (GO:0044444)  mitochondrial inner membrane (GO:0005743) |
| **ALCAM, CCT5, CLTCL1, EGFR, HIST3H3, IMMT, KDM1A, NDUFB9, RBBP6, TNFRSF1A, TPT1, UBA52** | regulation of response to DNA damage stimulus (GO:2001020)  cellular response to estradiol stimulus (GO:0071392)  regulation of response to stress (GO:0080134)  negative regulation of intrinsic apoptotic signaling pathway in response to DNA damage (GO:1902230)  cellular response to estrogen stimulus (GO:0071391)  negative regulation of response to stimulus (GO:0048585)  regulation of intrinsic apoptotic signaling pathway in response to DNA damage (GO:1902229)  negative regulation of response to DNA damage stimulus (GO:2001021)  negative regulation of epidermal growth factor receptor signaling pathway (GO:0042059)  negative regulation of ERBB signaling pathway (GO:1901185)  positive regulation of defense response (GO:0031349) | protein binding (GO:0005515) | clathrin coat of coated pit (GO:0030132)  intracellular organelle part (GO:0044446)  organelle part (GO:0044422)  clathrin vesicle coat (GO:0030125)  macromolecular complex (GO:0032991)  protein complex (GO:0043234)  vesicle coat (GO:0030120)  membrane-bounded organelle (GO:0043227)  clathrin coat (GO:0030118) |
| **ARIH2, ENO2, ENSA, HK1, HSF1, LYPLA2, MINK1, RAP2A, RBCK1, SCAMP1, SCMH1, UBE2L3, UBE2N, UBE2S, UBE3A** | protein polyubiquitination (GO:0000209)  ubiquitin-dependent protein catabolic process (GO:0006511)  modification-dependent protein catabolic process (GO:0019941)  modification-dependent macromolecule catabolic process (GO:0043632)  proteolysis involved in cellular protein catabolic process (GO:0051603)  cellular protein catabolic process (GO:0044257)  protein catabolic process (GO:0030163)  organic substance catabolic process (GO:1901575)  protein ubiquitination (GO:0016567)  protein K63-linked ubiquitination (GO:0070534)  protein modification by small protein conjugation (GO:0032446)  catabolic process (GO:0009056)  protein modification by small protein conjugation or removal (GO:0070647)  cellular macromolecule catabolic process (GO:0044265)  macromolecule catabolic process (GO:0009057)  proteolysis (GO:0006508)  cellular protein modification process (GO:0006464)  protein modification process (GO:0036211)  cellular metabolic process (GO:0044237)  macromolecule modification (GO:0043412)  primary metabolic process (GO:0044238)  cellular catabolic process (GO:0044248)  organic substance metabolic process (GO:0071704)  protein K11-linked ubiquitination (GO:0070979)  cellular protein metabolic process (GO:0044267)  metabolic process (GO:0008152)  single-organism process (GO:0044699)  organelle organization (GO:0006996)  protein K48-linked ubiquitination (GO:0070936) | ubiquitin-protein transferase activity (GO:0004842)  ligase activity (GO:0016874)  transferase activity (GO:0016740)  acid-amino acid ligase activity (GO:0016881)  catalytic activity (GO:0003824)  ligase activity, forming carbon-nitrogen bonds (GO:0016879)  purine ribonucleoside triphosphate binding (GO:0035639)  purine ribonucleoside binding (GO:0032550)  purine nucleoside binding (GO:0001883)  ribonucleoside binding (GO:0032549)  nucleoside binding (GO:0001882)  purine ribonucleotide binding (GO:0032555)  ribonucleotide binding (GO:0032553)  purine nucleotide binding (GO:0017076) | ubiquitin ligase complex (GO:0000151)  catalytic complex (GO:1902494)  transferase complex (GO:1990234)  intracellular part (GO:0044424)  intracellular (GO:0005622)  membrane-bounded organelle (GO:0043227) |
| **BIN1, CNBP, COBRA1, CUX1, DLGAP4, DNM1, FBP1, FLT3LG, HSPA8, HSPA9, MYST2, PACSIN1, PFN2, PPID, PPP1R1B, SEPT5** | vesicle-mediated transport (GO:0016192)  regulation of vesicle-mediated transport (GO:0060627)  single-organism process (GO:0044699)  regulation of cellular component organization (GO:0051128)  protein folding (GO:0006457)  chaperone-mediated protein folding (GO:0061077)  transport (GO:0006810)  establishment of localization (GO:0051234)  negative regulation of biological process (GO:0048519)  negative regulation of fibril organization (GO:1902904)  regulation of fibril organization (GO:1902903)  positive regulation of cellular component organization (GO:0051130) | protein binding (GO:0005515)  binding (GO:0005488)  anion binding (GO:0043168)  purine nucleotide binding (GO:0017076) | axon terminus (GO:0043679)  neuron projection terminus (GO:0044306)  axon part (GO:0033267)  synapse (GO:0045202)  axon (GO:0030424)  neuron part (GO:0097458)  terminal bouton (GO:0043195)  cell projection part (GO:0044463)  lipid tube (GO:0060987) |

**Table 10**: Subnetwork clustering analysis of the D2 PPI network of the META list. Analysis performed using Webgestalt platform (<http://bioinfo.vanderbilt.edu/webgestalt/>) and Reactome (http://www.reactome.org/).

| **Cluster** | **KEGG** | **Wikipathway** | **Pathway commons** | **Reactome** |
| --- | --- | --- | --- | --- |
| **CYC1, ERCC8, HSPA1L, MACF1, MARCKS, OLA1, OPTN, PLXND1, PRMT5, PSMA2, PSMA3, PSMA5, SGK1, SIP1, SIRT3, SNRPD1, SNRPF, UQCRC1, UQCRH, UQCRQ** | Parkinson's disease  Oxidative phosphorylation  Proteasome  Alzheimer's disease  Huntington's disease  Spliceosome | Proteasome Degradation  Electron Transport Chain  mRNA processing | Metabolism  Metabolism of RNA  Respiratory electron transport  Respiratory electron transport, ATP synthesis by chemiosmotic coupling, and heat production by uncoupling proteins.  The citric acid (TCA) cycle and respiratory electron transport  G1/S DNA Damage Checkpoints  CDT1 association with the CDC6:ORC:origin complex  Signaling by Wnt  APC/C:Cdc20 mediated degradation of Securin  p53-Independent DNA Damage Response  Regulation of Apoptosis  Autodegradation of Cdh1 by Cdh1:APC/C  p53-Dependent G1/S DNA damage checkpoint  Ubiquitin-dependent degradation of Cyclin D  Degradation of beta-catenin by the destruction complex  Ubiquitin Mediated Degradation of Phosphorylated Cdc25A  Vif-mediated degradation of APOBEC3G  Destabilization of mRNA by AUF1 (hnRNP D0)  Ubiquitin-dependent degradation of Cyclin D1  Vpu mediated degradation of CD4  p53-Dependent G1 DNA Damage Response  p53-Independent G1/S DNA damage checkpoint  Metabolism of non-coding RNA  Cdc20:Phospho-APC/C mediated degradation of Cyclin A  SCF-beta-TrCP mediated degradation of Emi1  Stabilization of p53  SCF(Skp2)-mediated degradation of p27/p21  Regulation of activated PAK-2p34 by proteasome mediated degradation  snRNP Assembly  Antigen processing: Ubiquitination & Proteasome degradation  CDK-mediated phosphorylation and removal of Cdc6  APC/C:Cdh1 mediated degradation of Cdc20 and other APC/C:Cdh1 targeted proteins in late mitosis/early G1  Regulation of ornithine decarboxylase (ODC)  Cross-presentation of soluble exogenous antigens (endosomes)  APC/C:Cdc20 mediated degradation of mitotic proteins  Autodegradation of the E3 ubiquitin ligase COP1  Activation of APC/C and APC/C:Cdc20 mediated degradation of mitotic proteins  Removal of licensing factors from origins  Assembly of the pre-replicative complex  Antigen processing-Cross presentation  Orc1 removal from chromatin  Switching of origins to a post-replicative state  DNA Replication Pre-Initiation  Cyclin A:Cdk2-associated events at S phase entry  Synthesis of DNA  Class I MHC mediated antigen processing & presentation  APC/C-mediated degradation of cell cycle proteins  Cyclin E associated events during G1/S transition  Regulation of mRNA Stability by Proteins that Bind AU-rich Elements  Regulation of APC/C activators between G1/S and early anaphase  Regulation of DNA replication  Regulation of mitotic cell cycle  M/G1 Transition  ER-Phagosome pathway  G1/S Transition  Cell Cycle Checkpoints  S Phase  Mitotic G1-G1/S phases  Host Interactions of HIV factors  Apoptosis  mRNA Splicing - Minor Pathway  Metabolism of amino acids and derivatives  HIV Infection  Metabolism of mRNA  Adaptive Immune System  Mitotic M-M/G1 phases  DNA Replication  Cell Cycle, Mitotic  mRNA Splicing - Major Pathway  mRNA Splicing  Signaling events mediated by HDAC Class I | Attenuation phase  HSF1-dependent transactivation  Regulation of HSF1-mediated heat shock response  snRNP Assembly  Metabolism of non-coding RNA  Cellular response to heat stress  HSF1 activation  Respiratory electron transport  AUF1 (hnRNP D0) destabilizes mRNA  Respiratory electron transport, ATP synthesis by chemiosmotic coupling, and heat production by uncoupling proteins.  Cellular responses to stress  Regulation of mRNA stability by proteins that bind AU-rich elements  mRNA Splicing - Minor Pathway  The citric acid (TCA) cycle and respiratory electron transport  Regulation of activated PAK-2p34 by proteasome mediated degradation  Regulation of ornithine decarboxylase (ODC)  CDK-mediated phosphorylation and removal of Cdc6  Ubiquitin-dependent degradation of Cyclin D  Ubiquitin-dependent degradation of Cyclin D1  Vpu mediated degradation of CD4  Autodegradation of the E3 ubiquitin ligase COP1  SCF-beta-TrCP mediated degradation of Emi1  Ubiquitin Mediated Degradation of Phosphorylated Cdc25A  p53-Independent DNA Damage Response  p53-Independent G1/S DNA damage checkpoint  Stabilization of p53  Vif-mediated degradation of APOBEC3G  degradation of DVL  degradation of AXIN  CDT1 association with the CDC6:ORC:origin complex  SCF(Skp2)-mediated degradation of p27/p21  Autodegradation of Cdh1 by Cdh1:APC/C  p53-Dependent G1 DNA Damage Response  p53-Dependent G1/S DNA damage checkpoint  Degradation of GLI1 by the proteasome  GLI3 is processed to GLI3R by the proteasome  Degradation of GLI2 by the proteasome  Hh ligand biogenesis disease  Regulation of Apoptosis  G1/S DNA Damage Checkpoints  APC/C:Cdc20 mediated degradation of Securin  Asymmetric localization of PCP proteins  Assembly of the pre-replicative complex  APC/C:Cdh1 mediated degradation of Cdc20 and other APC/C:Cdh1 targeted proteins in late mitosis/early G1  Cross-presentation of soluble exogenous antigens (endosomes)  APC/C:Cdc20 mediated degradation of mitotic proteins  Cyclin E associated events during G1/S transition  Activation of APC/C and APC/C:Cdc20 mediated degradation of mitotic proteins  Cyclin A:Cdk2-associated events at S phase entry  Activation of NF-kappaB in B cells  Processing-defective Hh variants abrogate ligand secretion  Transcriptional activation of mitochondrial biogenesis  Hedgehog ligand biogenesis  Orc1 removal from chromatin  Switching of origins to a post-replicative state  Removal of licensing factors from origins  Regulation of DNA replication  Regulation of APC/C activators between G1/S and early anaphase  Apoptosis  Mitochondrial biogenesis  M/G1 Transition  DNA Replication Pre-Initiation  Regulation of mitotic cell cycle  APC/C-mediated degradation of cell cycle proteins  Degradation of beta-catenin by the destruction complex  S45 mutants of beta-catenin aren't phosphorylated  T41 mutants of beta-catenin aren't phosphorylated  deletions in the AXIN genes in hepatocellular carcinoma result in elevated WNT signaling  APC truncation mutants are not K63 polyubiquitinated  truncations of AMER1 destabilize the destruction complex  S33 mutants of beta-catenin aren't phosphorylated  AXIN missense mutants destabilize the destruction complex  APC truncation mutants have impaired AXIN binding  TCF7L2 mutants don't bind CTBP  misspliced GSK3beta mutants stabilize beta-catenin  S37 mutants of beta-catenin aren't phosphorylated  deletions in the AMER1 gene destabilize the destruction complex  AMER1 mutants destabilize the destruction complex  truncated APC mutants destabilize the destruction complex  AXIN mutants destabilize the destruction complex, activating WNT signaling  phosphorylation site mutants of CTNNB1 are not targeted to the proteasome by the destruction complex  PCP/CE pathway  Synthesis of DNA  G1/S Transition  Release of apoptotic factors from the mitochondria  DNA Replication  mRNA Splicing  mRNA Splicing - Major Pathway  Hedgehog 'off' state  Cell Cycle Checkpoints  Processing of Capped Intron-Containing Pre-mRNA |
| **AHSG, AKR1B1, BCAS1, CAMKK2, CDC42, CEP63, DYNLL1, ELF3, ETFA, FTSJ1, FUBP1, GDI1, GSTM3, HIST1H2AB, MPG, MSN, RBL1, RUVBL2, SERBP1, SMN1, SNCG, SNRNP70, SRCAP, TFE3, TSPAN7, VIM** |  |  | Signaling events mediated by VEGFR1 and VEGFR2  Regulation of nuclear SMAD2/3 signaling  Plasma membrane estrogen receptor signaling  VEGF and VEGFR signaling network  Regulation of cytoplasmic and nuclear SMAD2/3 signaling  AP-1 transcription factor network  Regulation of CDC42 activity  ALK1 pathway  CDC42 signaling events  ALK1 signaling events  TGF-beta receptor signaling  Intrinsic Pathway for Apoptosis  TRAIL signaling pathway  Regulation of RAC1 activity  Golgi Cisternae Pericentriolar Stack Reorganization  Recruitment of mitotic centrosome proteins and complexes  Nectin adhesion pathway  PAR1-mediated thrombin signaling events  BMP receptor signaling  PDGFR-beta signaling pathway  Glypican 1 network  E2F transcription factor network  RhoA signaling pathway  Signaling events mediated by Hepatocyte Growth Factor Receptor (c-Met)  EGF receptor (ErbB1) signaling pathway  PDGF receptor signaling network  Class I PI3K signaling events  IGF1 pathway  Centrosome maturation  Syndecan-1-mediated signaling events  RAC1 signaling pathway  IL3-mediated signaling events  Insulin Pathway  IFN-gamma pathway  Regulation of RhoA activity  mTOR signaling pathway  Alpha9 beta1 integrin signaling events  EGFR-dependent Endothelin signaling events  Signaling events mediated by focal adhesion kinase  S1P1 pathway  Loss of proteins required for interphase microtubule organizationÃ‚Â from the centrosome  Urokinase-type plasminogen activator (uPA) and uPAR-mediated signaling  IL5-mediated signaling events  Arf6 trafficking events  Arf6 downstream pathway  Arf6 signaling events  ErbB receptor signaling network  Loss of Nlp from mitotic centrosomes  Class I PI3K signaling events mediated by Akt  LKB1 signaling events  Integrin-linked kinase signaling  Thrombin/protease-activated receptor (PAR) pathway  ErbB1 downstream signaling  GMCSF-mediated signaling events  Internalization of ErbB1  Mitotic Prophase  Sphingosine 1-phosphate (S1P) pathway  Endothelins  Proteoglycan syndecan-mediated signaling events  Glypican pathway  TNF receptor signaling pathway  Beta1 integrin cell surface interactions  G2/M Transition  Integrin family cell surface interactions  Mitotic G2-G2/M phases  Cell Cycle, Mitotic | Glutathione conjugation  Phase II conjugation |
| **ACTR6, CS, DHRS2, EEF1A2, EEF1B2, HSPB1, MAP3K8, PFKL, PHB2, PTPLAD1, SERINC3, TAB2, TAGLN2, TUBA3D, USP1, VDAC2** | MAPK signaling pathway  Toll-like receptor signaling pathway | MAPK signaling pathway  Translation Factors  Toll-like receptor signaling pathway  TNF alpha Signaling Pathway  Insulin Signaling  Lymphocyte TarBase  Leukocyte TarBase  Regulation of toll-like receptor signaling pathway | TNF alpha/NF-kB  p38 MAPK signaling pathway  Interleukin-1 signaling  ALK1 pathway  Regulation of nuclear SMAD2/3 signaling  Integrin-linked kinase signaling  IL1-mediated signaling events  Regulation of cytoplasmic and nuclear SMAD2/3 signaling  TNF receptor signaling pathway  AP-1 transcription factor network  TGF-beta receptor signaling  BMP receptor signaling  ALK1 signaling events  CDC42 signaling events  Signaling by Interleukins  Regulation of CDC42 activity |  |
| **AP2A1, AP2A2, AP2S1, ARHGEF7, ATP2B2, CCDC50, CUL3, EPS15, GABBR2, GRHPR, HSP90AA1, LASS2, LSM1, NARS, OTUD7B, PRKCD, RPS3A, SHC1, SLC9A3R2, TNFRSF10A, TOE1** | Endocytosis  Huntington's disease  Natural killer cell mediated cytotoxicity  Neurotrophin signaling pathway | EGF-EGFR Signaling Pathway  Synaptic Vesicle Pathway  Alpha 6 Beta 4 signaling pathway  Lymphocyte TarBase  IL-6 signaling pathway  AGE-RAGE pathway  Oncostatin M Signaling Pathway  TNF alpha Signaling Pathway  TCR Signaling Pathway  Integrin-mediated cell adhesion  B Cell Receptor Signaling Pathway  Corticotropin-releasing hormone | Signaling by EGFR  EGFR downregulation  Nef Mediated CD8 Down-regulation  Nef Mediated CD4 Down-regulation  Retrograde neurotrophin signalling  Thrombin/protease-activated receptor (PAR) pathway  ErbB1 downstream signaling  Arf6 signaling events  PDGF receptor signaling network  Endothelins  mTOR signaling pathway  Internalization of ErbB1  PDGFR-beta signaling pathway  Nectin adhesion pathway  IGF1 pathway  EGFR-dependent Endothelin signaling events  VEGF and VEGFR signaling network  Plasma membrane estrogen receptor signaling  Insulin Pathway  LKB1 signaling events  PAR1-mediated thrombin signaling events  Class I PI3K signaling events  S1P1 pathway  Arf6 trafficking events  Class I PI3K signaling events mediated by Akt  Arf6 downstream pathway  Glypican 1 network  Alpha9 beta1 integrin signaling events  Signaling events mediated by focal adhesion kinase  Sphingosine 1-phosphate (S1P) pathway  ErbB receptor signaling network  IL5-mediated signaling events  IFN-gamma pathway  Nef-mediates down modulation of cell surface receptors by recruiting them to clathrin adapters  EGF receptor (ErbB1) signaling pathway  IL3-mediated signaling events  Syndecan-1-mediated signaling events  Signaling events mediated by Hepatocyte Growth Factor Receptor (c-Met)  Urokinase-type plasminogen activator (uPA) and uPAR-mediated signaling  GMCSF-mediated signaling events  Signaling events mediated by VEGFR1 and VEGFR2  TRAIL signaling pathway  Glypican pathway  Proteoglycan syndecan-mediated signaling events  Beta1 integrin cell surface interactions  Integrin family cell surface interactions  The role of Nef in HIV-1 replication and disease pathogenesis  NGF signalling via TRKA from the plasma membrane  Recycling pathway of L1  Signal transduction by L1  Signalling by NGF  Signal Transduction  Developmental Biology  L1CAM interactions  Axon guidance  Platelet activation, signaling and aggregation  Hemostasis  Integrin-linked kinase signaling  Host Interactions of HIV factors  Regulation of CDC42 activity  CDC42 signaling events  Alpha6Beta4Integrin  Metabolism  HIV Infection  KitReceptor  Metabolism of mRNA  Trk receptor signaling mediated by PI3K and PLC-gamma  Downstream signaling of activated FGFR  IL2 signaling events mediated by PI3K  Metabolism of RNA  Syndecan-2-mediated signaling events  AP-1 transcription factor network  Neurotransmitter Receptor Binding And Downstream Transmission In The Postsynaptic Cell  Signaling by FGFR  Signaling by Aurora kinases  Influenza Viral RNA Transcription and Replication  LPA receptor mediated events  Neurotrophic factor-mediated Trk receptor signaling  IL2-mediated signaling events  Transmission across Chemical Synapses  Influenza Life Cycle | EGFR downregulation  Signaling by EGFR in Cancer  Nef Mediated CD8 Down-regulation  SHC-related events triggered by IGF1R  Signaling by EGFR  Nef Mediated CD4 Down-regulation  Reduction of cytosolic Ca++ levels  Retrograde neurotrophin signalling  WNT5A-dependent internalization of FZD4  EPH-Ephrin signaling  Trafficking of GluR2-containing AMPA receptors  Platelet calcium homeostasis  Nef-mediates down modulation of cell surface receptors by recruiting them to clathrin adapters  The role of Nef in HIV-1 replication and disease pathogenesis  Trafficking of AMPA receptors  Glutamate Binding, Activation of AMPA Receptors and Synaptic Plasticity  Recycling pathway of L1  IGF1R signaling cascade  Signaling by Type 1 Insulin-like Growth Factor 1 Receptor (IGF1R)  Ion transport by P-type ATPases  Signalling by NGF  EPH-ephrin mediated repulsion of cells  HSF1 activation |
| **ACTBL2, ARRB2, CHEK2, CLTB, CRMP1, DLG5, DLST, HNRNPH1, HNRNPH2, NDRG1, NSUN2, PFN1, PKM, PPP2CB, PPP2R2A, PPP2R5B, PPP2R5C, PPP2R5D, PPP2R5E, RPS12, SAG, SGOL2, TOB1** | mRNA surveillance pathway  Oocyte meiosis  Wnt signaling pathway | Glycogen Metabolism  Wnt Signaling Pathway and Pluripotency  Diurnally regulated genes with circadian orthologs  Wnt Signaling Pathway  DNA damage response (only ATM dependent) | TRAIL signaling pathway  Signaling events mediated by focal adhesion kinase  Thrombin/protease-activated receptor (PAR) pathway  Arf6 downstream pathway  Beta1 integrin cell surface interactions  PAR1-mediated thrombin signaling events  Class I PI3K signaling events  Proteoglycan syndecan-mediated signaling events  Alpha9 beta1 integrin signaling events  S1P1 pathway  VEGF and VEGFR signaling network  IL5-mediated signaling events  Insulin Pathway  Syndecan-1-mediated signaling events  IL3-mediated signaling events  ErbB receptor signaling network  LKB1 signaling events  Polo-like kinase signaling events in the cell cycle  IFN-gamma pathway  Nectin adhesion pathway  GMCSF-mediated signaling events  PDGFR-beta signaling pathway  Sphingosine 1-phosphate (S1P) pathway  Class I PI3K signaling events mediated by Akt  Endothelins  Internalization of ErbB1  PDGF receptor signaling network  Urokinase-type plasminogen activator (uPA) and uPAR-mediated signaling  Signaling events mediated by VEGFR1 and VEGFR2  Signaling events mediated by Hepatocyte Growth Factor Receptor (c-Met)  ErbB1 downstream signaling  Arf6 signaling events  EGF receptor (ErbB1) signaling pathway  EGFR-dependent Endothelin signaling events  IGF1 pathway  Signaling by Aurora kinases  Glypican 1 network  Plasma membrane estrogen receptor signaling  Glypican pathway  Arf6 trafficking events  mTOR signaling pathway  AP-1 transcription factor network  Integrin family cell surface interactions  Integrin-linked kinase signaling  Aurora B signaling  CDC42 signaling events  Regulation of CDC42 activity  Aurora A signaling  HIF-1-alpha transcription factor network  Hypoxic and oxygen homeostasis regulation of HIF-1-alpha  Wnt  TGF-beta receptor signaling  Regulation of cytoplasmic and nuclear SMAD2/3 signaling  Regulation of nuclear SMAD2/3 signaling  ALK1 signaling events  ALK1 pathway  mRNA Splicing - Major Pathway  PLK1 signaling events  mRNA Splicing  Nonsense Mediated Decay Enhanced by the Exon Junction Complex  Nonsense-Mediated Decay  IL2-mediated signaling events | Beta-catenin phosphorylation cascade  Platelet sensitization by LDL  CTLA4 inhibitory signaling  disassembly of the destruction complex and recruitment of AXIN to the membrane  Glycolysis  Resolution of Sister Chromatid Cohesion  Mitotic Prometaphase  Mitotic Anaphase  Mitotic Metaphase and Anaphase  Platelet homeostasis  phosphorylation site mutants of CTNNB1 are not targeted to the proteasome by the destruction complex  AXIN missense mutants destabilize the destruction complex  misspliced GSK3beta mutants stabilize beta-catenin  S45 mutants of beta-catenin aren't phosphorylated  T41 mutants of beta-catenin aren't phosphorylated  S33 mutants of beta-catenin aren't phosphorylated  APC truncation mutants have impaired AXIN binding  S37 mutants of beta-catenin aren't phosphorylated  truncations of AMER1 destabilize the destruction complex  AXIN mutants destabilize the destruction complex, activating WNT signaling  AMER1 mutants destabilize the destruction complex  Degradation of beta-catenin by the destruction complex  deletions in the AMER1 gene destabilize the destruction complex  deletions in the AXIN genes in hepatocellular carcinoma result in elevated WNT signaling  truncated APC mutants destabilize the destruction complex  APC truncation mutants are not K63 polyubiquitinated  TCF7L2 mutants don't bind CTBP  Signaling by Wnt  Costimulation by the CD28 family  Separation of Sister Chromatids  PP2A-mediated dephosphorylation of key metabolic factors  Glucose metabolism  M Phase  Formation of annular gap junctions  Gap junction degradation  ERKs are inactivated  TCF dependent signaling in response to WNT  misspliced LRP5 mutants have enhanced beta-catenin-dependent signaling  RNF mutants show enhanced WNT signaling and proliferation  XAV939 inhibits tankyrase, stabilizing AXIN  Hemostasis  ERK/MAPK targets  Cell Cycle  Cyclin A/B1 associated events during G2/M transition  DARPP-32 events  Nuclear Events (kinase and transcription factor activation)  Signaling by WNT in cancer  Cell Cycle, Mitotic  MAPK targets/ Nuclear events mediated by MAP kinases  Cyclin D associated events in G1  G1 Phase  Gap junction trafficking  Gap junction trafficking and regulation  MASTL Facilitates Mitotic Progression  EPH-ephrin mediated repulsion of cells  Inhibition of replication initiation of damaged DNA by RB1/E2F1  Spry regulation of FGF signaling  Initiation of Nuclear Envelope Reformation  Nuclear Envelope Reassembly  WNT5A-dependent internalization of FZD4  MAP kinase activation in TLR cascade  Cell-extracellular matrix interactions  Metabolism of carbohydrates  Myoclonic epilepsy of Lafora  Glycogen storage diseases  Opioid Signalling  TRAF6 mediated induction of NFkB and MAP kinases upon TLR7/8 or 9 activation  MyD88 cascade initiated on plasma membrane  Toll Like Receptor 5 (TLR5) Cascade  Toll Like Receptor 10 (TLR10) Cascade  MyD88 dependent cascade initiated on endosome  Toll Like Receptor 7/8 (TLR7/8) Cascade  Disease  PCP/CE pathway  Toll Like Receptor 9 (TLR9) Cascade  EPH-Ephrin signaling  MyD88:Mal cascade initiated on plasma membrane  Toll Like Receptor TLR6:TLR2 Cascade  Toll Like Receptor TLR1:TLR2 Cascade  Toll Like Receptor 2 (TLR2) Cascade  TRIF-mediated TLR3/TLR4 signaling  MyD88-independent cascade  Toll Like Receptor 3 (TLR3) Cascade  Interaction between L1 and Ankyrins  Adherens junctions interactions  Integration of energy metabolism  E2F mediated regulation of DNA replication  Nonsense-Mediated Decay (NMD)  Nonsense Mediated Decay (NMD) enhanced by the Exon Junction Complex (EJC)  Axon guidance  mRNA Splicing - Major Pathway  mRNA Splicing  Activated TLR4 signalling  Processing of Capped Intron-Containing Pre-mRNA  G2/M Transition  Recycling pathway of L1  EPHB-mediated forward signaling  Mitotic G2-G2/M phases |
| **ACTR3B, ARHGEF1, ARPC5, ARPC5L, ATP5A1, ATP5B, ATP5C1, ATP5F1, ATP5H, CCT2, CCT7, DDT, DSTYK, EIF3G, EIF3H, GNA13, MTPN, NGEF, PPP2R2D, PRAF2, RHOB, USP3** | Oxidative phosphorylation  Parkinson's disease  Alzheimer's disease  Huntington's disease  Regulation of actin cytoskeleton  Pathogenic Escherichia coli infection  Shigellosis  Bacterial invasion of epithelial cells  Fc gamma R-mediated phagocytosis  Metabolic pathways  Vascular smooth muscle contraction | Electron Transport Chain  Oxidative phosphorylation  Regulation of Actin Cytoskeleton  G13 Signaling Pathway  Translation Factors  Pathogenic Escherichia coli infection  G Protein Signaling Pathways  Epithelium TarBase | Formation of ATP by chemiosmotic coupling  Respiratory electron transport, ATP synthesis by chemiosmotic coupling, and heat production by uncoupling proteins.  The citric acid (TCA) cycle and respiratory electron transport  G alpha (12/13) signalling events  Folding of actin by CCT/TriC  Formation of tubulin folding intermediates by CCT/TriC  Association of TriC/CCT with target proteins during biosynthesis  Cooperation of Prefoldin and TriC/CCT in actin and tubulin folding  Chaperonin-mediated protein folding  Prefoldin mediated transfer of substrate to CCT/TriC  Metabolism of proteins  Protein folding  Regulation of RAC1 activity  RhoA signaling pathway  Translation initiation complex formation  Ribosomal scanning and start codon recognition  Regulation of RhoA activity  RAC1 signaling pathway  Metabolism  Formation of the ternary complex, and subsequently, the 43S complex  Thromboxane A2 receptor signaling  Activation of the mRNA upon binding of the cap-binding complex and eIFs, and subsequent binding to 43S | Formation of ATP by chemiosmotic coupling  G alpha (12/13) signalling events  Respiratory electron transport, ATP synthesis by chemiosmotic coupling, and heat production by uncoupling proteins.  The citric acid (TCA) cycle and respiratory electron transport  Folding of actin by CCT/TriC |
| **ATP12A, C21ORF33, CAMK2A, CAMK2B, CHMP5, CLTA, CPLX1, CPSF2, CTNNB1, EEF1D, FRA10AC1, GLRX3, HSP90B1, INPP5D, MME, NDUFB4, PIP4K2B, RBM39, RNPS1, RXRB, STAMBP** | Melanogenesis  Thyroid cancer  Wnt signaling pathway  Endocytosis  Prostate cancer  mRNA surveillance pathway  Glioma  Long-term potentiation  ErbB signaling pathway  Phosphatidylinositol signaling system  Gastric acid secretion  Bacterial invasion of epithelial cells  Pathways in cancer  GnRH signaling pathway  Oocyte meiosis  Neurotrophin signaling pathway  Oxidative phosphorylation | EGF-EGFR Signaling Pathway  mRNA processing  Synaptic Vesicle Pathway | Unblocking of NMDA receptor, glutamate binding and activation  CREB phosphorylation through the activation of CaMKII  Glypican 1 network  Ras activation uopn Ca2+ infux through NMDA receptor  Transmission across Chemical Synapses  LKB1 signaling events  S1P1 pathway  PDGF receptor signaling network  ErbB receptor signaling network  Signaling events mediated by focal adhesion kinase  Signaling events mediated by Hepatocyte Growth Factor Receptor (c-Met)  Proteoglycan syndecan-mediated signaling events  ErbB1 downstream signaling  Thrombin/protease-activated receptor (PAR) pathway  TRAIL signaling pathway  PDGFR-beta signaling pathway  VEGF and VEGFR signaling network  Signaling events mediated by VEGFR1 and VEGFR2  Endothelins  IFN-gamma pathway  Arf6 trafficking events  Beta1 integrin cell surface interactions  Trafficking of AMPA receptors  Arf6 signaling events  Wnt  Insulin Pathway  Internalization of ErbB1  IGF1 pathway  Class I PI3K signaling events  Arf6 downstream pathway  IL3-mediated signaling events  Sphingosine 1-phosphate (S1P) pathway  Alpha9 beta1 integrin signaling events  IL5-mediated signaling events  Nectin adhesion pathway  Glypican pathway  Glutamate Binding, Activation of AMPA Receptors and Synaptic Plasticity  Plasma membrane estrogen receptor signaling  EGFR-dependent Endothelin signaling events  EGF receptor (ErbB1) signaling pathway  Urokinase-type plasminogen activator (uPA) and uPAR-mediated signaling  GMCSF-mediated signaling events  mTOR signaling pathway  Syndecan-1-mediated signaling events  PAR1-mediated thrombin signaling events  Class I PI3K signaling events mediated by Akt  Integrin family cell surface interactions  CREB phosphorylation through the activation of Ras  Post NMDA receptor activation events  Post-Elongation Processing of Intron-Containing pre-mRNA  mRNA 3'-end processing  Activation of NMDA receptor upon glutamate binding and postsynaptic events  Cleavage of Growing Transcript in the Termination Region  Post-Elongation Processing of the Transcript  Neuronal System  RNA Polymerase II Transcription Termination  Transport of Mature Transcript to Cytoplasm  AP-1 transcription factor network  Regulation of nuclear SMAD2/3 signaling  TGF-beta receptor signaling  Regulation of cytoplasmic and nuclear SMAD2/3 signaling  Membrane Trafficking  Integrin-linked kinase signaling  Neurotransmitter Receptor Binding And Downstream Transmission In The Postsynaptic Cell  ALK1 signaling events  ALK1 pathway  RNA Polymerase II Transcription  mRNA Splicing - Major Pathway  mRNA Splicing  Regulation of Androgen receptor activity  Gene Expression  CDC42 signaling events  Regulation of CDC42 activity  Androgen-mediated signaling  Processing of Capped Intron-Containing Pre-mRNA | CREB phosphorylation through the activation of CaMKII  Ras activation uopn Ca2+ infux through NMDA receptor  Unblocking of NMDA receptor, glutamate binding and activation  HSF1-dependent transactivation  CREB phosphorylation through the activation of Ras  Trafficking of AMPA receptors  Glutamate Binding, Activation of AMPA Receptors and Synaptic Plasticity  Synthesis of PIPs at the plasma membrane  Post NMDA receptor activation events  Cellular response to heat stress  Activation of NMDA receptor upon glutamate binding and postsynaptic events |
| **ACHE, ACTB, ALDOA, DDX24, DPYSL5, E2F1, ENO1, FHIT, GNAI2, GNB2L1, HSP90AB1, HSPA5, HSPD1, LAP3, MDH2, MRPL10, MYH9, NDUFA13, RPSA, STARD13, TPI1, TRRAP, TUBA1B, TUBA4A, USP22, VDAC3, XRN1** | Glycolysis / Gluconeogenesis  RNA degradation  Pathogenic Escherichia coli infection  Gap junction  Tight junction  Phagosome  Fructose and mannose metabolism  Non-small cell lung cancer  Viral myocarditis  Antigen processing and presentation  Gastric acid secretion  Small cell lung cancer  Progesterone-mediated oocyte maturation  Prostate cancer | Glycolysis and Gluconeogenesis  Parkin-Ubiquitin Proteasomal System pathway  Pathogenic Escherichia coli infection  TSH signaling pathway | Plasma membrane estrogen receptor signaling  sucrose degradation  IGF1 pathway  VEGF and VEGFR signaling network  Hypoxic and oxygen homeostasis regulation of HIF-1-alpha  Semaphorin interactions  Signaling events mediated by VEGFR1 and VEGFR2  Validated targets of C-MYC transcriptional activation  Syndecan-1-mediated signaling events  PDGFR-beta signaling pathway  Glypican 1 network  IL3-mediated signaling events  PDGF receptor signaling network  Platelet degranulation  Endothelins  Alpha9 beta1 integrin signaling events  Arf6 signaling events  Arf6 downstream pathway  Sphingosine 1-phosphate (S1P) pathway  Nectin adhesion pathway  IL5-mediated signaling events  mTOR signaling pathway  C-MYC pathway  Thrombin/protease-activated receptor (PAR) pathway  LKB1 signaling events  GMCSF-mediated signaling events  Class I PI3K signaling events mediated by Akt  Gluconeogenesis  Internalization of ErbB1  EGFR-dependent Endothelin signaling events  Glucose metabolism  ErbB1 downstream signaling  AP-1 transcription factor network  ErbB receptor signaling network  Class I PI3K signaling events  IFN-gamma pathway  EGF receptor (ErbB1) signaling pathway  Urokinase-type plasminogen activator (uPA) and uPAR-mediated signaling  Response to elevated platelet cytosolic Ca2+  PAR1-mediated thrombin signaling events  Signaling events mediated by Hepatocyte Growth Factor Receptor (c-Met)  Insulin Pathway  Signaling events mediated by focal adhesion kinase  Arf6 trafficking events  Platelet activation, signaling and aggregation  S1P1 pathway  Proteoglycan syndecan-mediated signaling events  TRAIL signaling pathway  Glypican pathway  Beta1 integrin cell surface interactions  Integrin-linked kinase signaling  Integrin family cell surface interactions  Developmental Biology  CDC42 signaling events  Axon guidance  Regulation of CDC42 activity  HIF-1-alpha transcription factor network  E2F transcription factor network  Metabolism of carbohydrates  Notch-mediated HES/HEY network  Notch signaling pathway | Cooperation of Prefoldin and TriC/CCT in actin and tubulin folding  Post-chaperonin tubulin folding pathway  Gap junction trafficking  Recycling pathway of L1  Gap junction trafficking and regulation  Formation of tubulin folding intermediates by CCT/TriC  Chaperonin-mediated protein folding  Protein folding  Gluconeogenesis  Microtubule-dependent trafficking of connexons from Golgi to the plasma membrane  Transport of connexons to the plasma membrane  Translocation of GLUT4 to the plasma membrane  Mitotic G2-G2/M phases  Axon guidance  Glycolysis  Recruitment of NuMA to mitotic centrosomes  Glucose metabolism  L1CAM interactions  Gap junction assembly  Centrosome maturation  Recruitment of mitotic centrosome proteins and complexes  Kinesins  Muscle contraction  Developmental Biology  Hedgehog 'off' state  Prefoldin mediated transfer of substrate to CCT/TriC  G2/M Transition  Semaphorin interactions  Membrane Trafficking  Signaling by Hedgehog  G2 Phase  Factors involved in megakaryocyte development and platelet production  EPH-Ephrin signaling  Formation of annular gap junctions  CDC6 association with the ORC:origin complex  Gap junction degradation  Resolution of Sister Chromatid Cohesion |
| **AARS2, AP2B1, AP2M1, ASAP2, CAPZB, CASK, CHM, COPS2, EEF1A1, GDA, ITSN1, MEGF10, MLH1, MPST, MRPL20, SF3A3, SIRPA, SMNDC1, TMSB4X, TRIM2, UBE2E1, UIMC1** |  |  |  | Signal regulatory protein (SIRP) family interactions |
| **BTF3, CCND1, CDC34, CDK6, CTNNBIP1, ESR1, PPP1CB, PPP1CC, RPA3, SH2D4A, SH3GL1, SH3GL2, TCEA2, TIMM13** | Chronic myeloid leukemia  p53 signaling pathway  Melanoma  Glioma  Long-term potentiation  Non-small cell lung cancer  Pancreatic cancer  Focal adhesion  Small cell lung cancer  Vascular smooth muscle contraction  Oocyte meiosis  Cell cycle  Insulin signaling pathway  Wnt signaling pathway  Endocytosis  Regulation of actin cytoskeleton | G1 to S cell cycle control  miRNAs involved in DDR  Wnt Signaling Pathway  miRNA regulation of DNA Damage Response  Signaling Pathways in Glioblastoma  DNA damage response  Leptin signaling pathway  Muscle cell TarBase  Integrated Pancreatic Cancer Pathway | Syndecan-1-mediated signaling events  Internalization of ErbB1  ErbB receptor signaling network  Insulin Pathway  Glypican 1 network  Alpha9 beta1 integrin signaling events  ErbB1 downstream signaling  Class I PI3K signaling events mediated by Akt  IFN-gamma pathway  EGF receptor (ErbB1) signaling pathway  PDGF receptor signaling network  EGFR-dependent Endothelin signaling events  Class I PI3K signaling events  Signaling events mediated by VEGFR1 and VEGFR2  LKB1 signaling events  Arf6 trafficking events  Thrombin/protease-activated receptor (PAR) pathway  Nectin adhesion pathway  Endothelins  Urokinase-type plasminogen activator (uPA) and uPAR-mediated signaling  Arf6 signaling events  Arf6 downstream pathway  VEGF and VEGFR signaling network  PAR1-mediated thrombin signaling events  Signaling events mediated by Hepatocyte Growth Factor Receptor (c-Met)  S1P1 pathway  PDGFR-beta signaling pathway  Sphingosine 1-phosphate (S1P) pathway  IL5-mediated signaling events  mTOR signaling pathway  GMCSF-mediated signaling events  Signaling events mediated by focal adhesion kinase  IGF1 pathway  Plasma membrane estrogen receptor signaling  IL3-mediated signaling events  TRAIL signaling pathway  Glypican pathway  Proteoglycan syndecan-mediated signaling events  Beta1 integrin cell surface interactions  Integrin family cell surface interactions  Hormone-sensitive lipase (HSL)-mediated triacylglycerol hydrolysis  Cell Cycle, Mitotic  Mitotic G1-G1/S phases  Regulation of nuclear beta catenin signaling and target gene transcription  Regulation of CDC42 activity  TGFBR  CDC42 signaling events  Canonical Wnt signaling pathway  Noncanonical Wnt signaling pathway  Cyclin D associated events in G1  G1 Phase  Regulation of RhoA activity  Wnt signaling network  RhoA signaling pathway  Regulation of RAC1 activity  Glypican 3 network  RAC1 signaling pathway  FOXM1 transcription factor network  Syndecan-4-mediated signaling events  Posttranslational regulation of adherens junction stability and dissassembly  Lipid digestion, mobilization, and transport  N-cadherin signaling events  AP-1 transcription factor network  Coregulation of Androgen receptor activity  E-cadherin signaling events  Stabilization and expansion of the E-cadherin adherens junction  Integrin-linked kinase signaling  ATF-2 transcription factor network  E-cadherin signaling in the nascent adherens junction  Validated nuclear estrogen receptor alpha network  Regulation of retinoblastoma protein  Regulation of Telomerase  AndrogenReceptor  C-MYB transcription factor network  Regulation of Androgen receptor activity  Cell Cycle Checkpoints  S Phase  Androgen-mediated signaling  p38 MAPK signaling pathway |  |
| **APP, CAT, CBFB, DBN1, DLGAP5, HSD17B10, HSPA4, HSPA6, IKBKG, MAP2, P4HB, PDIA3, PPIA, PRSS23, PTPRN, PTPRS, RG9MTD1, SCHIP1, SFXN1, SLC8A1, STXBP1, SUPT5H, TAB1, TAP1, TRAF2, TUBB2B, TUFM** | Antigen processing and presentation  Protein processing in endoplasmic reticulum  MAPK signaling pathway  Osteoclast differentiation  Toxoplasmosis  Primary immunodeficiency  NOD-like receptor signaling pathway  Adipocytokine signaling pathway  RIG-I-like receptor signaling pathway  Small cell lung cancer  Apoptosis | RANKL-RANK Signaling Pathway  Parkin-Ubiquitin Proteasomal System pathway  TNF alpha Signaling Pathway  MAPK signaling pathway  IL-1 signaling pathway  Tryptophan metabolism | Innate Immune System  Nucleotide-binding domain, leucine rich repeat containing receptor (NLR) signaling pathways  TRAF6 mediated NF-kB activation  Immune System  IL3-mediated signaling events  TNF receptor signaling pathway  Glypican 1 network  PAR1-mediated thrombin signaling events  Class I PI3K signaling events  ErbB receptor signaling network  PDGFR-beta signaling pathway  Interleukin-1 signaling  GMCSF-mediated signaling events  Antigen Presentation: Folding, assembly and peptide loading of class I MHC  Urokinase-type plasminogen activator (uPA) and uPAR-mediated signaling  Endothelins  Alpha9 beta1 integrin signaling events  EGF receptor (ErbB1) signaling pathway  Thrombin/protease-activated receptor (PAR) pathway  Class I PI3K signaling events mediated by Akt  VEGF and VEGFR signaling network  ErbB1 downstream signaling  EGFR-dependent Endothelin signaling events  Signaling events mediated by Hepatocyte Growth Factor Receptor (c-Met)  Syndecan-1-mediated signaling events  IL5-mediated signaling events  IFN-gamma pathway  LKB1 signaling events  PDGF receptor signaling network  Nectin adhesion pathway  Signaling events mediated by VEGFR1 and VEGFR2  Arf6 signaling events  NOD1/2 Signaling Pathway  Insulin Pathway  IGF1 pathway  Arf6 trafficking events  mTOR signaling pathway  Sphingosine 1-phosphate (S1P) pathway  Glypican pathway  Internalization of ErbB1  S1P1 pathway  Plasma membrane estrogen receptor signaling  Signaling events mediated by focal adhesion kinase  TRAIL signaling pathway  FAS (CD95) signaling pathway  Arf6 downstream pathway  TNF alpha/NF-kB  Proteoglycan syndecan-mediated signaling events  Beta1 integrin cell surface interactions  Integrin family cell surface interactions  TRAF6 Mediated Induction of proinflammatory cytokines  Caspase cascade in apoptosis  NFkB and MAP kinases activation mediated by TLR4 signaling repertoire  Toll Like Receptor 3 (TLR3) Cascade  TRIF mediated TLR3 signaling  CD40/CD40L signaling  Toll Like Receptor 5 (TLR5) Cascade  MyD88 dependent cascade initiated on endosome  MyD88 cascade initiated on plasma membrane  MyD88-independent cascade initiated on plasma membrane  Toll Like Receptor 10 (TLR10) Cascade  TRAF6 mediated induction of NFkB and MAP kinases upon TLR7/8 or 9 activation  Toll Like Receptor 7/8 (TLR7/8) Cascade  MyD88:Mal cascade initiated on plasma membrane  Toll Like Receptor 2 (TLR2) Cascade  Toll Like Receptor 9 (TLR9) Cascade  Toll Like Receptor TLR1:TLR2 Cascade  Aurora A signaling  Toll Like Receptor TLR6:TLR2 Cascade  IL1-mediated signaling events  RIG-I/MDA5 mediated induction of IFN-alpha/beta pathways  Adaptive Immune System  Activated TLR4 signalling  Toll Like Receptor 4 (TLR4) Cascade  ER-Phagosome pathway  Antigen processing-Cross presentation  Toll Receptor Cascades  Signaling by Interleukins  Regulation of cytoplasmic and nuclear SMAD2/3 signaling  Regulation of nuclear SMAD2/3 signaling  Signaling by Aurora kinases  TGF-beta receptor signaling  Class I MHC mediated antigen processing & presentation  HIV Life Cycle  PLK1 signaling events | Microtubule-dependent trafficking of connexons from Golgi to the plasma membrane  Transport of connexons to the plasma membrane  Post-chaperonin tubulin folding pathway  Recruitment of NuMA to mitotic centrosomes  TRAF6 mediated NF-kB activation  Gap junction assembly  TAK1 activates NFkB by phosphorylation and activation of IKKs complex  RIG-I/MDA5 mediated induction of IFN-alpha/beta pathways  Gap junction trafficking  Kinesins  Recycling pathway of L1  Gap junction trafficking and regulation  TRIF-mediated TLR3/TLR4 signaling  MyD88-independent cascade  Toll Like Receptor 3 (TLR3) Cascade  Protein folding  Resolution of Sister Chromatid Cohesion  Nucleotide-binding domain, leucine rich repeat containing receptor (NLR) signaling pathways  JNK (c-Jun kinases) phosphorylation and activation mediated by activated human TAK1  activated TAK1 mediates p38 MAPK activation  Mitotic Prometaphase  Activated TLR4 signalling  Toll Like Receptor 4 (TLR4) Cascade  Translocation of GLUT4 to the plasma membrane  Centrosome maturation  Recruitment of mitotic centrosome proteins and complexes  TRAF6 mediated IRF7 activation  Toll-Like Receptors Cascades  NOD1/2 Signaling Pathway  MyD88 cascade initiated on plasma membrane  Toll Like Receptor 5 (TLR5) Cascade  Toll Like Receptor 10 (TLR10) Cascade  TRAF6 mediated induction of NFkB and MAP kinases upon TLR7/8 or 9 activation  MyD88 dependent cascade initiated on endosome  Toll Like Receptor 7/8 (TLR7/8) Cascade  Separation of Sister Chromatids  Toll Like Receptor 9 (TLR9) Cascade  Mitotic Anaphase  Mitotic Metaphase and Anaphase  MyD88:Mal cascade initiated on plasma membrane  Toll Like Receptor TLR6:TLR2 Cascade  Interleukin-1 signaling  Toll Like Receptor TLR1:TLR2 Cascade  Toll Like Receptor 2 (TLR2) Cascade  IRAK2 mediated activation of TAK1 complex  IRAK2 mediated activation of TAK1 complex upon TLR7/8 or 9 stimulation  L1CAM interactions  G2/M Transition  Mitotic G2-G2/M phases  MHC class II antigen presentation  IRAK1 recruits IKK complex  IRAK1 recruits IKK complex upon TLR7/8 or 9 stimulation  MAP kinase activation in TLR cascade  Immune System  Factors involved in megakaryocyte development and platelet production  TRAF6 mediated induction of TAK1 complex  Formation of tubulin folding intermediates by CCT/TriC  Hemostasis  IKK complex recruitment mediated by RIP1  RIP-mediated NFkB activation via ZBP1 |
| **ACTG1, ADAM22, CFL1, EPB41L2, KIF5C, KLC3, NCKAP1, OSBPL3, RAB5A, RABEP1, YWHAB, YWHAE, YWHAH, YWHAQ, YWHAZ** | Neurotrophin signaling pathway  Oocyte meiosis  Cell cycle  Pathogenic Escherichia coli infection  Regulation of actin cytoskeleton  Tight junction  Phagosome  Endocytosis | Myometrial Relaxation and Contraction Pathways  Cell cycle  Calcium Regulation in the Cardiac Cell  SIDS Susceptibility Pathways  Pathogenic Escherichia coli infection  Regulation of Actin Cytoskeleton  EGF-EGFR Signaling Pathway | Signaling mediated by p38-alpha and p38-beta  p38 signaling mediated by MAPKAP kinases  Insulin-mediated glucose transport  a6b1 and a6b4 Integrin signaling  Alpha6Beta4Integrin  FoxO family signaling  Stabilization and expansion of the E-cadherin adherens junction  E-cadherin signaling in the nascent adherens junction  E-cadherin signaling events  Trk receptor signaling mediated by PI3K and PLC-gamma  Regulation of p38-alpha and p38-beta  p38 MAPK signaling pathway  Role of Calcineurin-dependent NFAT signaling in lymphocytes  Neurotrophic factor-mediated Trk receptor signaling  BMP receptor signaling  Posttranslational regulation of adherens junction stability and dissassembly  IL1-mediated signaling events  N-cadherin signaling events  CDC42 signaling events  Regulation of CDC42 activity  Regulation of nuclear beta catenin signaling and target gene transcription  Regulation of cytoplasmic and nuclear SMAD2/3 signaling  Regulation of nuclear SMAD2/3 signaling  TGF-beta receptor signaling  TNF receptor signaling pathway  ALK1 pathway  Canonical Wnt signaling pathway  ALK1 signaling events  TNF alpha/NF-kB  p75(NTR)-mediated signaling  Noncanonical Wnt signaling pathway  Wnt signaling network  Glypican 3 network  Syndecan-4-mediated signaling events  AP-1 transcription factor network  Integrin-linked kinase signaling  Signaling events mediated by focal adhesion kinase  Arf6 trafficking events  Class I PI3K signaling events  IFN-gamma pathway  Sphingosine 1-phosphate (S1P) pathway  Alpha9 beta1 integrin signaling events  Endothelins  Signaling events mediated by VEGFR1 and VEGFR2  Insulin Pathway  EGF receptor (ErbB1) signaling pathway  Thrombin/protease-activated receptor (PAR) pathway  ErbB1 downstream signaling  Urokinase-type plasminogen activator (uPA) and uPAR-mediated signaling  Class I PI3K signaling events mediated by Akt  Arf6 downstream pathway  LKB1 signaling events  EGFR-dependent Endothelin signaling events  ErbB receptor signaling network  Glypican 1 network  IGF1 pathway  Syndecan-1-mediated signaling events  Arf6 signaling events  VEGF and VEGFR signaling network  PAR1-mediated thrombin signaling events  mTOR signaling pathway  Signaling events mediated by Hepatocyte Growth Factor Receptor (c-Met)  PDGF receptor signaling network  Nectin adhesion pathway  Internalization of ErbB1  PDGFR-beta signaling pathway  GMCSF-mediated signaling events  IL5-mediated signaling events  S1P1 pathway  IL3-mediated signaling events  Plasma membrane estrogen receptor signaling  TRAIL signaling pathway  Glypican pathway  Beta1 integrin cell surface interactions  Rap1 signalling  Proteoglycan syndecan-mediated signaling events  Integrin family cell surface interactions  Signaling events mediated by HDAC Class II  Signaling by Interleukins  Hemostasis  Regulation of mRNA Stability by Proteins that Bind AU-rich Elements  Signaling events mediated by HDAC Class I  EGFR1  Platelet activation, signaling and aggregation  Signalling by NGF | Activation of BAD and translocation to mitochondria  Translocation of GLUT4 to the plasma membrane  Activation of BH3-only proteins  Intrinsic Pathway for Apoptosis  Membrane Trafficking  Apoptosis  Factors involved in megakaryocyte development and platelet production  Kinesins  VEGFA-VEGFR2 Pathway  EPHB-mediated forward signaling  Hemostasis  Signaling by VEGF  Formation of annular gap junctions  Gap junction degradation |
| **ANK2, CDK5, CDKN1B, CLU, PPARG, PSMC5, PSMD1, STMN1, TAF9, UBA1** | Proteasome | Proteasome Degradation  Parkin-Ubiquitin Proteasomal System pathway  miRNA regulation of DNA Damage Response  DNA damage response  Integrated Pancreatic Cancer Pathway | EGFR-dependent Endothelin signaling events  GMCSF-mediated signaling events  Class I PI3K signaling events  IL5-mediated signaling events  p53-Dependent G1 DNA Damage Response  Urokinase-type plasminogen activator (uPA) and uPAR-mediated signaling  Regulation of DNA replication  ErbB1 downstream signaling  Alpha9 beta1 integrin signaling events  Class I PI3K signaling events mediated by Akt  Signaling events mediated by VEGFR1 and VEGFR2  PDGF receptor signaling network  Endothelins  Thrombin/protease-activated receptor (PAR) pathway  PAR1-mediated thrombin signaling events  Signaling events mediated by focal adhesion kinase  Syndecan-1-mediated signaling events  Sphingosine 1-phosphate (S1P) pathway  SCF(Skp2)-mediated degradation of p27/p21  Removal of licensing factors from origins  Insulin Pathway  VEGF and VEGFR signaling network  Orc1 removal from chromatin  Switching of origins to a post-replicative state  EGF receptor (ErbB1) signaling pathway  Arf6 trafficking events  Signaling events mediated by Hepatocyte Growth Factor Receptor (c-Met)  Cyclin E associated events during G1/S transition  IGF1 pathway  Arf6 signaling events  PDGFR-beta signaling pathway  Internalization of ErbB1  Plasma membrane estrogen receptor signaling  S1P1 pathway  p53-Dependent G1/S DNA damage checkpoint  Cyclin A:Cdk2-associated events at S phase entry  LKB1 signaling events  mTOR signaling pathway  Glypican 1 network  ErbB receptor signaling network  Arf6 downstream pathway  G1/S DNA Damage Checkpoints  IL3-mediated signaling events  Nectin adhesion pathway  IFN-gamma pathway  TRAIL signaling pathway  Glypican pathway  Proteoglycan syndecan-mediated signaling events  Beta1 integrin cell surface interactions  Integrin family cell surface interactions  Synthesis of DNA  G1/S Transition  Cell Cycle Checkpoints  S Phase  Mitotic G1-G1/S phases  C-MYC pathway  HIV Infection  AP-1 transcription factor network  Integrin-linked kinase signaling  DNA Replication  CDC42 signaling events  APC/C:Cdc20 mediated degradation of mitotic proteins  Regulation of ornithine decarboxylase (ODC)  APC/C:Cdc20 mediated degradation of Securin  Regulation of CDC42 activity  p53-Independent DNA Damage Response  Ubiquitin-dependent degradation of Cyclin D1  Validated targets of C-MYC transcriptional repression  Stabilization of p53  Regulation of retinoblastoma protein  CDK-mediated phosphorylation and removal of Cdc6  Regulation of activated PAK-2p34 by proteasome mediated degradation  Vpu mediated degradation of CD4  Cdc20:Phospho-APC/C mediated degradation of Cyclin A  Vif-mediated degradation of APOBEC3G  SCF-beta-TrCP mediated degradation of Emi1  Destabilization of mRNA by AUF1 (hnRNP D0)  Ubiquitin Mediated Degradation of Phosphorylated Cdc25A  Ubiquitin-dependent degradation of Cyclin D  APC/C:Cdh1 mediated degradation of Cdc20 and other APC/C:Cdh1 targeted proteins in late mitosis/early G1  Cell Cycle, Mitotic  Autodegradation of the E3 ubiquitin ligase COP1  p53-Independent G1/S DNA damage checkpoint  Activation of APC/C and APC/C:Cdc20 mediated degradation of mitotic proteins  Autodegradation of Cdh1 by Cdh1:APC/C  Antigen processing: Ubiquitination & Proteasome degradation  Cross-presentation of soluble exogenous antigens (endosomes)  Regulation of APC/C activators between G1/S and early anaphase  Regulation of Apoptosis  CDT1 association with the CDC6:ORC:origin complex  Degradation of beta-catenin by the destruction complex  Signaling by Wnt  Assembly of the pre-replicative complex  APC/C-mediated degradation of cell cycle proteins  ER-Phagosome pathway  Regulation of mitotic cell cycle  Antigen processing-Cross presentation  M/G1 Transition  DNA Replication Pre-Initiation  Developmental Biology  Regulation of mRNA Stability by Proteins that Bind AU-rich Elements  Class I MHC mediated antigen processing & presentation  Signal Transduction  Host Interactions of HIV factors  Apoptosis  p38 MAPK signaling pathway  Metabolism of amino acids and derivatives  Axon guidance  BMP receptor signaling  Metabolism of mRNA  IL1-mediated signaling events  Adaptive Immune System  Mitotic M-M/G1 phases  Metabolism of RNA  TNF receptor signaling pathway  TGF-beta receptor signaling  Regulation of nuclear SMAD2/3 signaling  Regulation of cytoplasmic and nuclear SMAD2/3 signaling  ATM pathway | SCF(Skp2)-mediated degradation of p27/p21  p53-Dependent G1/S DNA damage checkpoint  p53-Dependent G1 DNA Damage Response  G1/S DNA Damage Checkpoints  Cyclin E associated events during G1/S transition  Cyclin A:Cdk2-associated events at S phase entry  Orc1 removal from chromatin  Switching of origins to a post-replicative state  Removal of licensing factors from origins  Regulation of DNA replication  Antigen processing: Ubiquitination & Proteasome degradation  HIV Infection  Synthesis of DNA  DNA Replication  G1/S Transition  Cell Cycle Checkpoints  S Phase  Mitotic G1-G1/S phases  Class I MHC mediated antigen processing & presentation |
| **ANXA2, BAG6, C1QBP, CDH1, CTSB, DCXR, DMWD, ECHS1, GABARAPL2, GLO1, GSPT1, GSTK1, IKBKE, LMNA, NCDN, NCKIPSD, NIPSNAP1, PDXK, PGRMC2, RGS2, SRM, STAC** | beta-Alanine metabolism  Glutathione metabolism |  | Apoptotic cleavage of cellular proteins  Apoptotic execution phase |  |
| **AES, ATPIF1, CBL, CEP76, DGKE, FABP3, GAB1, GABARAP, GFAP, HIST4H4, INPPL1, MAP1LC3C, NBR1, NEDD4, PAICS, PDHA1, PDHB, PIK3CA, PTPRO, RIBC2, RPL18A, SORBS1, SQSTM1, VPS28** | Phosphatidylinositol signaling system  Valine, leucine and isoleucine biosynthesis  Bacterial invasion of epithelial cells  ErbB signaling pathway  Butanoate metabolism  Citrate cycle (TCA cycle)  Insulin signaling pathway  Pyruvate metabolism  Endocytosis  Inositol phosphate metabolism  PPAR signaling pathway  Glycolysis / Gluconeogenesis  Renal cell carcinoma  Chronic myeloid leukemia | Insulin Signaling  Signaling Pathways in Glioblastoma  Senescence and Autophagy  Signaling of Hepatocyte Growth Factor Receptor  Glycolysis and Gluconeogenesis  IL-4 signaling pathway  RANKL-RANK Signaling Pathway  Prostate Cancer  Prolactin Signaling Pathway  MicroRNAs in cardiomyocyte hypertrophy | Signaling events mediated by Stem cell factor receptor (c-Kit)  EPHA2 forward signaling  FGF signaling pathway  EphrinA-EPHA pathway  acetyl-CoA biosynthesis (from pyruvate)  EGFR1  Regulation of pyruvate dehydrogenase (PDH) complex  Pyruvate metabolism  Syndecan-2-mediated signaling events  N-cadherin signaling events  Signaling by Interleukins  Signaling by FGFR  Posttranslational regulation of adherens junction stability and dissassembly  Signaling by EGFR  Signaling events mediated by TCPTP  E-cadherin signaling in the nascent adherens junction  Stabilization and expansion of the E-cadherin adherens junction  Neurotrophic factor-mediated Trk receptor signaling  E-cadherin signaling events  PI-3K cascade  Interleukin-3, 5 and GM-CSF signaling  Reelin signaling pathway  VEGFR1 specific signals  Pyruvate metabolism and Citric Acid (TCA) cycle  Signaling events mediated by VEGFR1 and VEGFR2  GAB1 signalosome  ErbB receptor signaling network  EPO signaling pathway  VEGF and VEGFR signaling network  p75(NTR)-mediated signaling  Presenilin action in Notch and Wnt signaling  IL6-mediated signaling events  Cytokine Signaling in Immune system  Downstream signaling of activated FGFR  Glypican 1 network  Internalization of ErbB1  Integrins in angiogenesis  IL4-mediated signaling events  Fc-epsilon receptor I signaling in mast cells  Nectin adhesion pathway  Signaling events mediated by Hepatocyte Growth Factor Receptor (c-Met)  S1P1 pathway  Signaling events regulated by Ret tyrosine kinase  Class I PI3K signaling events mediated by Akt  Sphingosine 1-phosphate (S1P) pathway  Trk receptor signaling mediated by PI3K and PLC-gamma  IFN-gamma pathway  Endothelins  IL3-mediated signaling events  Arf6 downstream pathway  PDGFR-beta signaling pathway  PDGF receptor signaling network  PAR1-mediated thrombin signaling events  Signaling events mediated by focal adhesion kinase  Arf6 trafficking events  Syndecan-1-mediated signaling events  Urokinase-type plasminogen activator (uPA) and uPAR-mediated signaling  LKB1 signaling events  GMCSF-mediated signaling events  Plasma membrane estrogen receptor signaling  IL5-mediated signaling events  Alpha9 beta1 integrin signaling events  Arf6 signaling events  ErbB1 downstream signaling  EGFR-dependent Endothelin signaling events  Thrombin/protease-activated receptor (PAR) pathway  Immune System  Signaling by SCF-KIT  IGF1 pathway  Insulin Pathway  Class I PI3K signaling events  EGF receptor (ErbB1) signaling pathway  mTOR signaling pathway  TRAIL signaling pathway  Glypican pathway  Beta1 integrin cell surface interactions  Proteoglycan syndecan-mediated signaling events  Integrin family cell surface interactions  LPA receptor mediated events | repression of WNT target genes  Signaling by constitutively active EGFR |
| **ACTR1A, AIFM1, CTSD, CYB5B, DCC, EIF4A2, ERP29, ESD, GNG12, GOT1, H2AFX, HADHA, HLA-B, LMNB1, MAPK1, MCC, PACSIN3, PEBP1, PRDX3, PREP, RPL10A, SPG20, STC2** |  |  | FAS (CD95) signaling pathway  Ceramide signaling pathway  TNF receptor signaling pathway | Antigen Presentation: Folding, assembly and peptide loading of class I MHC  Endosomal/Vacuolar pathway  Class I MHC mediated antigen processing & presentation  ER-Phagosome pathway  Immunoregulatory interactions between a Lymphoid and a non-Lymphoid cell  Antigen processing-Cross presentation  Adaptive Immune System  Interferon gamma signaling  Interferon Signaling  Immune System  Interferon alpha/beta signaling  Cytokine Signaling in Immune system  HDACs deacetylate histones  RMTs methylate histone arginines |
| **BRE, CAPN2, CAPNS1, CNP, GNAI1, GNB1, GNB2, GNB4, LRPPRC, MTNR1A, MTNR1B, RAB1A, SLC25A13, STAU1, SYT5, TERF1, TUBA1A, TUBB3, USP32, USP50, VPS35** | Chemokine signaling pathway  Gap junction  Pathogenic Escherichia coli infection | Calcium Regulation in the Cardiac Cell  G Protein Signaling Pathways  Small Ligand GPCRs  Corticotropin-releasing hormone  Myometrial Relaxation and Contraction Pathways  Nicotine Activity on Dopaminergic Neurons  Pathogenic Escherichia coli infection  Parkin-Ubiquitin Proteasomal System pathway  TSH signaling pathway  Integrin-mediated cell adhesion | Integration of energy metabolism  GABA B receptor activation  Activation of GABAB receptors  Glucagon signaling in metabolic regulation  GABA receptor activation  ADP signalling through P2Y purinoceptor 12  Signal amplification  Inhibition of voltage gated Ca2+ channels via Gbeta/gamma subunits  G-protein activation  G protein gated Potassium channels  Activation of G protein gated Potassium channels  Neurotransmitter Receptor Binding And Downstream Transmission In The Postsynaptic Cell  G alpha (z) signalling events  Inwardly rectifying K+ channels  Nongenotropic Androgen signaling  Transmission across Chemical Synapses  G alpha (s) signalling events  G alpha (i) signalling events  CXCR3-mediated signaling events  Neuronal System  Hedgehog signaling events mediated by Gli proteins  Opioid Signalling  Signaling events mediated by the Hedgehog family  Potassium Channels | Post-chaperonin tubulin folding pathway  Microtubule-dependent trafficking of connexons from Golgi to the plasma membrane  Transport of connexons to the plasma membrane  Protein folding  Formation of tubulin folding intermediates by CCT/TriC  Recruitment of NuMA to mitotic centrosomes  Cooperation of Prefoldin and TriC/CCT in actin and tubulin folding  Gap junction assembly  Kinesins  Recycling pathway of L1  Gap junction trafficking  Gap junction trafficking and regulation  Centrosome maturation  Recruitment of mitotic centrosome proteins and complexes  ADP signalling through P2Y purinoceptor 12  Chaperonin-mediated protein folding  G-protein activation  Signal amplification  Hedgehog 'off' state  G2/M Transition  Translocation of GLUT4 to the plasma membrane  Mitotic G2-G2/M phases  Signaling by Hedgehog  G alpha (z) signalling events  Hemostasis  L1CAM interactions  Adrenaline,noradrenaline inhibits insulin secretion  Resolution of Sister Chromatid Cohesion  Mitotic Prometaphase  MHC class II antigen presentation  M Phase  Regulation of insulin secretion  Opioid Signalling  Factors involved in megakaryocyte development and platelet production  Activation of GABAB receptors  GABA B receptor activation  Cell Cycle  G alpha (i) signalling events  Integration of energy metabolism  Cell Cycle, Mitotic  Separation of Sister Chromatids  Prostacyclin signalling through prostacyclin receptor  GABA receptor activation  G beta:gamma signalling through PLC beta  Presynaptic function of Kainate receptors  Mitotic Anaphase  Mitotic Metaphase and Anaphase  Thromboxane signalling through TP receptor  G alpha (s) signalling events  ADP signalling through P2Y purinoceptor 1  Membrane Trafficking  Neurotransmitter Receptor Binding And Downstream Transmission In The Postsynaptic Cell  Activation of Kainate Receptors upon glutamate binding  Glucagon-type ligand receptors  Thrombin signalling through proteinase activated receptors (PARs)  Glucagon signaling in metabolic regulation  Glucagon-like Peptide-1 (GLP1) regulates insulin secretion  Vasopressin regulates renal water homeostasis via Aquaporins  Orphan transporters  G beta:gamma signalling through PI3Kgamma  Metabolism of proteins  G-protein beta:gamma signalling  Aquaporin-mediated transport  Transmission across Chemical Synapses  Inhibition of adenylate cyclase pathway  Adenylate cyclase inhibitory pathway  Golgi Cisternae Pericentriolar Stack Reorganization  Ca2+ pathway  Platelet activation, signaling and aggregation  Loss of proteins required for interphase microtubule organizationÂ from the centrosome  Loss of Nlp from mitotic centrosomes  Axon guidance  G alpha (12/13) signalling events  Platelet homeostasis  Regulation of PLK1 Activity at G2/M Transition  Activation of G protein gated Potassium channels  Inhibition of voltage gated Ca2+ channels via Gbeta/gamma subunits  G protein gated Potassium channels  Neuronal System  Prefoldin mediated transfer of substrate to CCT/TriC |
| **C19ORF62, COX2, COX5A, COX6B1, COX6C, CSTB, DAZAP2, EIF4H, GZMA, HSPA1A, LASP1, LIN54, MDM2, NDUFA12, NDUFA2, NDUFA4, NDUFA5, NDUFA8, NDUFA9, NDUFB5, NDUFB7, NDUFS1, NDUFS3, PHB, PRDX5, PSMF1, PTPRD, RHOXF2, RNH1, TNIP3, TOLLIP, USP9X** | Parkinson's disease  Oxidative phosphorylation  Alzheimer's disease  Huntington's disease  Metabolic pathways  Cardiac muscle contraction | Electron Transport Chain  Oxidative phosphorylation | Respiratory electron transport  Respiratory electron transport, ATP synthesis by chemiosmotic coupling, and heat production by uncoupling proteins.  The citric acid (TCA) cycle and respiratory electron transport  Metabolism | Respiratory electron transport  Respiratory electron transport, ATP synthesis by chemiosmotic coupling, and heat production by uncoupling proteins.  The citric acid (TCA) cycle and respiratory electron transport  Attenuation phase  HSF1-dependent transactivation  Regulation of HSF1-mediated heat shock response  Cellular response to heat stress  Cellular responses to stress  HSF1 activation |
| **ANK3, ANXA3, ARL15, ARL3, ATP6V1D, BCR, CHMP7, GAPDH, GRIPAP1, IGSF21, LAMA4, LRRK1, MYO5A, NUDT21, PAFAH1B3, RIMS1, RPLP1, SMAD2, TUBB2A, UBR1** |  |  |  |  |
| **ABI2, ATL1, CBR1, CCDC53, CDKN2A, CKB, CPLX2, EIF2C3, EXOC7, GOT2, HGS, HIST1H2BF, IFT20, KIF5B, NAPA, NME2, OXCT1, PCBD1, RTN4, SNAP23, SNAP25, SSBP1, STIP1, SYT1, TFCP2, TUBA1C, TUBB2C, UBQLN1, USPL1, VDAC1, ZDHHC17** | SNARE interactions in vesicular transport  Arginine and proline metabolism | Synaptic Vesicle Pathway  Insulin Signaling | Glutamate Neurotransmitter Release Cycle  Dopamine Neurotransmitter Release Cycle  Norepinephrine Neurotransmitter Release Cycle  Acetylcholine Neurotransmitter Release Cycle  Botulinum neurotoxicity  Serotonin Neurotransmitter Release Cycle  Effects of Botulinum toxin  Membrane Trafficking  GABA synthesis, release, reuptake and degradation  Neurotransmitter Release Cycle  Clathrin derived vesicle budding  trans-Golgi Network Vesicle Budding  Insulin Synthesis and Processing | Post-chaperonin tubulin folding pathway  Formation of tubulin folding intermediates by CCT/TriC  Oxidative Stress Induced Senescence  Cooperation of Prefoldin and TriC/CCT in actin and tubulin folding  Protein folding  Oncogene Induced Senescence  Chaperonin-mediated protein folding  Cellular Senescence  Kinesins  Microtubule-dependent trafficking of connexons from Golgi to the plasma membrane  Transport of connexons to the plasma membrane  Translocation of GLUT4 to the plasma membrane  Cellular responses to stress  Prefoldin mediated transfer of substrate to CCT/TriC  Recruitment of NuMA to mitotic centrosomes  Membrane Trafficking  Gap junction assembly  MHC class II antigen presentation  Senescence-Associated Secretory Phenotype (SASP)  Gap junction trafficking  Recycling pathway of L1  Gap junction trafficking and regulation  Factors involved in megakaryocyte development and platelet production  Serotonin Neurotransmitter Release Cycle  Dopamine Neurotransmitter Release Cycle  Acetylcholine Neurotransmitter Release Cycle  Glutamate Neurotransmitter Release Cycle  Hedgehog 'off' state  Norepinephrine Neurotransmitter Release Cycle  Packaging Of Telomere Ends  RNA Polymerase I Promoter Opening  Signaling by Hedgehog  DNA methylation  GABA synthesis, release, reuptake and degradation  Cell Cycle, Mitotic  M Phase  Centrosome maturation  Recruitment of mitotic centrosome proteins and complexes  Regulatory RNA pathways  PRC2 methylates histones and DNA |
| **ASNS, ATP2A2, CHGB, CSE1L, CUTC, KARS, MGLL, NEFL, NEFM, NR4A2, RAN, SIRT4, SLC1A3, SLC25A3, SLC25A4, SLC25A5, SLC25A6, STRN4, TMEM62, TNF, TNFRSF1B, TRADD** | Amyotrophic lateral sclerosis (ALS)  Calcium signaling pathway  Adipocytokine signaling pathway  Parkinson's disease  Huntington's disease  RIG-I-like receptor signaling pathway  Apoptosis  Dilated cardiomyopathy  Hypertrophic cardiomyopathy (HCM) | Apoptosis  Electron Transport Chain  TNF alpha Signaling Pathway  Synaptic Vesicle Pathway  SIDS Susceptibility Pathways  Alzheimers Disease | Vpr-mediated induction of apoptosis by mitochondrial outer membrane permeabilization  Interactions of Vpr with host cellular proteins  TNF signaling  Host Interactions of HIV factors  HIV Infection  Extrinsic Pathway for Apoptosis  Death Receptor Signalling  TNF receptor signaling pathway  FAS (CD95) signaling pathway  Canonical NF-kappaB pathway  HIV-1 Nef: Negative effector of Fas and TNF-alpha  TNF alpha/NF-kB  Ceramide signaling pathway  Caspase cascade in apoptosis | Vpr-mediated induction of apoptosis by mitochondrial outer membrane permeabilization  TNF signaling  Interactions of Vpr with host cellular proteins |
| **ALCAM, CCT5, CLTCL1, EGFR, HIST3H3, IMMT, KDM1A, NDUFB9, RBBP6, TNFRSF1A, TPT1, UBA52** | Alzheimer's disease  Hepatitis C  Endocytosis  Huntington's disease | Androgen receptor signaling pathway  Integrated Pancreatic Cancer Pathway | Negative regulation of FGFR signaling  Spry regulation of FGF signaling  EGFR downregulation | Downregulation of ERBB4 signaling  Signaling by ERBB4  Signaling by ERBB2 |
| **ARIH2, ENO2, ENSA, HK1, HSF1, LYPLA2, MINK1, RAP2A, RBCK1, SCAMP1, SCMH1, UBE2L3, UBE2N, UBE2S, UBE3A** | Ubiquitin mediated proteolysis  Glycolysis / Gluconeogenesis | Glycolysis and Gluconeogenesis |  | Antigen processing: Ubiquitination & Proteasome degradation  Class I MHC mediated antigen processing & presentation |
| **BIN1, CNBP, COBRA1, CUX1, DLGAP4, DNM1, FBP1, FLT3LG, HSPA8, HSPA9, MYST2, PACSIN1, PFN2, PPID, PPP1R1B, SEPT5** | Parkinson's disease  Endocytosis | Parkin-Ubiquitin Proteasomal System pathway |  |  |
